# Enhanced expression of ADAMTS1 in ovarian carcinomas: loss of ADAMTS1 expression instigates cellular reprogramming of extracellular matrix ensuing altered plasticity, augmented migration and attenuated adhesion

**DOI:** 10.1186/s12929-026-01260-z

**Published:** 2026-06-22

**Authors:** Ruth M. Escalona, Dylan King, Noor A. Lokman, Martin K. Oehler, Carmela Ricciardelli, George Kannourakis, Nuzhat Ahmed

**Affiliations:** 1 Fiona Elsey Cancer Research Institute, Suites 23, 106-110 Lydiard Street South, Ballarat Technology Park Central, Ballarat, VIC 3353 Australia; 2 Centre for Endocrinology and Reproductive health, Hudson Institute of Medical Research, Department of Translational Medicine, Monash University, VIC 3168, Australia; 3 School of Medicine, College of Health, Adelaide University, Adelaide, SA 5000 Australia; 4 Department of Gynecological Oncology, Royal Adelaide Hospital, Adelaide, SA 5000 Australia; 5 Health Innovation and Transformation Centre, Federation University Australia, Mt Helen Campus, Ballarat, VIC 3350 Australia; 6 Department of Obstetrics and Gynaecology, University of Melbourne, Parkville, Melbourne, VIC 3052 Australia; 7 Department of Surgery, St Vincent Hospital, University of Melbourne, Fitzroy, Melbourne, VIC 3065 Australia

**Keywords:** Ovarian cancer, ADAMTS1, MET, MMP9, Versican, Cdc42, Cellular plasticity, Proliferation, Migration, Adhesion

## Abstract

**Background:**

ADAMTS1, a disintegrin and metalloproteinase with thrombospondin motif 1, plays a role in inflammation, organogenesis, and ovulation. Up or downregulation of ADAMTS1 has been implicated in tissue remodelling leading to cancer. We analysed the expression of ADAMTS1 in different stages/grades of primary and metastatic serous ovarian tumours and ascites-derived tumour cells from patients and assessed the functional role of ADAMTS1 in ovarian cancer (OC) cell lines.

**Methods:**

The expression and localisation of ADAMTS1 was assessed by immunohistochemistry (IHC) and Opal Multiplex IHC staining of OC tissues. Functional roles of ADAMTS1 in OC cell lines were assessed by using siRNA-mediated knockdown (KD), MTT assay, cell migration by xCELLigence, cell adhesion, ELISA, qRT-PCR, Western blot, immunofluorescence (IF) and activity of Cdc42 GTPase.

**Results:**

The expression of ADAMTS1 was significantly enhanced in higher stages/grades of ovarian tumours compared to benign tumours. In high-grade tumours, ADAMTS1 was localised more in the nucleus of epithelial cells while localisation in stromal cells was mostly in the cytoplasm. Significantly higher mRNA expression of ADAMTS1 was noted in epithelial compared to mesenchymal ascites-derived tumour cells. The expression of ADAMTS1 was significantly higher in metastatic high-grade tumours compared to primary tumours.

KD of ADAMTS1 expression by siRNA in OC cell lines had no effect on cell proliferation but resulted in decreased cell adhesion, increased cell migration accompanied by increased expression of markers (CDH1 and EPCAM) associated with epithelial plasticity. Remodelling of ECM accompanied by increased intra- and extracellular production of VCAN and enhanced Cdc42 GTPase activity was also noted in cell lines with ADAMTS1 KD. Cdc42 GTPase specific inhibitor, ML141, reversed ADAMTS1 KD-mediated enhanced migration. On the other hand, VCAN KD inhibited ADAMTS1 KD-mediated migration and reversed the effect on cell adhesion.

**Conclusions:**

These results suggest that the expression of ADAMTS1 progressively enriches ovarian tumours and promotes OC progression. Its knock down in in vitro cell culture impacts ECM remodelling through enhanced Cdc42GTPase activity and VCAN production resulting in epithelial cell plasticity, accelerated migration, and reduced cell adhesion.

**Supplementary Information:**

The online version contains supplementary material available at 10.1186/s12929-026-01260-z.

## Introduction

Cancer of the ovary (ovarian cancer, OC) is the eighth most common cancer, amongst the gynaecological cancers, affecting women world-wide [1]. The cancer is insidious with a five-year survival rate of ~ 45% after initial diagnosis (https://www.cancer.org.au/cancer-information/types-of-cancer/ovarian-cancer). The physiopathology of epithelial OC, which originates either from the surface epithelium of ovaries or from the fallopian tubes is still poorly understood [2]. The tumour microenvironment (TME) of OC is unique as it comprises both solid tumours and floating tumour cell aggregates residing in ascites, sloughed either from the surface epithelium of the ovaries or the epithelium of fallopian tubes [3, 4]. These tumours grow and disseminate within the abdominal cavity in ascites, the volume of which can range from few to several litres [5]. The shear pressure of ascites and the confinement of the abdominal cavity under hypoxic conditions produces a unique hostile microenvironment for the expanding tumour cells prompting them to reprogram extracellular matrix (ECM) and associated tumour cell plasticity to promote metastasis [6–8]. This complex interaction of OC cells responding to different stimuli within the TME at different stages of OC progression enables ECM remodelling, facilitating altered cellular functions such as adhesion and migration, to promote metastases, the primary cause of cancer-related death in patients [9].

The ADAMTS (a disintegrin and metalloproteinase with thrombospondin motifs) are a family of zinc metalloproteinases each possessing a catalytic domain which contains the metalloproteinase and disintegrin-like characteristics important for biological action [10, 11]. In normal biological processes, ADAMTS1, is essential for normal growth, fertility, and organ morphology and is implicated in inflammation, organogenesis, and ovulation [11]. ADAMTS1 is one of the proteoglycanases that can cleave a variety of proteoglycans such as versican (VCAN), aggrecan, brevican as well as other proteins involved in ECM remodelling [12, 13], during cancer or disease development and progression [10, 11]. Adamts1-/- knock out mice demonstrated high rate of multiple organ defects, including severe kidney and cardiac defects, and the surviving female mice suffered infertility due to ineffective cleavage of versican during ovarian maturation [11]. In that scenario, we can envisage that loss of ADAMTS1 expression, can result in intracellular VCAN accumulation, which may install ADAMTS1 dependent changes in cellular functions. VCAN independently has been shown to promote highly invasive and motile phenotypes in prostate and OC cells [14–16]. A recent study has shown secretory VCAN-dependent motility of normal fallopian tube cells during the ovulatory process, indicating that secreted VCAN is essential for ovulation [17].

Another binding partner of ADAMTS1 is ubiquitously expressed heparan-sulfate proteoglycan, syndecan-4 (SDC4), which acts as a key mediator of several cellular processes including adhesion and migration/invasion [18]. Syndecan-4 through its heparan-sulfate glycosaminoglycan chains provides binding sites for heparin-binding growth factors such as fibroblast growth factors (FGFs), platelet-derived growth factors (PDGFs) and vascular endothelial growth factor (VEGF) [19]. The binding of these growth factors to SDC4 facilitates bioavailability of these growth factors to cells initiating a chain of signalling cascades which regulates different cellular processes [18, 20].

Out of the 19 secreted proteases belonging to the ADAMTS family, ADAMTS1 dysregulation has been linked to ovarian, colorectal, prostate, breast, liver and non-small lung carcinoma [11]. However, there has been several conflicting reports of ADAMTS1 expression in different tumours. For example, studies have demonstrated a decreased expression of ADAMTS1 in lung and breast tumours compared to normal tissues [21, 22], while others, including OC, the opposite was observed [23, 24]. In these studies, the decreased or increased expression of ADAMTS1 in tumours was associated with a higher degree of malignancy contributing to cancer development and progression.

In addition to its proteolytic function, ADAMTS1 interacts with a variety of cytokines, growth factors and its receptors [10]. ADAMTS1 interacts with latency associated peptide-TGFβ (LAP-TGFβ) to promote TGFβ activation [10, 25] and fibulin-1 (FBLN1) [26]. In breast cancer ADAMTS1 facilitates the ectodomain shedding of transmembrane bound epidermal growth factor (EGF) and EGF-like ligands, liberating active mitogens to bind to their corresponding receptor to promote tumour metastasis [27].

ADAMTS1 has recently been associated with epithelial mesenchymal transition (EMT) in different cancers. Overexpression of ADAMTS1 in lung cancer cell lines promoted EMT-like phenomenon in vitro and in in vivo [28]. Subcutaneous injection of recombinant ADAMTS1 in lung cancer cell lines led to enhanced lung metastasis compared to control [28]. Conversely, knock down of ADAMTS1 in this model impaired migration and EMT [28]. Mechanistically, ADAMTS1 in this model promoted EMT through the activation of TGFβ signalling, as pharmacological inhibition or siRNA mediated downregulation of TGFβ opposed EMT driven by ADAMTS1 [28]. In oral squamous cell carcinoma model, cyclic increase in ADAMTS1-L1 cell adhesion molecule (L1CAM) axis mediated epidermal growth factor (EGFR) activation leading to EMT-mediated excessive invasive abilities of oral squamous carcinoma cell lines [29]. In a renal carcinoma model, ADAMTS1–VCAN–EGFR axis was shown to drive anoikis resistance and invasion [30]. These studies highlight the role of ADAMTS1 in regulating EMT in conjunction with other signalling receptors.

The effects of ADAMTS1 on cellular adhesion, migration and invasion have also been a subject of conflicting results. In lung cancer, cell migration and invasion were positively correlated to ADAMTS1 expression [28], while in breast [22] and OC [31] low ADAMTS1 expression was associated with enhanced cell migration and invasion. The downregulation of ADAMTS1 in breast cancer cells led to upregulated levels of VEGF in the conditioned medium which promoted cell migration and invasion [22]. However, in OC this effect seems to be specific for the cell type tested, as complete knock down of ADAMTS1 modulated the Cdc42 activity which enhanced migration [31]. On the other hand, ADAMTS1 cleavage of syndecan-4 has been shown to impair cell adhesion in different cellular systems [18].

The aim of the present study was to elucidate the expression of ADAMTS1 in different stages and grades of serous ovarian tumours and to understand the functional role of ADAMTS1 in OC cells by knocking down the expression of the protein. Our study for the first time reveals a high expression of ADAMTS1 in ovarian tumours compared to normal/benign ovaries, consistent with an *in-silico* analysis, and that correlated with shorter overall survival in OC patients with ADAMTS1^high^ compared to ADAMTS1^low^ expression. We also show that the expression of ADAMTS1 was significantly enhanced in metastatic (omentum) ovarian tumours compared to primary ovarian tumours. In in vitro cell culture studies, a loss of ADAMTS1 expression by siRNA (A-KD) resulted in decreased mRNA expression of SDC4, FBLN1, LICAM and ECM-associated integrins consistent with ECM remodelling effects of A-KD [18, 29]. This resulted in decreased adhesion but increased migration and altered cell plasticity in some OC cell lines. On the other hand, ADAMTS1 siRNA enhanced Cdc42GTPase activity and VCAN expression in OC cells. Knock down of VCAN or treatment with Cdc42 GTPase-specific inhibitor in A-KD cells reversed the A-KD mediated enhanced migration; while only VCAN knockdown reversed the decreased adhesion observed in the A-KD OC cell lines. However, these phenomena in relation to A-KD were not observed in the ES2 cell line which had several-fold high expression of ADAMTS1 than other OC cell lines. These results suggest that the expression level of ADAMTS1 in OC cells dictates ECM-mediated cellular reprogramming resulting in tumour cells with modified functional characteristics.

## Materials and methods

### Research sample collection

Ovarian tumours were acquired from OC patients admitted to The Royal Women’s Hospital (RWH) after procuring written consents under officially accepted ethical approval (Ethics approval #09/09) by the Research and Ethics Committee of Royal Women’s Hospital, Melbourne, Australia. High-grade serous epithelial ovarian tumours were obtained from seventeen patients undergoing surgery at the Oncology Dysplasia Unit of The Royal Women’s Hospital, Melbourne, Australia. Eight normal/benign samples were obtained from patients undergoing abdominal hysterectomy or bilateral salpingo-oophorectomy due to pre-existing medical conditions. At the time of collection, tissues were fixed in 4% paraformaldehyde. Information on tumour grade, stage and histopathology for individual tumour was obtained from pathology reports. The clinical information on each tumour sample is described in Supplementary Table 1. Patients recruited in this study did not undergo treatment with chemotherapy, immunotherapy, or radiation. This study was also approved for Fiona Elsey Cancer Research Tissue Bank by Grampians Health and St John of God Hospital, Ballarat, Human Research Ethics Committee (Project ID:37521).

Primary high-grade serous ovarian cancer (HGSOC) (n = 120) and metastatic HGSOC (n = 49) were collected with patient consent and approval by the Royal Adelaide Hospital Human Ethics Committee (RAH protocols #060–903 and #080102). The formalin fixed and paraffin embedded (FFPE) tumour samples were assembled in tissue microarrays (TMA). Clinical information of these patients is listed in Supplementary Table 2. Each patient tumour had duplicate or triplicate 1 mm diameter cores.

Immunohistochemistry staining of tumours was outsourced to the Anatomical Pathology Laboratory Services at The Royal Children’s Hospital, Melbourne, Australia. Briefly, paraffin embedded tissue samples were sectioned at 4 μm thickness and stained using ADAMTS1 antibody (1:100, ab39194, Abcam, Taipei, USA), or CA125 (1:1, Clone OC125 Cat#760–2610, Ventana Medical Systems, Inc., Arizona, USA) and OptiView DAB IHC Detection kit (Ventana Medical Systems, Inc., Arizona, USA). The samples were processed on Ventana Benchmark Immunostainer (Ventana Medical Systems, Inc., Arizona, USA) as described previously [32]. Negative controls used in this study were prepared by incubating samples in diluent without primary antibodies followed by the secondary antibody. Sections of human placental and tonsil tissues were used as positive controls to determine the staining efficiency of the antibodies used. Stained slides were then scanned at X40 magnification by the Southern Health Tissue Bank at Monash Medical Centre (Victoria, Australia) using the Aperio Scanscope XT (Aperio-Leica Microsystems Pty Ltd) and imaged using the Aperio ImageScope v12.3.2.8013 software (Leica Biosystems Pathology Imaging 2003–2016). Sections were evaluated microscopically for positive DAB staining in conjunction with positive CA125 (Ventana Medical Systems, Inc., Arizona, USA) staining, as described previously [33, 34]. Three to eight random areas were selected and DAB positivity over each of these areas was calculated and divided by the average of negative control of each group. To deduce epithelial and stromal intensity of ADAMTS1 staining, three to five different areas of only epithelial or stroma staining were selected from each block and assessed by FIJI software [ImageJ software 1.51j8 (Wayne Rasband National Institute of Health, USA; http://imagej.org)]. The DAB intensities were assessed and was divided by the area of analysed section. Results were plotted on a bar graph using GraphPad PRISM software (Version 10.4.1).

Immunostaining of the TMAs were performed at Adelaide University using the same ADAMTS1 antibody (1:500, ab39194, Abcam) as described previously [35]. Slides were subsequently incubated with biotinylated goat anti-rabbit (1:400, Dako, Mulgrave, VIC, Australia) and streptavidin-horse radish peroxidase (HRP) (1:500, Dako, Australia) each for 1 h at room temperature. Peroxidase activity was detected using the substrate diaminobenzidine (DAB)/H_2_O_2_, as described previously [35]. Positive controls for the immunostaining included mouse ovary and negative controls included tissue sections incubated with no primary antibody or rabbit IgG isotype control (Thermo Fisher Scientific, Melbourne, Australia). The TMA slides were scanned using the NanoZoomer Digital Pathology System (Hamamatsu Photonics, Japan). ADAMTS1 immunostaining in both the cancer cells and cancer associated stroma cells (5–6 areas per tissue) was measured as H-score (0–300), a measure of both the percentage of positive cells, and the intensity of positive staining using QuPath software (Version 0.4.3) [36].

### Collection of ascites and ascites-derived tumour cells

Ascites samples were obtained from high-grade serous OC patients undertaking treatment at either RWH, Melbourne, Australia or at Monash Medical Centre, Clayton, Australia. Chemo-naïve ascites was collected at the time of diagnosis as described previously [37, 38]. Tumour cells (epithelial) were separated from stromal (mesenchymal) cells in ascites using the method described previously [37]. Clinical information on each patient from whom ascites was collected is described in Supplementary Table 3.

### Accessing online databases

The Human Protein Atlas Version 25.0 (https://www.proteinatlas.org, accessed on 8 February 2026) [39] was used to obtain mRNA expression overview of ADAMTS1 in RNA-seq data from The Cancer Genome Atlas (TCGA) across different cancer types (https://www.proteinatlas.org/ENSG00000154734-ADAMTS1/cancer). This website was also used to obtain the protein expression of ADAMTS1 in OC cell lines (n = 59) (https://www.proteinatlas.org/ENSG00000154734-ADAMTS1/cell+line#ovarian_cancer). The mRNA expression data is represented as pTPM, is a normalized transcripts per million in reference to Human Protein Atlas (HPA). It is a quantitative comparison of transcript abundance across different human tissues. While the protein expression is based on the mass-spectrometry (MS) proteomic dataset from the Pan-Cancer Atlas project (obtained by SCIEX6600 Triple TOF mass spectrometry running DIA mode) is represented by a circle for each cell line. The size of the circle corresponds to the Normalized Relative Protein Expression (nRPX) value for the cell lines, and the colour represents the tissue type to which the cell line is related.

GENT2, http://gent2.appex.kr/gent2/ [40] was used to compare mRNA levels in normal tissues (ovarian surface epithelium, OSE, n = 66; fallopian tube, FT, n = 40) and HGSOC (n = 806). Individual samples were reviewed and specified as normal ovarian surface epithelium (OSE), fallopian tube (FT) or HGSOC subtype as described previously [41].

KM Plotter (http://www.kmplot.com, accessed on 21 April 2026) was used to obtain the overall survival (OS). KM graphs were derived from two distinct databases mRNA-ChIP-seq [42] & Pan-cancer RNA-seq [43]. The above analyses were performed using ADAMTS1 (Affymetrix ID 222162) as the gene of interest on grade 3, ovarian tumours. The mRNA-ChIP database had 1015 samples [41]. The expression probed used in the analysis had a range of 25–14,141 units, and the auto select best cutoff value used in analysis was 2297. This was used to differentiate between high ADAMTS1 expressing tumours (samples  > 2297, n = 307) versus low ADAMTS1 expressing tumours (samples   < 2297, n = 708).

The second analysis used the mRNA-seq Pan-cancer database selected for grade 3 ovarian tumours, only had 319 samples. The expression probed used in the analysis had a range of 85–16,818 units, and the auto select best cutoff value used in analysis was 2098. This was used to determine high ADAMTS1 expressing tumours (samples < 2098, n = 146) versus low ADAMTS1 expressing tumours (samples > 2098, n = 173).)

### Cell culture

Three established ovarian cancer cell lines were used in this study including OVCAR4, OVCAR5 and ES2. OVCAR4 cell line derived from the ascites of a patient diagnosed with ovarian serous adenocarcinoma, pre-treated with cyclophosphamide, cisplatin, and doxorubicin chemotherapies (Cellosaurus cell line, CVCL_1627) was obtained from Professor David Bowtell (Peter MacCallum Cancer Centre, Parkville, Australia). OVCAR5 (Cellosaurus cell line, CVCL_1628), collected from the ascites of an untreated, advanced stage ovarian cancer patient, was later predicted to be of gastric origin by gene expression analysis [44]. However, a recent paper has confirmed this cell line as of ovarian origin by short tandem repeat (STR) sequencing [45]. ES2 (primary tumour cells derived from a patient with clear cell carcinoma of the ovary, (Cellosaurus cell line, CVCL_3509) was reported to be a clear cell carcinoma cell line in origin but later was identified as high grade serous [46]. OVCAR-5 and ES2 cell lines were kindly donated by Dr. Carmela Ricciardelli (Adelaide University, Adelaide, SA, Australia). Both OVCAR5 and ES2 cell lines were verified by STR in 2021 (Griffith University, Australia).

All three cell lines were maintained in RPMI-1640 (Sigma-Aldrich, Sydney, Australia) and supplemented with L-glutamine (2 mM), and antibiotics (Fungizone, streptomycin and penicillin 1% v/v) and FBS (10% v/v). All Cell lines were maintained at 37 °C in 5% CO_2_ and passaged at least twice a week once they reached a confluence of 65–80%. Details of the other cell lines used in the study are provided in Supplementary Table 4.

### Transient transfections and treatment of cell lines

Three unique 27mer small interfering RNA (siRNA A, B, C) duplexes directed against human ADAMTS1 (Ori- Gene Technologies, SR322803, MD, USA) and a pooled siRNA (A + B + C) directed against ADAMTS1 were used to knock down ADAMTS1 expression (A-KD) in OVCAR4, OVCAR5 and ES2 cell lines. A Universal nontargeting siRNA duplex was used as a Control (C) (OriGene Technologies, SR30004, MD, USA) in these experiments. To avoid off-target effects, the lowest ADAMTS1 siRNA concentrations were optimized for each cell line (range tested was from 1 to 10 nM) and transfected cells were collected for RNA analysis 48 h after transfection. Transfection efficiency for each cell line was evaluated by using 15 nM siGLO™ Red Transfection Indicator (Horizon Discovery (Dharmacon), Lafayette, CO) as per manufacturer’s instructions. Parental cells (P) were cells treated with transfection reagent but without the siRNAs.

Control and knockdown cells were treated with Cdc42/Rac1 GTPase inhibitor ML141 (MERCK, Darmstadt, Germany) at concentrations of 5, 10, 20 and 40 µM for 24 h at 37 °C in 5% CO_2_.

For VCAN knockdown, a 21mer small interfering RNA (siRNA) directed against human VCAN gene that targeted exon 1 (N-terminal region of the VCAN gene) (Ambion, CA, USA) was used at a concentration of 3 nM per transfection for 48 h. For double knock down cells were either transfected with control siRNA or ADAMTS1 siRNA for 24 h followed by VCAN siRNA for 24 h, after which media was changed and cells left to grow for another 24 h before collection.

### OPAL multiplex immunofluorescence staining of tumours

The Opal Multiplex staining was performed on HGSOC (n = 2) and benign ovarian tumour (n = 1) FFPE sections. The staining was conducted by Monash Histology Platform as described previously [47]. Briefly, the antibodies used were E-Cadherin (1:200, ab40772, Abcam; visualized with OPAL Dye 520); ADAMTS1 (1:100, ab39194, Abcam; visualized with OPAL Dye 570) and vimentin (1:200, MA5-35320, Invitrogen; visualized with OPAL Dye 690). Antigen retrieval was done at 98 °C for 30 min in DAKO PT Link in 1× Low pH Target Retrieval Solution (DAKO, CA, USA). Slides were placed on a DAKO Autostainer Plus, followed by Real Peroxidase Blocking (DAKO, CA, USA) and Serum Free Protein Block (DAKO, CA, USA). The sections were stained with blocking buffer diluted with primary antibody for 1 h. After appropriate washing steps, secondary antibody: EnVision System-HRP Labelled Polymer Anti-Rabbit (DAKO, CA, USA) was applied followed by treatment with OPAL Dye working solution (Akoya Biosciences, MA, USA). This was followed by counterstaining with DAPI (1:10,000, Sigma-Aldrich, St Louis, Missouri, USA). Finally, after additional washing steps at room temperature, slides were cover slipped with Prolong Gold anti-Fade reagent (Thermo Fisher Scientific, CA, USA). Slide scanning and imaging was performed on the Olympus VS120^®^ Virtual Slide Microscope System. Scans were then analysed using the image processing software, QuPath (version 0.5.1) [36].

### Immunofluorescence

Immunofluorescence analysis was conducted on cell lines as described previously [34]. Briefly, 1 X 10^4^ cells were cultured overnight on 8-well chamber slides (Lab-Tek II Chamber Slide System, Thermo Fisher Scientific, CA, USA) in complete growth medium at 37 °C in 5% CO_2_. Next day the cells were fixed with paraformaldehyde (PFA)/PBS solution, permeabilized using 0.1 (v/v) Triton X-100 (Sigma-Aldrich) in PBS, washed with cold PBS, and incubated for 2 h with blocking buffer (1% BSA/PBS) followed by primary antibody treatment overnight at 4 °C (Rb ADAMTS-1 Cat # ab39194, Abcam (1:1000); Ms E-Cad Cat#14472S cell signalling (1:200); Ms N-Cadherin, ab98952, Abcam (1:200); Mouse IgG, ab37355, Abcam (1:200); Rabbit IgG ab172730, Abcam(1:200)). Cells were stained with appropriate secondary antibodies (1:200 Alexa fluor 568 Donkey anti-mouse or Alexa fluor 488 Donkey Anti-rabbit, Thermo Fisher Scientific, CA, USA) in blocking buffer for 2 h. DAPI (4′,6-diamidino-2-phenylindole) (Invitrogen, Carlsbad, USA) was used to stain cellular nuclei at a 1:2000 dilution for 10 min at room temperature. Fluorescence imaging was visualized using an OLYMPUS BX53F upright microscope (Olympus, Tokyo, Japan) and images were taken using an Olympus DP70 camera and the Olympus Cells Sens Dimension version 1.7.1 software (Olympics Corporation). To avoid biased measurements, equal intensity acquisition parameters were set prior to imaging [for example the fluorophores DAPI acquisition time was set at 10 milli seconds (ms), Alexa 488 at 300 ms, Alexa 568 at 150 ms and Alexa 594 at 450 ms]. The intensity units were measured using FIJI analysis software [ImageJ software 1.51j8 (Wayne Rasband National Institute of Health, USA; http://imagej.org] according to DAPI location. This was repeated at least 4–9 times for each photograph and at least two images were taken for each well.

## MTT assay

Cell proliferation was assessed by the MTT assay as described previously [34]. Briefly, cells (3X10^4^) were seeded and transfected in 96-well plates on the same day. After 24 h, cell culture medium was replaced with 100 μL of 3-(4,5-dimethylthiazol-2-yl)−2, 5-diphenyltetrazolium bromide) (MTT) solution (Sigma- Aldrich, Melbourne, Australia) dissolved in 1 × PBS solution (0.5 mg/mL) (Sigma-Aldrich, Melbourne, Australia). After 2 h incubation, cell culture medium was replaced with 100 μL of dimethyl sulfoxide (DMSO). Absorbance was read at OD595nm using the CLARIOstar Plate Reader (BMG Labtech, Germany) and MARS Data Analysis Computer Software (BMG Labtech, Mornington, Victoria, Australia).

### Migration assay

Cell migration assay was performed by using the Roche xCELLigence DP instrument as described previously [32, 34]. Briefly, the upper chamber of the 16-well CIM plate (Roche, NSW, Australia) was coated with Matrigel and set for 30 min at 37 °C. Cells suspension (4X10^4^) in Gibco^®^ Opti-MEM™ Media (Thermo-Fisher Scientific, NSW, Australia) and ± ML-141 (MERCK, Darmstadt, Germany) (5, and 10 µM concentrations) were seeded on the top compartment of the pre-equilibrated 16-well CIM plate (Roche, NSW, Australia). Readings were taken every 15 min for ~ 40 h. Each plate contained two duplicate wells. Mean values from each C and ADAMTS1-KD cell lines ± ML-141 are illustrated graphically using PRISM software. Linear regression analysis of two slopes arising from control (C) and ADAMTS1-KD (A-KD) cells were used to obtain significant values.

### Adhesion assay

Adhesion assays were performed as described previously [48]. Briefly, 96-well plates were coated with poly-L-lysine (Sigma, St Louis, Missouri,) and incubated overnight at 4 °C. Plates were then washed twice with PBS, then incubated at 37 °C with 0.5% BSA blocking buffer for 45–60 min. Then washed with PBS and chilled in ice till ready to use. Following treatment of cells as described earlier, cells were trypsinised and harvested in Gibco^®^ Opti-MEM™ Media (Thermo-Fisher Scientific, NSW, Australia). Cells (2X10^4^) were plated in triplicate on the poly-L-lysine treated plates and incubated at 37 °C in 5% CO_2_ for 90 min. Cells were then washed two–three times with PBS to remove non-adhering cells, and the adherent cells were fixed with 100% methanol for 5 min at room temperature. Cells were stained with 0.5% crystal violet for 15 min, then washed three times with PBS and dried at room temperature. Absorbance was then read at OD595 nm using the CLARIOstar Plate Reader (BMG Labtech, Victoria, Australia) and MARS Data Analysis Computer Software (BMG Labtech, Victoria, Australia).

### RNA extraction and relative real-time PCR (qRT-PCR)

RNA was extracted from parental, control, ADAMTS1-KD (± ML141 (MERCK, Darmstadt,Germany) treatment) and VCAN-KD cell lines using TRIzol^®^ reagent (Ambion-Life Technologies, Carlsbad, CA, USA) followed by the chloroform: phenol method as described previously [34]. Five hundred ng of total RNA was reverse transcribed using the high-capacity cDNA Reverse Transcription Kit (Applied Biosystems, CA, USA) and qRT-PCR amplification was performed using the Applied Biosystems ViiA 7 Real-Time PCR (Thermo Fisher Scientific, NSW, Australia) as described previously [34]. Supplementary Table 5 lists the sequences and accession numbers of genes analysed. Data are presented as relative expression normalized to housekeeping gene 18S.

### Western blot

Western blot was performed on OC cell lysates using sodium dodecyl-sulphate polyacrylamide gel electrophoresis (SDS-PAGE) by the methods described previously [32]. Briefly, total protein (15 µg) was separated on 4–15% pre-cast gradient SDS-PAGE gels (Bio-Rad, Sydney, Australia) and transferred to a polyvinylidene difluoride (PVDF) membrane. After blocking with 5% skimmed milk or 3% BSA, the membranes were stained with Erk, P-Erk antibodies (Thermo Scientific and Cell Signalling, Melbourne, Australia, 1:250–1000), followed by treatment with anti-rabbit/mouse IgG (H + L) HRP secondary conjugated antibodies (1:5000) (Bio-Rad, Sydney, Australia). Protein bands were developed using the enhanced chemiluminescence reagents (Bio-Rad Laboratories, Melbourne, Australia) and visualised using the ChemiDoc imaging system (Bio-Rad, Australia). Using densitometric Image Lab 6.0 software (Bio-Rad, Sydney, Australia), the bands for each image were quantified.

### Human ELISA for measurement of ADAMTS-1, syndecan-4 and versican

ELISA (enzyme-linked immunosorbent assay) was performed using the commercially available kits: ADAMTS1 (Human ELISA Kit; Cat#KA4465, Abnova, Taiwan); Versican (Human ELISA Kit, Cat# ab283883, Abcam, Victoria. Australia), and Syndecan-4 (Human ELISA Kit, Cat#EH442RB, Thermo Fisher, NSW, Australia), as per manufacturer’s instructions. Briefly, 2X10^6^ cells were plated in a T75cm-flask and transfected on the same day, followed by changing medium after 24 h, 10 ml of serum free medium (Gibco^®^ Opti-MEM™ Media (Thermo-Fisher Scientific, NSW, Australia). After 48 h post transfection conditioned medium was then collected, span for 5 min at 1200 rpm and cell pellet discarded. Conditioned medium was aliquoted and stored at −20 ^o^C till ready to use. For each ELISA kit appropriate standards were prepared as described by the manufacturer. Standards and 100 μL of conditioned serum-free medium or 50 mg of cell lysates were added to individual 96-well ELISA plates in duplicate and incubated overnight at 4 ºC with gently shaking. After Wash Buffer treatment, biotin conjugate was added and incubated for 1 h, followed by Streptavidin–HRP treatment for another 45 min. After washing steps TMB developmental substrate (3,3',5,5'-tetramethylbenzidine) was added and incubated in the dark for the development of colour specific for each ELISA developed. Following addition of Stop Solution, plates were read at OD450 nm using the CLARIOstar Plate Reader (BMG Labtech, Germany) and MARS Data Analysis Computer Software (BMG Labtech, Mornington, Victoria, Australia). Standard curves for each ELISA were used to then determine the correct concentration of the antigens in individual sample.

### Statistical analysis

When only two treatment groups were compared, an unpaired Mann–Whitney’s non-parametric t-test was used. However, when more than two treatment groups were compared a One-Way ANOVA was used. Data are presented as mean ± standard error of the mean (SEM). xCELLigence data was analysed by linear regression analysis and presented as the standard deviation (SD) of the mean. For statistical significance, the probability levels adopted were p < 0.05(*), p < 0.01(**), p < 0.001 (***) and p < 0.0001 (****). All data were analysed by Graph Pad PRISM software (Version 10.4.1) and Microsoft Excel 2016. All experiments were performed for a minimum of three times (unless otherwise indicated) in triplicate.

## Results

### The expression of ADAMTS1 is significantly higher in human high-grade serous ovarian tumours compared to benign tumours.

**Fig. 1:**
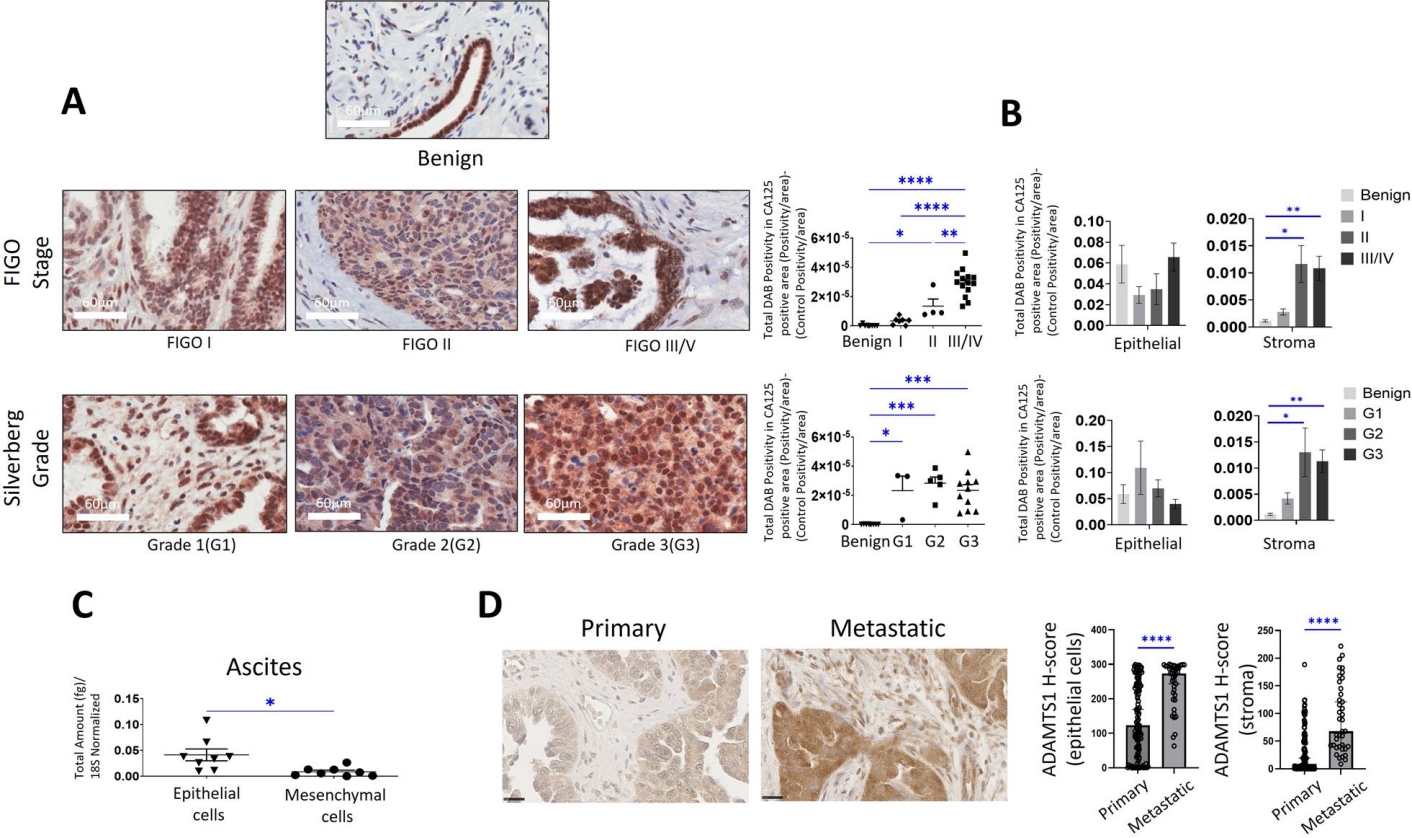
Expression and localization of ADAMTS1 in serous ovarian tumours. **A** Representative image of ADAMTS1 staining in primary serous ovarian and benign serous tumours; divided into FIGO classification (FIGO I, II and III/V) and Silverberg/Shimizu Grading (Grade 1, Grade 2 and Grade 3) classifications. Images are representative of tumours within each group/classification as described in Supplementary Table 1. Graphs represent total positivity of DAB staining within the area that was CA125 positive excluding control DAB positivity. Magnification (40X), scale bar = 60 µM. **B** The stromal staining was selected for CA125 negative areas. Graphs are representative of DAB positivity over area divided by negative control for each patient tissue block described in Methods. **C** mRNA expression of ADAMTS1 in epithelial (n = 8) and mesenchymal (n = 8) populations of cells isolated from ascites. **D** Representative image of ADAMTS1 staining in primary (n = 115) and metastatic (omentum) (n = 42) ovarian tumours. Error bars are presented as median with 95% CI. Significance is indicated by * p < 0.05, ** p < 0.01, *** p < 0.001, **** p < 0.0001, one-way ANOVA or Mann–Whitney t-test

Twenty-five paraffin embedded tissues (Supplementary Table 1), consisting of 8 benign serous ovarian tumours and 17 serous tumours of different stages/grades were analysed by immunohistochemistry using an anti-human ADAMTS1 specific antibody. ADAMTS1 expression was noted in epithelial tumour cells expressing CA125, and some stromal expression of ADAMTS1 was also noted (Fig. 1**A**, Supplementary Figs. 1**A** and **B**). Staining was mostly confined to the cytoplasm and nucleus (Fig. 1**A**). The stromal cytoplasmic staining of ADAMTS1 was mostly diffuse, compared to distinct nuclear staining of the epithelia. In case of benign tumours, only epithelial cells stained positive for ADAMTS1. This staining was noticeable compared to weak discreet diffuse stromal staining of ADAMTS1 evident in benign tumours. Expression of ADAMTS1 was significantly enhanced in high-grade/stage ovarian tumours compared to benign tumours (Fig. 1**A**).

Even though high expression of ADAMTS1 was noted in the epithelial compartments of tumour cells, no significant expression difference was observed in the epithelial compartment between stages/grades of tumours (Fig. 1**B**). The expression of ADAMTS1 was lower in stromal compartments, but there was a significant increase in expression with increasing stage and grade of tumours (Fig. 1**B**). This contributed to overall significant changes in the expression of ADAMTS1 in high grade/stages compared to benign tumours (Fig. 1**A** and B). This was consistent with significantly higher ADAMTS1 mRNA expression in epithelial components compared with mesenchymal components of ascites-derived tumour cells, separated as previously described. [37] (Fig. 1**C**).

In addition to above, we also noted higher expression of ADAMTS1 in an independent cohort of metastatic (omentum) serous ovarian tumours compared to primary HGSOC (Fig. 1**D**). Consistent with our above findings, higher ADAMTS1 expression was noted in epithelial tumour cells and stromal cells of metastatic versus primary tumours (Fig. 1**D**). In that case, an increasing trend of ADAMTS1 staining was noted in epithelial compared to stromal staining in both primary and metastatic tumours (Fig. 1**D**).

### In silico expression of ADAMTS1 and its prognostic potential in OC

**Fig. 2:**
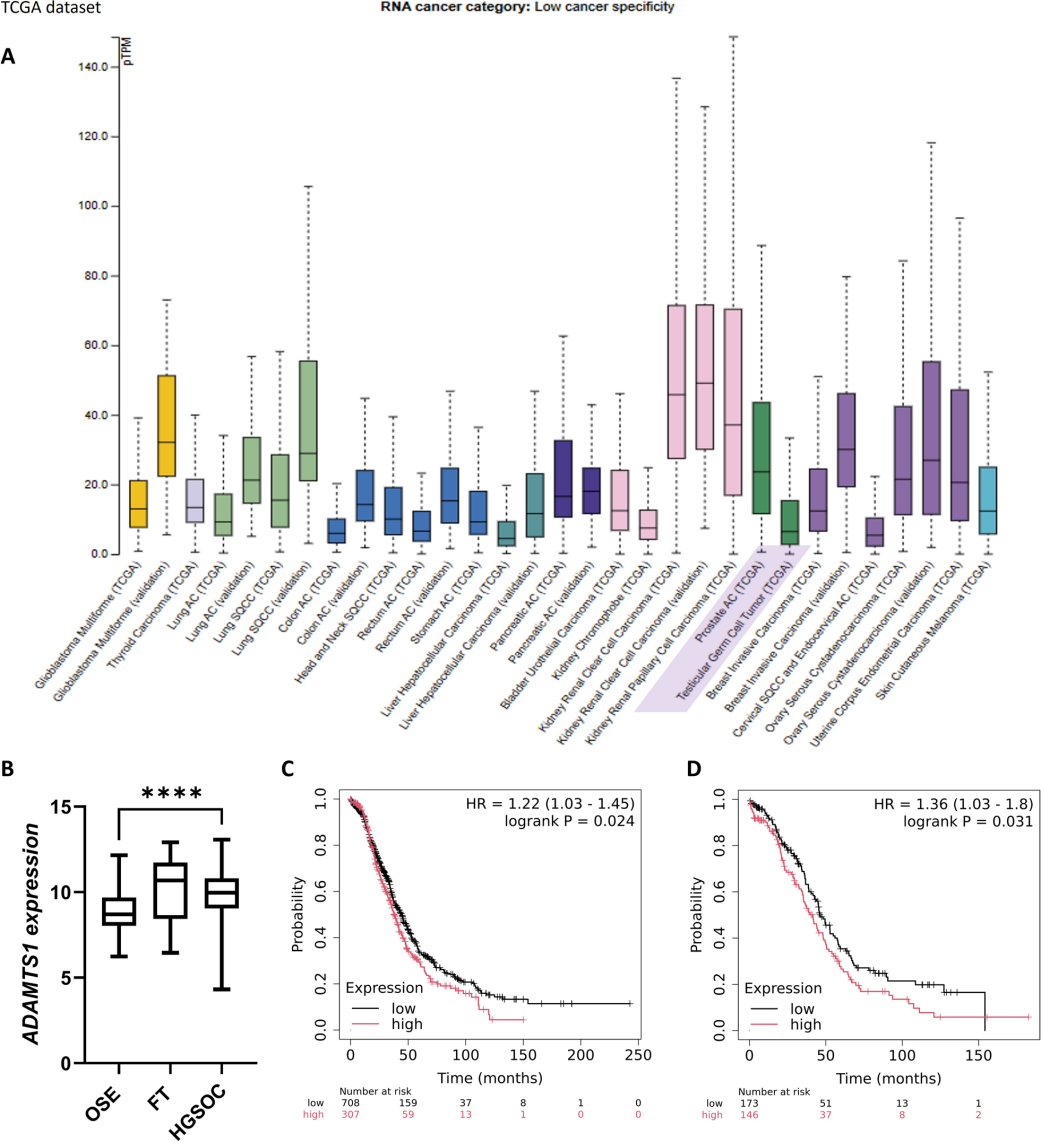
ADAMTS1 expression in public domain database. **A** ADAMTS1 expression in ovarian tumours compared to other tumours in TCGA dataset. Image acquired from the Human Protein Atlas (as described in the Materials and methods section) (https://www.proteinatlas.org/ENSG00000154734-ADAMTS1/cancer). **B** ADAMTS1 mRNA expression in OSE, FT and HGSOC based on GENT2 analysis (http://gent2.appex.kr/gent2/). Data are median with min and max values. P < 0.0001 (Kruskal Wallis test with Dunn’s multiple comparison test). **C** Kaplan–Meier survival curves for OC grade 3 patients was derived from mRNA-ChIP-seq database. This was used to differentiate between high ADAMTS1 expressing tumours (samples > 2297, n = 307) versus low ADAMTS1 expressing tumours (samples < 2297, n = 708). **D** This analysis used the mRNA-seq Pan Cancer database selected for grade 3 ovarian tumours, only had 319 samples. The expression probed used in the analysis had a range of 85-16,818 units, and the auto selection cutoff value used in analysis was 2098. This was used to determine high ADAMTS1 expressing tumours (samples > 2098, n = 146) versus low ADAMTS1 expressing tumours (samples < 2098, n = 173)

To determine the clinical significance of ADAMTS1 in OC patients, we initially compared the expression level of ADAMTS1 in OC tumours with other tumours across TCGA tumour database (Fig. 2**A**). The expression of ADAMTS1 in OC tumours was within the tumour groups with high expression of ADAMTS1 (Fig. 2**A**). Next, using the GENT2 database as described previously [40] we analysed ADAMTS1 expression in normal tissues, ovarian surface epithelium (OSE, n = 66), Fallopian Tube (FT, n = 40) and HGSOC (n = 806) tissues (Fig. 2**B**). The expression of ADAMTS1 was significantly high in HGSOC compared to OSE (Fig. 2**B**).

As the sample number of in-house samples was low, multivariate analysis was not possible. However, Kaplan–Meier plot derived from RNA-ChIP and Pan-cancer RNA-seq data bases using grade 3 OC tumours demonstrated that OC patients with ADAMTS1^high^ expression had reduced overall survival (OS) than patients with ADAMTS1^low^ expression (Fig. 2**C** and **D**).

### Opal staining of high-grade and benign serous ovarian tumours

**Fig. 3:**
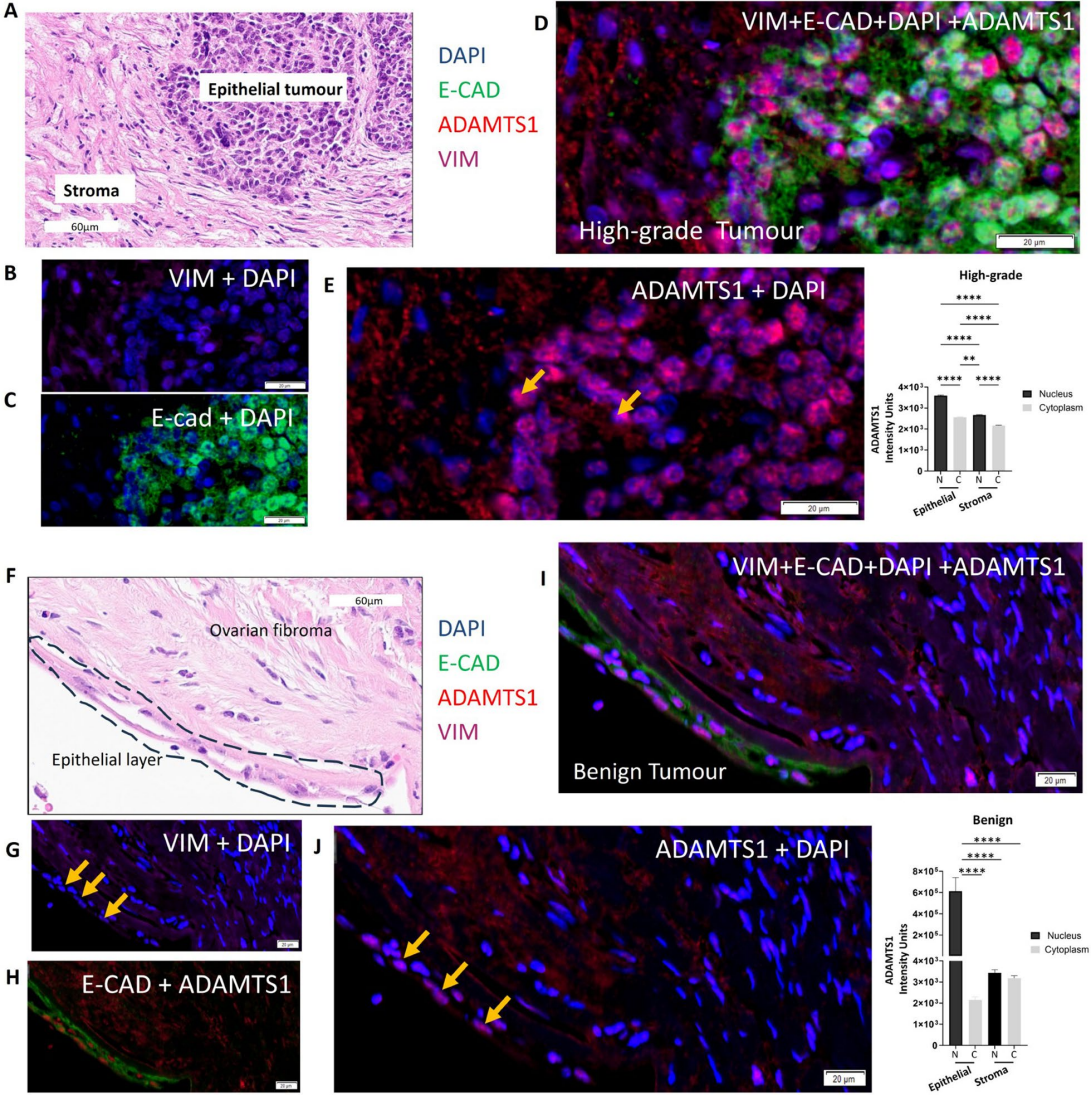
The Opal multiplexed immunohistochemical staining.** A, F** H & E image of **(A)** high-grade serous and **(F)** benign ovarian tumours used for OPAL multiplexed staining. Scale bar = 60 µm. The simultaneous staining of DAPI (nuclear, blue) and mesenchymal specific vimentin (VIM, magenta) **(B, E)**, epithelial specific E-cadherin (E-cad, green) **(C, D, H and I)**, and ADAMTS1 (red) (**D, E, I, J**) are shown. Composite images were produced according to the spectral wavelength of each fluorescent probe. Scale bar = 20 µm. **E, J** Epithelial and stromal cell detection and IF intensity measurement of nuclear and cytoplasmic ADAMTS1 in high-grade and benign tumours was performed by using QuPath (version 0.5.1). Error bars are presented as mean ± SEM. Significance is indicated by ** p < 0.01 or ****p < 0.0001, Mann–Whitney test

To visualize and analyse the distribution of ADAMTS1 expression within the nuclear and cytoplasmic compartments of benign and high-grade ovarian tumours, and to obtain a more comprehensive understanding of the localisation of ADAMTS1 expression in the tumour microenvironment we performed a multiplexed immunofluorescence staining using Opal Multiplex IHC kit on a small high-grade and benign tumour sets (n = 3). The expression intensities of ADAMTS1 (red), aligning with epithelial E-cadherin (green), mesenchymal vimentin (magenta) or DAPI nuclear (blue) in whole high-grade tumour was evaluated (Fig. 3**A–E**). Figure 3**A **represents the H & E image of a representative high-grade tumour and Opal staining is presented in Fig. 3**B–E**. Each marker showed positive intensities in the expected cellular compartment of each tumour sample. In high-grade tumours, ADAMTS1 expression was noted primarily in the nucleus of epithelial cells, more evident at the edge of tumour-stroma, while similar staining was also noted in other parts of epithelial compartments (Fig. 3**C–E**). Stromal staining of ADAMTS1 in high-grade tumours was mostly restricted to cytoplasm of tumour cells, even though some discreet nuclear staining was also visible (Fig. 3**B, D, E**). Consistent with that, ADAMTS1 was also localised in the nucleus of the epithelial lining of the surface epithelium of benign tumours (Fig. 3**I, J**). Nuclear and cytoplasmic localisation of ADAMTS1 was noted in the stroma of benign tumours (Fig. 3**I, J)**. The H & E image of a benign tumour is presented in Fig. 3**F.**

Quantification of the intensity of staining in these samples showed a significant increase in the nuclear staining in the epithelial cells compared to the stromal cells of high-grade tumours using Q-path analysis (Fig. 3**E**). Nuclear staining was significantly higher than cytoplasmic staining in both epithelial and stroma cells in high-grade tumours (Fig. 3**E**). Similarly, in benign tumours, nuclear staining in both epithelial and stromal cells was significantly higher than the cytoplasmic staining in both components (Fig. 3**J**). However, while nuclear staining in benign epithelial cells was significantly higher than cytoplasmic staining no difference between nuclear and cytoplasmic staining was observed in the stroma of benign tumours (Fig. 3**J**).

### Expression of ADAMTS1 in OC cell lines and its siRNA-mediated knocked down expression

**Fig. 4:**
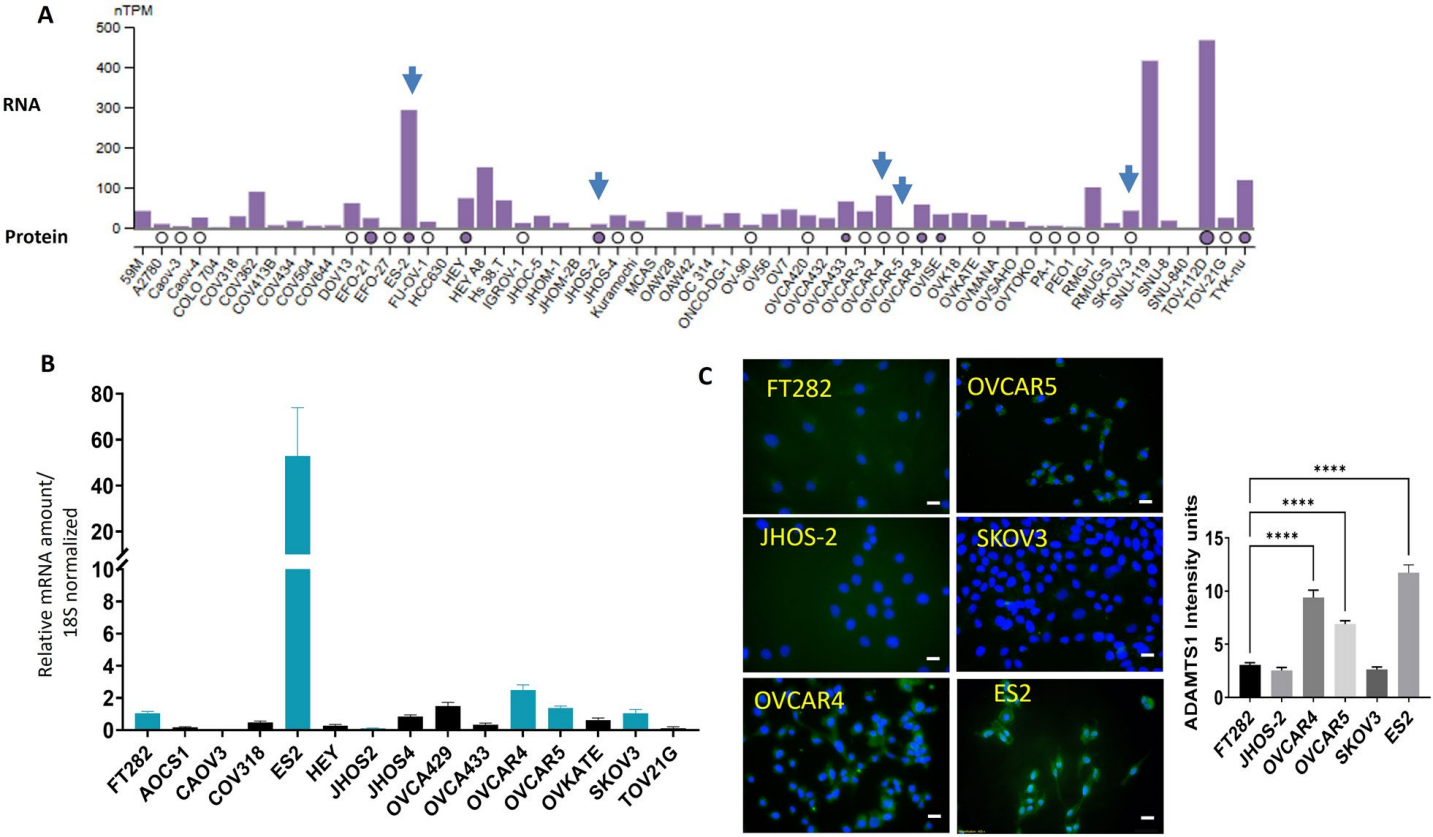
Expression of ADAMTS1 in OC cell lines. **A** mRNA and protein expression of ADAMTS1 in 59 ovarian cancer cell lines. Data derived from https://www.proteinatlas.org/ENSG00000154734-ADAMTS1/cell+line#ovarian_cancer. The RNA expression data is represented as normalized transcript per million (nTPM) values of cancer cell lines. While the protein expression based on MS proteomic dataset from the Pan-Cancer Atlas project is represented by a circle for each cell line (located under the graph). The size of the circle corresponds to the Normalized Relative Protein Expression (nRPX) value for the cell line, and the colour represents the tissue type to which the cell line is related. A white circle means that the protein was not detected by MS in the cell line, while no circle means that MS data was not available for the cell line. Blue arrows indicate cell lines selected in **(C)**. **B** ADAMTS1 mRNA expression in ovarian cancer cell lines derived from qRT-PCR as described in Methods. Error bars are presented as mean ± of SEM. **C** Protein expression of ADAMTS1 shown by immunofluorescence in FT282, JHOS-2, OVCAR4, OVCAR5, SKOV3 and ES2, cell lines as described in Methods. ADAMTS1 staining was visualized using fluorescent-labelled anti-rabbit Alexa 488 (green) secondary antibody and nuclei were detected by DAPI (blue) staining. Images are representation of merged DAPI (blue) and ADAMTS1 (green) staining on individual cell line done in three passages in triplicate. The intensity of fluorescence was obtained using FIJI software. 20X magnification; scale bar (in yellow) 20 μm. Error bars are presented as mean ± SEM. Significance is indicated by *** p < 0.001 where FT282 cell line is used as a control

We investigated the expression of ADAMTS1 in a range of OC cell lines in public domain dataset (www.proteinatlas.org). Variable level of expression of ADAMTS1 was noted in a range of OC cell lines (Fig. 4**A**). This was confirmed by in house evaluation of mRNA expression of ADAMTS1 in a range of OC cell lines (Fig. 4**B**). Among the cell lines ES2, had a considerably high expression of ADAMTS1, which was consistent with the public domain data set, while OVCAR4 and OVCAR5 had lower expression (Figs. 4**B**). Protein levels of ADAMTS1 were evaluated by IF (Fig. 4**C**). Cytoplasmic localization of ADAMTS1 was noted in all six cell lines analysed (Fig. 4**C**). However, some nuclear staining was evident in ES2 and OVCAR4 cell lines (Supplementary Fig. 2**A**).

**Fig. 5:**
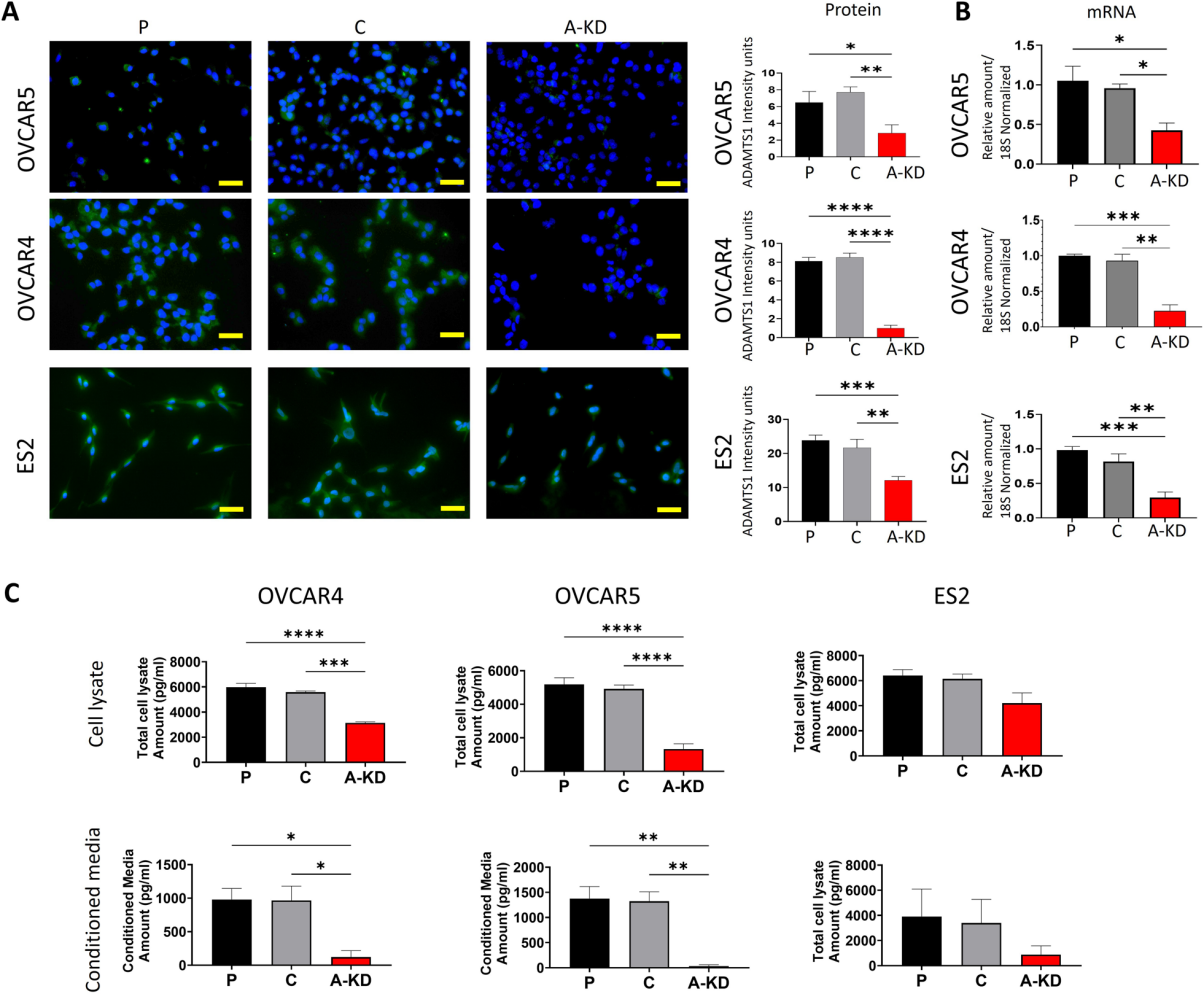
Knockdown of ADAMTS1 expression in OC cell lines by siRNA. Reduction of ADAMTS1 expression by siRNA in OVCAR4, OVCAR5 and ES2 cell lines. ADAMTS1 expression was knocked down by pooled siRNA transfection as described in the Methods. **A** The expression of ADAMTS1 at the protein level was deduced by immunofluorescence using rabbit polyclonal antibody as described in Methods. Staining was visualized using fluorescent-labelled anti-rabbit Alexa 488 (green) secondary antibody and nuclei were detected by DAPI (blue) staining. Images are representation of merged DAPI (blue) and ADAMTS1 (green) staining on individual cell line done in three passages in triplicate. The intensity of fluorescence was obtained using FIJI software. 20X magnification; scale bar (in yellow) 20 μm. P indicates parental cell line treated with transfection reagent, C are cells transfected with scrambled siRNA and A-KD are ADAMTS1 siRNA knock down cells. **B** The mRNA expression of ADAMTS1 in ADAMTS1 knock down cells was assessed by qRT-PCR. Error bars are presented as mean ± SEM. Significance is indicated by * p < 0.05, **p < 0.01, ***p < 0.001 and **** p < 0.0001, one-way ANOVA. **C** 48 h post transfection the protein expression of ADAMTS1 in 50 µg of cell lysate and conditioned media was evaluated by ELISA. The experiment was repeated three times in triplicate. Graphs represent mean of total protein ± SEM. Significance is indicated by * p < 0.05, **p < 0.01, and **** p < 0.0001, one-way ANOVA

To assess the functional role of ADAMTS1 in ovarian tumours, the expression of ADAMTS1 was knocked down (A-KD) in OC cell lines OVCAR4, OVCAR5 and ES2, by using three unique 27mer siRNA duplexes individually, or a pooled siRNA duplex (Supplementary Figs. 2**B** and **C**). A non-targeting universal siRNA was used as a control (C) in these experiments. ADAMTS1 mRNA and intracellular (cell lysate) and secreted protein (conditioned media) expression was reduced by 60–90% in these cell lines (A-KD) compared to control (C) and Parental (P) cell lines using single or pooled ADAMTS1 siRNA (Fig. 5**A**–C).

The knock down of ADAMTS1 (A-KD) in OC cell lines had no significant effect on the mRNA expression of natural inhibitors of ADAMS/ADAMTS (TIMP-1, −2, −3 and RECK) (Supplementary Fig. 3**A**) and or other associated ADAMTS or ADAMs which share homology sequence with ADAMTS1 (ADAMTS4, ADAMTS5, ADAMTS9, ADAMTS16, ADAM17, ADAM10 and ADAM12) (Supplementary Fig. 3**B**, **C**).

### Reduction in ADAMTS1 expression had no effect on cell proliferation but enhanced migration and reduced adhesion of OC cell lines

**Fig. 6:**
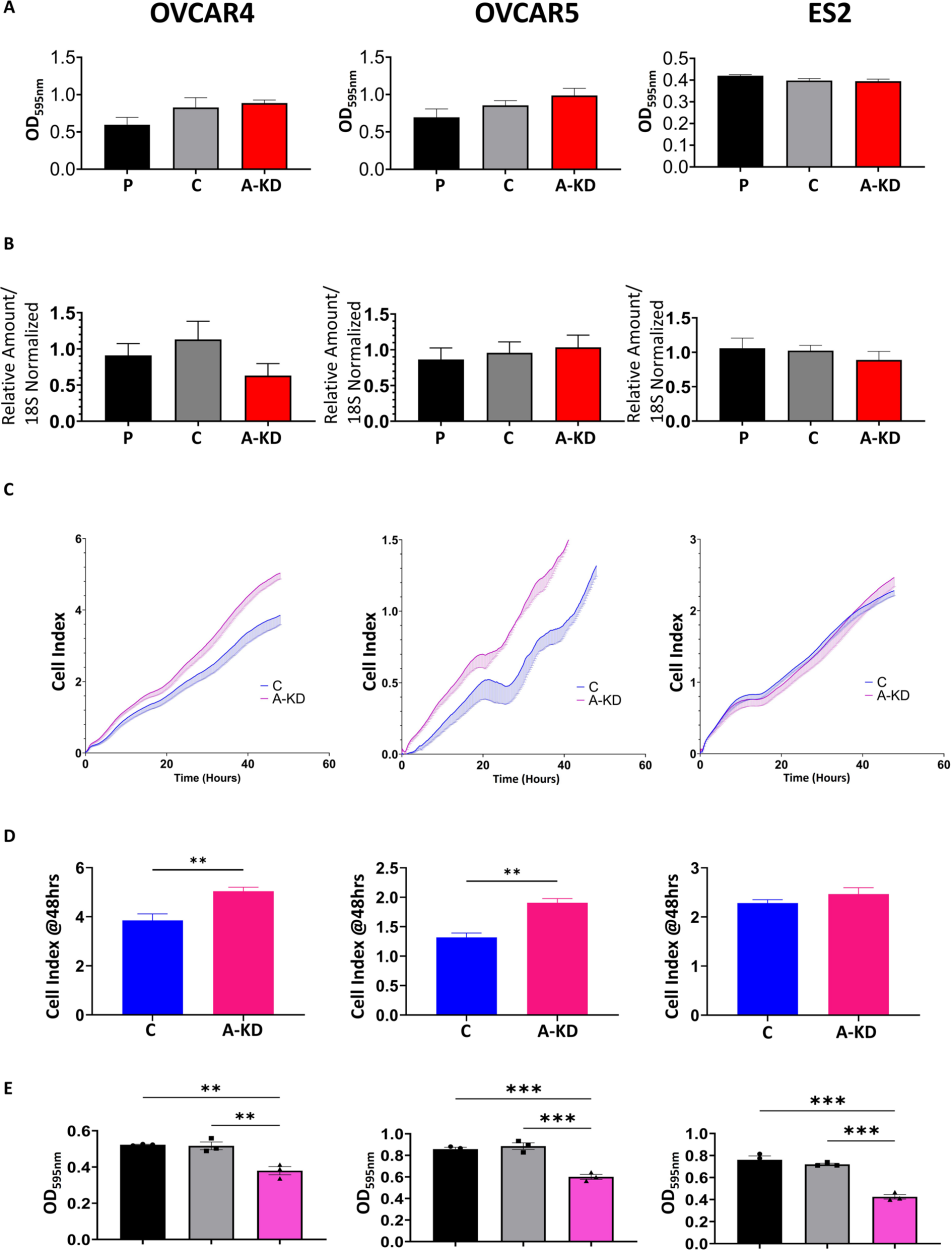
Effect ADAMTS1 knock down on proliferation, migration and adhesion of OC cell lines. Proliferation was assessed by **A** MTT assay and **B** Ki67 mRNA expression using qRT-PCR, while **C** migration was assessed by xCELLigence real time cell analysis. P indicates parental cell line treated with transfection reagent, C are cells transfected with scrambled siRNA and A-KD is a representative of a pool of all three ADAMTS1 siRNAs knock down cells. The experiment was repeated three times in triplicate. Graphs represent mean of total ± of SEM. In xCELLigence real-time cell analysis, the pink line represents A-KD while the blue line represents C scrambled siRNA transfected cells. **D** Significance in migration between the C and A-KD was assessed by plotting 48 h time point in a bar graph and assessed by unpaired t-tests, **p < 0.01. Graphs represent total mean ± SEM. **E** Adhesion was assessed 48 h post transfection of ADAMTS1 siRNA by adhesion assay as described in the Methods by plating cells in wells coated with Poly-L-Lysine, then staining with crystal violet and reading the results in a plate reader at OD_595_ nm. The experiment was repeated three times in triplicate. Graphs represent mean of total ± SEM. Significance is indicated by **p < 0.01, and *** p < 0.001, one-way ANOVA

The proliferation of OVCAR4, OVCAR5 and ES2 cell lines after siRNA knock down of ADAMTS1 (A-KD) and the relevant C and P cell lines was analysed 48 h after siRNA and control vector transfection using the MTT assay and mRNA expression of Ki67 by qRT-PCR. Both assays revealed no change in cellular proliferation in all A-KD cells compared to the matched C and P cells (Fig. 6**A** and **B**).

We next evaluated the migratory capacities of these cells by xCELLigence Real-time Cell Analysis. This revealed a significant enhancement of migratory abilities in A-KD OVCAR4 and OVCAR5 cell lines compared to the respective control cells (Fig. 6**C** and **D**). In contrast, ES2 cell line showed no change in migration in A-KD cells compared to the relevant control cells (Fig. 6**C **and **D**).

Coordinated attachment and detachment of cells to ECM is requisite for cellular proliferation and migration [49]. Hence, next we looked at the adhesion profile of cells in which ADAMTS1 was knocked down. Knocking down ADAMTS1 by siRNA (A-KD) significantly decreased adhesion in OVCAR4, OVCAR5 and ES2 cell lines (Fig. 6**E**).

### Reduction in ADAMTS1 expression results in ECM remodelling

ADAMTS1 has a known role in ECM remodelling which can facilitate tumour progression and metastasis [11]. ECM remodelling is a dynamic process in which MMPs play an essential role by affecting cell migration and adhesion [50]. In addition, cancer cells interact with the ECM through various integrins and growth factor receptors, that initiate intracellular signalling pathways which also regulate cell adhesion and migration [51].

**Fig. 7:**
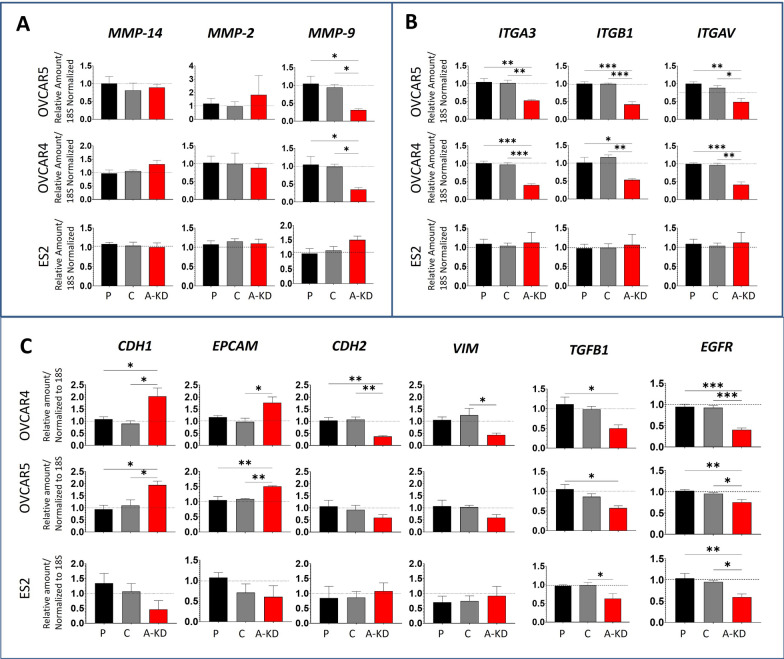
The effect of ADAMTS1 knock down on ECM genes. After 48 h post transfection of ADAMTS1 siRNA the mRNA expression on **A** MMPs, **B** integrins **C** and EMT associated genes: CDH1(E-Cad), EPCAM, CDH2 (N-Cad), VIM, TGFβ and EGFR in OVCAR4, OVCAR5 and ES2 cell lines was deduced by qRT-PCR. The experiment was repeated three times in triplicate. Graphs represent mean of total ± SEM. Significance is indicated by *p < 0.05, **p < 0.01, and *** p < 0.001, one-way ANOVA

Taking into consideration the ECM remodelling action of ADAMTS1, we assessed the expression of MMPs [MMP-2, MT1-MMP (MMP-14)] known to play an active role in ECM remodelling. We show that knock down of ADAMTS1 resulted in consistent significant decrease in the mRNA expression of MMP-9 in OVCAR4 and OVCAR5 cells, but no change was observed in ES2 cells (Fig. 7**A**). ADAMTS1 knock down had no effect on the expression of MMP-2 and MTI-MMP (MMP-14) on any of the cell lines tested (Fig. 7**A**).

Consistent with knockdown of MMP-9 in A-KD OVCAR4 and OVCAR5 cell lines, the mRNA expression of integrin α3 (ITGα3), αv (ITGαV) and β1 (ITGβ1) was also significantly reduced in A-KD OVCAR4 and OVCAR5 cell lines (Fig. 7**B**). However, no such change in integrin expression was noted in ES2 cell line (Fig. 7**B**).

### Reduction in ADAMTS1 expression contributes to the acquisition of altered cellular plasticity in mouldable tumour cells

To decipher the phenotypic changes accompanying ADAMTS1 knock down in OC cell lines we screened for the EMT markers in OC cell lines by qRT-PCR. Knock down of ADAMTS1 in OVCAR4 and OVCAR5 cell lines resulted in a multitude reduction of common mesenchymal markers at the mRNA level (Fig. 7**C**). Expression levels of EMT-specific genes such as N-cadherin (CDH2) and vimentin (VIM) were decreased in OVCAR4 A-KD cell line compared to relevant P and C cell lines (Fig. 7**C**). However, in OVCAR5 A-KD cells a downward trend was noted which did achieve significance level, while in ES2 cell line no change was noted (Fig. 7**C**). On the other hand, the expression of epithelial markers such as E-cadherin (CDH1) and EPCAM was significantly up regulated in both A-KD OVCAR4 and OVCAR5 cell lines but not in ES2 (Fig. 7**C**).

To further understand the role of cellular plasticity changes in OC cell lines in response to ADAMTS1 KD, we investigated the expression of known EMT-inducers, TGFβ [52] and EGFR [53]. ADAMTS1 KD in OVCAR4, OVCAR5 and ES2 cell lines led to significant decrease in the mRNA expression of these EMT-inducers (Fig. 7**C**).

In addition to that, we investigated the mRNA expression of EMT-associated transcription factors SLUG, SNAIL, TWIST, Frizzled-4 (FZD4), Frizzled-7 (FZD7) and the expression of scaffold protein (SHC1) in OVCAR4, OVCAR5 and ES2 cell lines before and after ADAMTS1 KD by siRNA (Supplementary Fig. 4). These transcription factors play major roles in essential aspects of tumour progression and metastasis, resistance to therapy and immune evasion [53, 54]. Suppression of these genes enhances E-cadherin and supresses mesenchymal markers such as VIM and N-cadherin [55]. In line with that, we saw in OVCAR4 cell line, significant downregulation of SHC1, TWIST1, FZD4 and FZD7 after ADAMTS1 KD compared to control cells (Supplementary Fig. 4). Both SLUG and SNAIL were also down regulated, but decreases were not significant. In OVCAR5 cell lines a downward trend of expression was noted in ADAMTS1 KD cells compared to vector control, but it did not reach significance. Furthermore, no defined changes in expression were noted after ADAMTS1 KD compared to control in ES2 cell line (Supplementary Fig. 4).

**Fig. 8:**
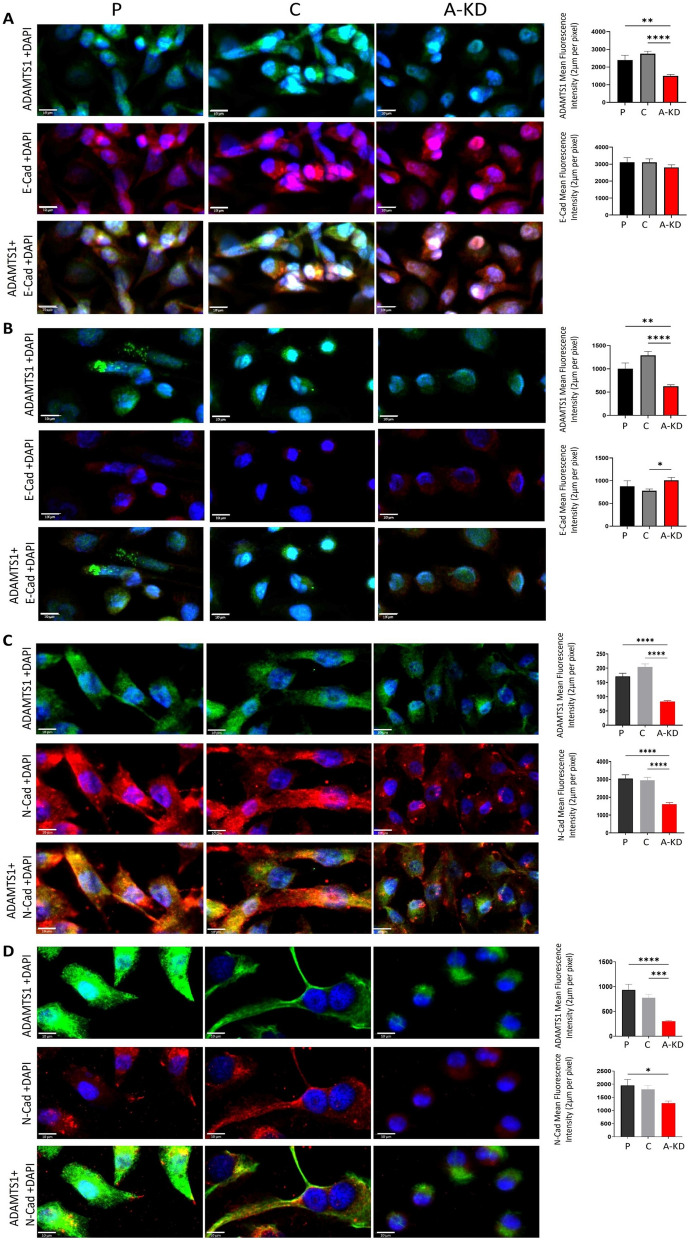
The effect of ADAMTS1 knock down on E-Cad and N-Cad protein expression. The protein expression of E-Cad and N-Cad in OVCAR4 **(A & C)** and OVCAR5 **(B & D)** cell lines was evaluated by immunofluorescence using ADAMTS1 (green) and N-Cad or E-Cad (red) antibodies as described in the Methods. DAPI stain (blue) was used for nuclear staining. Immunofluorescence quantification was done using QPath and the Mean Fluorescence Intensity (2 µm per pixel) was graphed ± SEM. Significance is indicated by *p < 0.05 and *** p < 0.001, one-way ANOVA

At the protein level, the expression of E-cadherin (E-Cad) (Fig. 8**A**, **B**) and N-cadherin (N-cad) (Fig. 8**C**, **D**) was assessed by IF in OVCAR4 (Fig. 8**A** and **C**) and OVCAR5 (Fig. 8**B** and **D**) cell lines. ADAMTS1 KD significantly increased the expression of E-cad in OVCAR5 cell line (Fig. 8**B**) compared to control cells but there was no change in the protein expression of E-Cad in OVCAR4 A-KD cell line compared to control cells (Fig. 8A). However, the expression of N-Cad was significantly attenuated in A-KD OVCAR4 and OVCAR5 cell lines, compared to control cells (Fig. 8**C**, **D**).

Interestingly, ADAMTS1 KD in both OVCAR4 and OVCAR5 cell lines resulted in distinct, smaller, polaroid, rounded cells compared to more elongated, bigger parental and control cell lines (Fig. 8**A**–**D**).

**Fig. 9:**
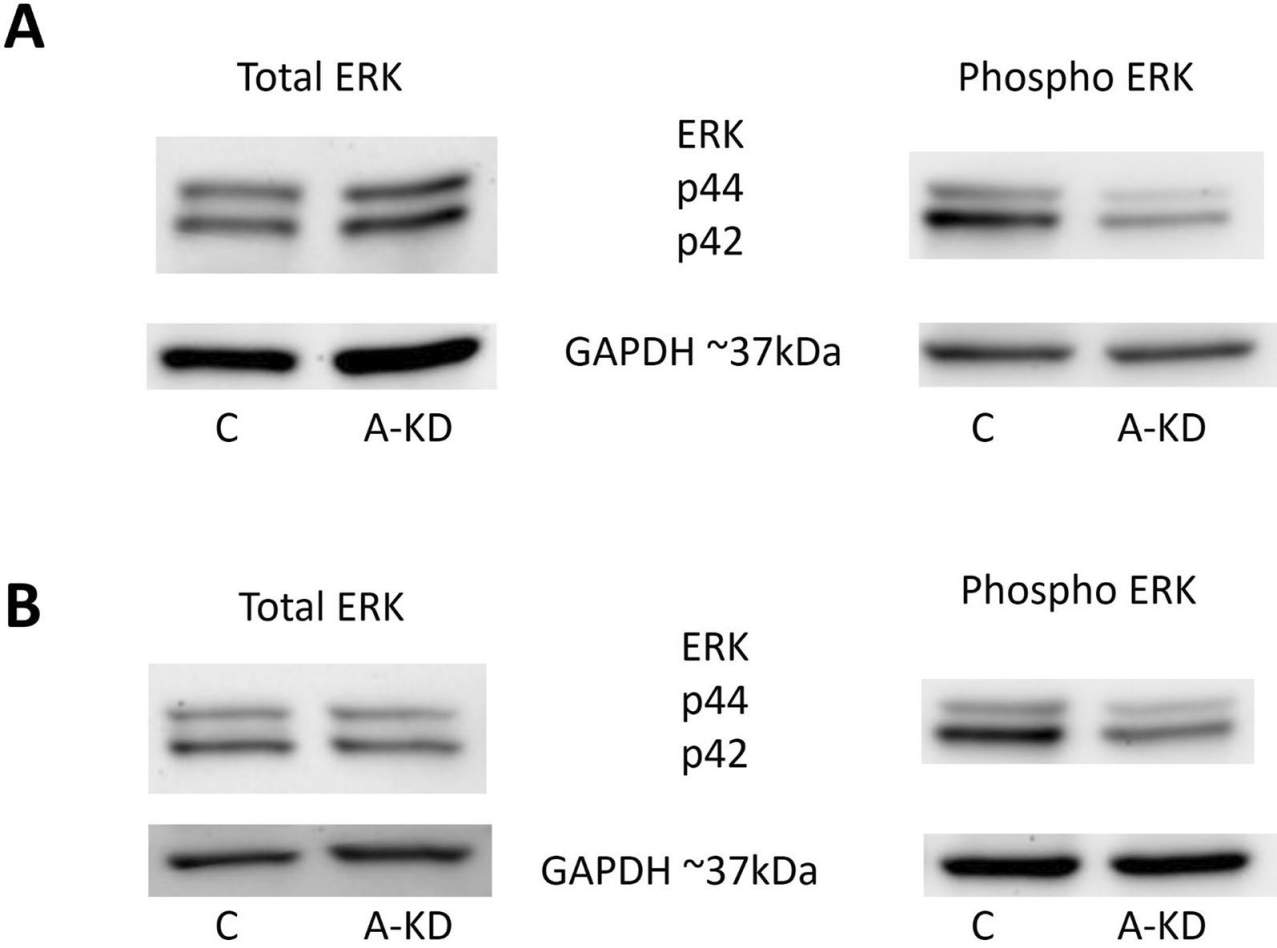
ADAMTS1 knock down decrease’s activation of Erk1/2 pathway. **A &B** After 48 h post transfection of ADAMTS1 siRNA, the activation of Erk1/2 pathway was investigated in **A** OVCAR4 and **B** OVCAR5 cell lines by Western blot

As activation of Erk is a critical signalling component of TGFβ1, EGFR, integrins-mediated signalling in different cancer models [54, 55], we investigated the Erk signalling pathway in both OVCAR4 and OVCAR5 cells in response to ADAMTS1 KD. Consistent with downregulated TGFβ1, EGFR, and integrin expression in response to A-KD in OVCAR4 and OVCAR5 cell lines, the expression of both phospho Erk1 (p44) and Erk2 (p42) was downregulated in A-KD compared to vector control (C) cells (Fig. 9). The expression of total Erk1 and Erk2, however, remained unchanged (Fig. 9). Supplementary Fig. 5 shows the raw blots from three experiments displaying expression of Erk and P-Erk.

### Reduction in ADAMTS1 expression contributes to low mRNA expression of Fibulin-1 (*FBLN1*), Syndecan-4 (*SDC4*) and L1 cell adhesion molecule (*L1CAM*)

**Fig. 10:**
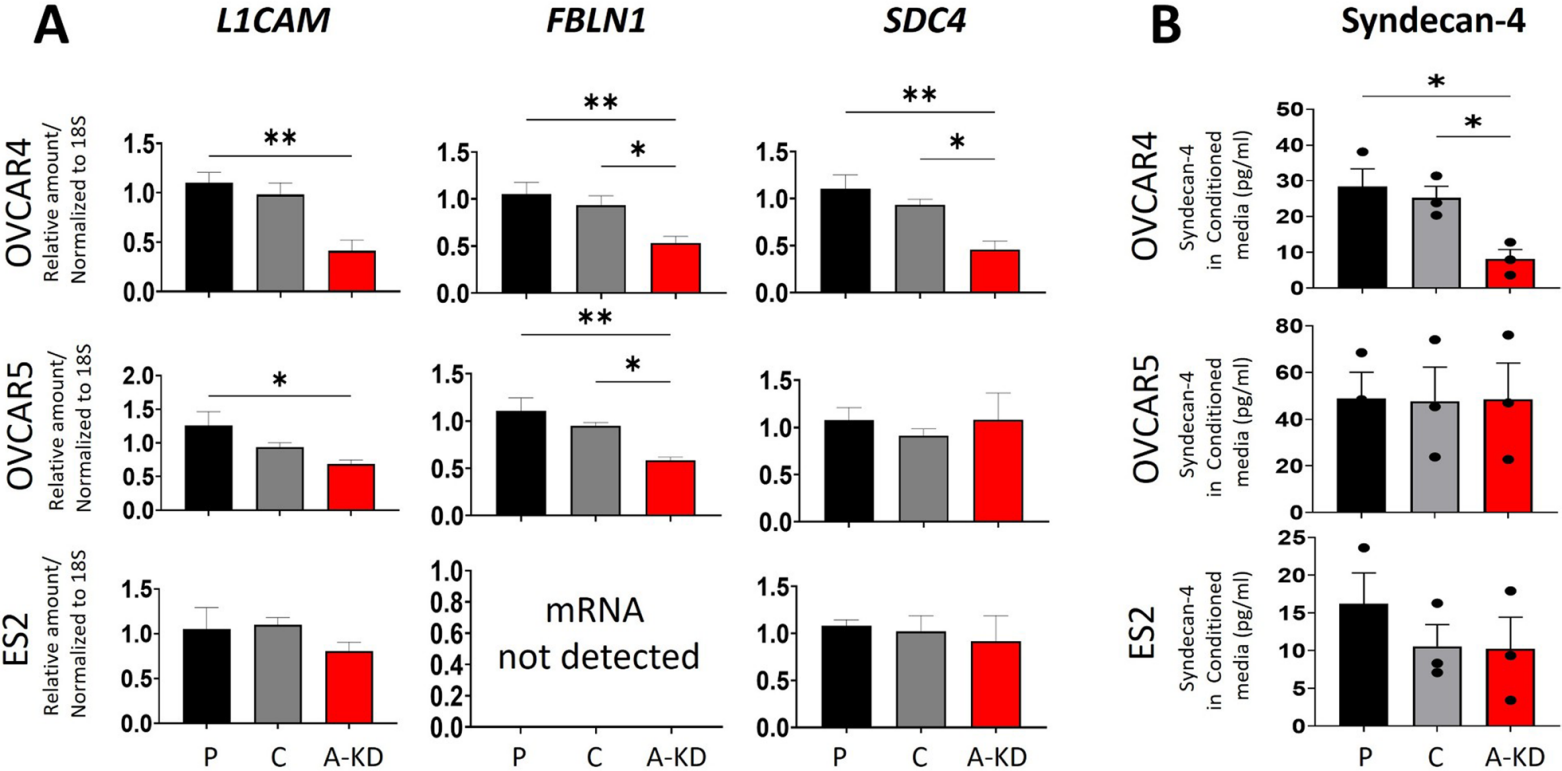
Effect of ADAMTS1 knock down on known ADAMTS1 regulated genes. **A** The mRNA expression of genes associated with ADAMTS1 regulation (L1CAM, FBLN1 and SDC4) was deduced by qRT-PCR after 48 h post transfection of ADAMTS1 siRNA in OVCAR4, OVCAR5 and ES2 cell lines as described in Methods. Graphs represent amount of mRNA relative to 18S ± SEM derived from three experiments done in triplicate. P indicates parental cell line treated with transfection reagent, C are cells transfected with scrambled siRNA and A-KD is a representative of a pool of all three ADAMTS1 siRNAs knock down cells. Significance is indicated by p < 0.05 and **p < 0.01, one-way ANOVA using Tukey’s multiple comparison test. **B** Secreted Syndecan-4 (SCD4) conditioned media levels were deduced by commercially available ELISA, as described in Methods. Graphs represent mean of total ± SEM, (n = 3). Significance is indicated by * p < 0.05 and ** p < 0.01, one-way ANOVA using Tukey’s multiple comparison test

To understand the role of ADAMTS1 KD in OC cell lines, we investigated the expression of ECM proteins previously shown to regulate ADAMTS1-mediated functions. Among those, the mRNA expression of L1 cell adhesion molecule (L1CAM), Fibulin (FBLN1) and proteoglycans syndecan-4 (SDC4) [18, 29, 56] were assessed. ADAMTS1 KD significantly downregulated the mRNA expression of these proteins in OVCAR4 cell line (Fig. 10**A**). However, in the OVCAR5 cell line only expression of FBLN1 and L1CAM was significantly downregulated (Fig. 10A). While in ES2, no such downregulated change was observed (Fig. 10**A**). The soluble ectodomain of SDC4, measured in conditioned media by syndecan-4 ELISA, also revealed significant downregulation in OVCAR4 A-KD cell line. However, this was not observed in either of OVCAR5 or ES2 A-KD cell lines compared to controls (Fig. 10**B**).

### Reduction in ADAMTS1 expression contributes to high mRNA and protein expression of VCAN in OC cell lines

ADAMTS1 is an active versicanase (degrading versican, VCAN), it cleaves VCAN to release a bioactive N-terminal fragment known as versikine [57]. All three (ADAMTS1, versican and versikine) play an active, independent roles in ovulation and embryogenesis by mediating ECM remodelling [17, 57]. We investigated the intracellular and extracellular expression of VCAN in response to A-KD in OC cell lines. VCAN gene consists of a N-terminal domain (G1), the central domain (G2) and the C-terminal domain (G3) (Supplementary Fig.  6**A**). There are four identified isoforms of VCAN (V0, V1, V2 and V3) (Supplementary Fig.  6**A**) of which only 2 of them (V1 and V0) are known to be involved in OC [58]. We show that the mRNA expression of V0, V1 and G1 domain is present in all three OC cell lines (Supplementary Fig. 6**B**).

**Fig. 11:**
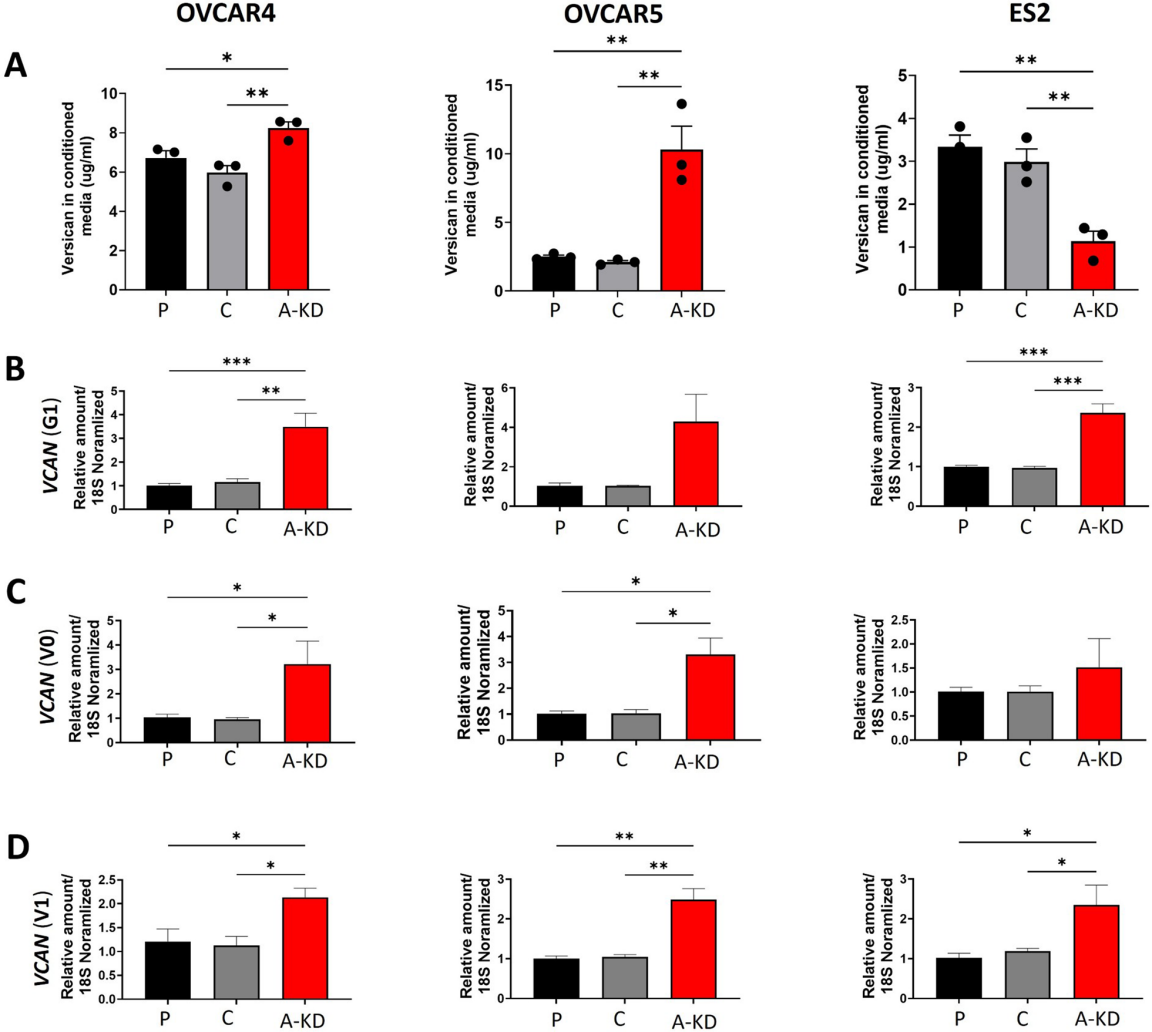
Effect of ADAMTS1 knock down on VCAN expression in OC lines. **A** After 48 h post transfection of ADAMTS1 siRNA, the level of VCAN in the conditioned medium of OVCAR4, OVCAR5 and ES2 cell lines was measured by ELISA as described in the Methods. The experiment was repeated three times in triplicate. Graphs represent mean of total ± SEM. Significance is indicated by *p < 0.05, **p < 0.01 by one-way ANOVA. The mRNA expression of **B** G1 domain and **C** V0 and **D** V1 isoforms was analysed at the mRNA level by qRT-PCR. P are the parental cell lines; C are cells transfected with scrambled siRNA and A-KD is a representative of a pool of all three ADAMTS1 siRNAs knock down cells. The experiment was repeated three times in triplicate. Graphs represent mean of total ± SEM. Significance is indicated by *p < 0.05, and **p < 0.01, one-way ANOVA

The extracellular protein expression of G1 domain of VCAN was enhanced in the cell-free medium (secreted, extracellular) of OVCAR4 and OVCAR5 following ADAMTS1 KD but was decreased significantly in the ES2 cell line (Fig. 11**A**). However, the mRNA level of VCAN (G1 domain) present in all VCAN isoforms was elevated in all three cell lines (Fig. 11**B**). We investigated the mRNA expression of V0 and V1 isoforms of VCAN in these cell lines, and as predicted V0 and V1 were expressed in all three cell lines (Fig. 11**D**). The expression of V1 was significantly elevated after ADAMTS1 KD in all three OVCAR4, OVCAR5 and ES2 cell lines (Fig. 11**D**). The mRNA expression of VCAN-V0 isoform was significantly elevated in OVCAR4 and OVCAR5 cell lines but not in ES2 cell line (Fig. 11**C**).

### Activation of Rho GTPase Cdc42 facilitates migration but has no effect on A-KD mediated cell adhesion and altered cellular plasticity in OC cell lines

**Fig. 12:**
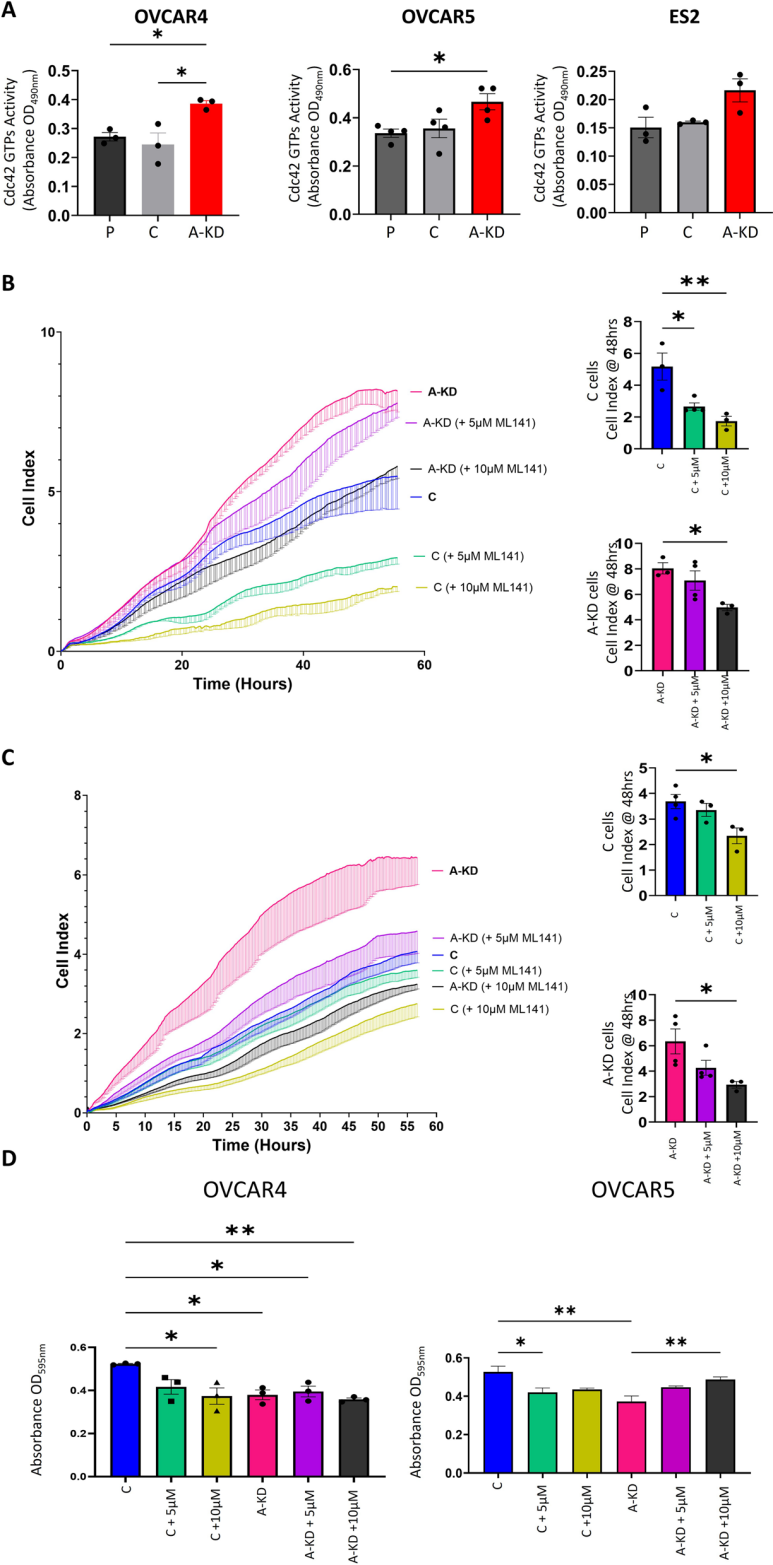
Effect of ADAMTS1 knock down on Cdc42 activity and inhibitory effect of ML141. **A** After 48 h post transfection of ADAMTS1 siRNA Cdc42 Activation Assay was conducted as described in Methods. The experiment was repeated three times in triplicate. Graphs represent mean of total ± SEM. Significance is indicated by *p < 0.05 by one-way ANOVA. Migration of **B** OVCAR4 and **C** OVCAR5 cell lines treated with 5 µM or 10 µM of the Cdc42 inhibitor (ML141) was assessed by xCELLigence real time cell analysis. **B** and **C** also demonstrate significance in migration at 48 h under different conditions. C are cells transfected with scrambled siRNA and A-KD is a representative of a pool of all three ADAMTS1 siRNAs knock down cells. The experiment was repeated three times in triplicate. Graphs represent mean of total ± of SEM. In xCELLigence real-time cell analysis, blue line represents C, green = C + 5 µM ML141, mustard colour = C + 10 µM ML141 the pink = A-KD, purple = A-KD + 5 µM ML141 and black = A-KD + 10 µM ML141. Significance (*p < 0.05) was calculated by plotting a graph of each treatment at 48 h and using on-way ANOVA. **D** Adhesion was assessed 48 h post transfection of ADAMTS1 siRNA and cells treated for 24 h with ML141 as described in the Methods. The experiment was repeated three times in triplicate. Graphs represent mean of total ± SEM. Significance is indicated by *p < 0.05, and **p < 0.01, one-way ANOVA

High expression and activation of the Rho family of small GTPase Cdc42 has been shown in different malignancies including OC [59, 60]. As Cdc42 is a master regulator in controlling cell motility and polarity we investigated the activity of Cdc42 in control and ADAMTS1 knocked down OC cell lines. The activity of Cdc42 was significantly elevated in response to A-KD in OVCAR4 and OVCAR5 cell lines but not in ES2 cell line compared to their control counterparts P and C (Fig. 12**A**). This activation of Cdc42 was inhibited by a commercially available Cdc42 specific inhibitor ML141 (Supplementary Fig. 7). Preliminary data indicated that 5 to 10 µM of ML141 inhibited the A-KD mediated activation of Cdc42 in OVCAR4 cell line to control levels (Supplementary Fig. 7).

To test if the inhibition of activated Cdc42 in response to ADAMTS1 KD has any effect in ADAMTS1 KD-mediated enhanced migration, ML141 at 5 and 10 µM concentrations were used for migration assays. ML141 at 10 µM concentration reduced the migration efficiency of A-KD OVCAR4 and OVCAR5 cell lines to near control levels (Fig. 12**B **and **C**). ML141 also inhibited the migration of control cells (Fig. 12**B** and **C**).

Next, we checked the effect of ML141 on the adhesive abilities of control and ADAMTS1 knocked down OVCAR4 and OVCAR5 cell lines. ML141 at 5 and 10 µM concentrations did not reverse the ADAMTS1 KD adhesive effects OC cell lines (Fig. 12**D**).

We investigated whether ML141 treatment could reverse the acquisition of epithelial phenotype induced by A-KD in OVCAR4 and OVCAR5 cells. ML141 at 10 µM concentration itself enhanced the expression of CDH1 and EPCAM but had no significant effect in reversing the enhanced CDH1 or EPCAM mRNA expressions in A-KD OVCAR4 and OVCAR5 cell lines (Supplementary Figs. 8**A** and **B**). However, ML141 reduced CDH2 and VIM expression in OVCAR4-A-KD cell line. In OVCAR5 cell line only VIM but not CDH2 expression were reduced (Supplementary Fig. 8**B**). Consistent with that, ML141 had no effect on the A-KD induced downregulated mRNA expression of EMT inducers (TGFβ and EGFR) (Supplementary Fig.  8**C**).

### Versican siRNA (V-KD) reverses the reduced expression of ADAMTS1 induced by ADAMTS1 siRNA (A-KD) in OC cell lines

**Fig. 13:**
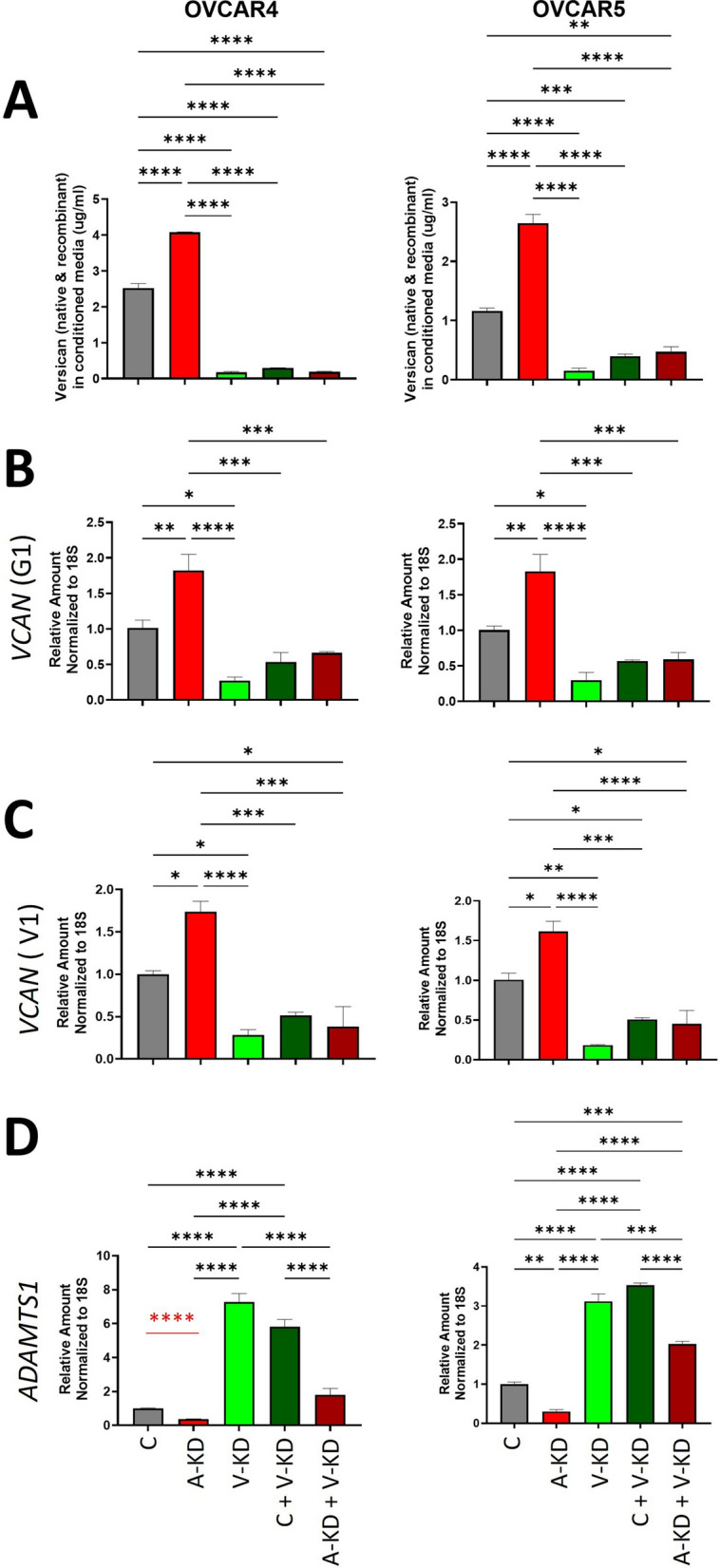
Effect of VCAN knock down in ADAMTS1 knocked down OC cell lines. **A** After 48 h post transfection of VCAN and/or ADAMTS1 siRNA the levels of VCAN in conditioned medium of OVCAR4 and OVCAR5 cell lines were measured by ELISA as described in the Methods. The experiment was repeated three times in triplicate. Graphs represent mean of total ± SEM. Significance is indicated by *p < 0.05, **p < 0.01, ***p < 0.001, ****p < 0.0001 by one-way ANOVA. The mRNA expression of **B** G1 domain picks up all isoforms of VCAN and **C** V1 isoform of VCAN and **D** ADAMTS1 was analysed in OVCAR4 and OVCAR5 cell lines treated with VCAN and ADAMTS1 siRNAs. Grey bar (**C**) indicates cell line transfected with scrambled siRNA; red bar (A-KD) is a representative of a pool of all three ADAMTS1 siRNAs knock down cells; light green bar (V-KD) is cell line transfected with VCAN siRNA; dark red bar (C + V-KD) is cell line transfected with C siRNA + VCAN siRNA; and dark green bar (A-KD + V-KD) is a cell line transfected with ADAMTS1 and VCAN siRNAs as described in the method section. The experiment was repeated three times in triplicate. Graphs represent mean of total ± SEM. Significance is indicated by *p < 0.05, **p < 0.01, ***p < 0.001, ****p < 0.0001 by one-way. Red asterisks indicate pairs analysed by unpaired t-test

In Fig. 11 we have shown that the intra- and extracellular expression of VCAN increases in response to A-KD in OVCAR4 and OVCAR5 cell lines. To investigate if the functional and other genomic changes induced by ADAMTS1 knockdown can be reversed by a parallel knockdown of VCAN gene in OC cell lines, a commercially available predesigned 21mer VCAN duplex siRNA was transfected in both OVCAR4 and OVCAR5 cell lines. The VCAN siRNA (V-KD) targets a section of the exon 1 (G1 domain) of VCAN gene, hence it supresses all four isoforms of VCAN. A non-targeting universal siRNA used for ADAMTS1 knock down was used as a control (C) in these experiments. As shown in Fig. 13**A**, the protein expression of secreted G1 domain of VCAN was significantly upregulated in both A-KD OVCAR4 and OVCAR5 cell lines. However, the protein expression of G1 was significantly down regulated in V-KD, and double transfected C + V-KD and A-KD + V-KD cell lines (Fig. 13**A**). These results are consistent with mRNA expression of G1 and V1 isoforms of VCAN under similar experimental condition (Fig. 13**B** and **C**). Under this same condition the expression of ADAMTS1 was significantly high in V-KD and double transfected C + V-KD and to lesser extend A-KD + V-KD cells lines compared to C and A-KD transfected cell lines (Fig. 13**D**).

### Versican siRNA can reverse the effect of ADAMTS1 knockdown (A-KD) on adhesion and migration in OC cell lines

**Fig. 14:**
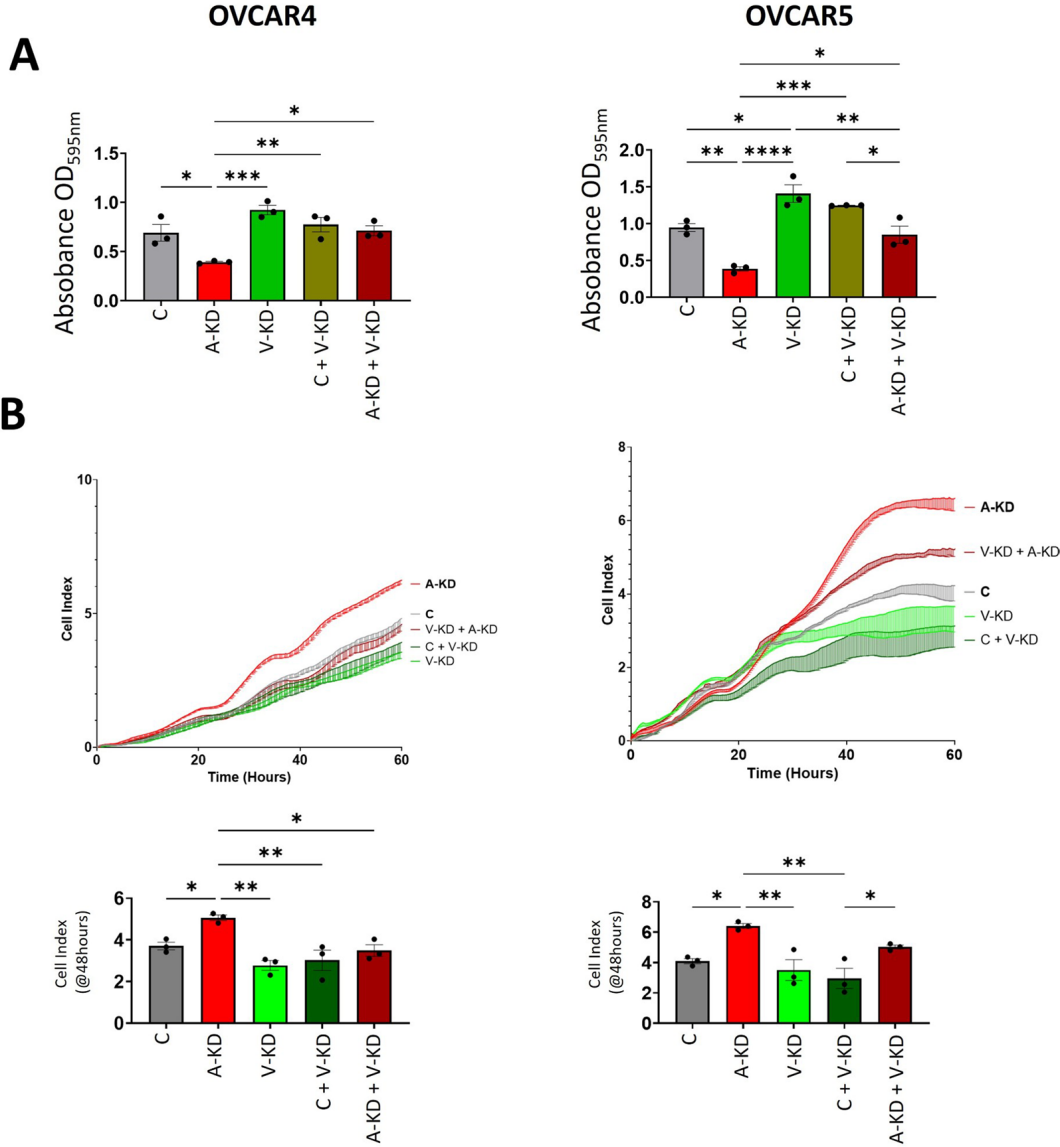
Effect of VCAN knockdown on the adhesion and migration of ADAMTS1 KD cells. **A** Adhesion and **B** migration in OVCAR4 and OVCAR5 cell lines treated 48 h with VCAN and ADAMTS1 siRNAs was measured as described in the Methods. **B** also demonstrates significance in migration at 48 h under different experimental condition. Grey bar (C) indicates cell line transfected with scrambled siRNA; red bar (A-KD) is a representative of a pool of all three ADAMTS1 siRNAs knock down cells; light green bar (V-KD) is cell line transfected with VCAN siRNA; dark red bar (C + V-KD) is cell line transfected with control siRNA + VCAN siRNA; and dark green bar (A-KD + V-KD) is a cell line transfected with ADAMTS1 siRNA and VCAN siRNA as described in the method section. The experiments were repeated three times in triplicate. Graphs represent mean of total ± SEM. Significance is indicated by *p < 0.05, **p < 0.01, ***p < 0.001, ****p < 0.0001 by one-way ANOVA

To test if the upregulation of VCAN in response to A-KD has any effect in A-KD-mediated suppression of adhesion and enhanced migration, assays were performed in OVCAR4 and OVCAR5 cell lines after double transfection with ADAMTS1 and VCAN (A-KD + V-KD) (Fig. 14). V-KD on its own had a significant effect on increasing adhesion in OVCAR4 and OVCAR5 cell lines compared to A-KD cells (Fig. 14**A**). Compared to A-KD the adhesion of cells increased significantly in all cell lines transfected with either V-KD on its own or used in a double transfection with either C or A-KD (Fig. 14**A**). On the other hand, the enhanced migration in A-KD cells compared to C was reversed in A-KD + V-KD cell lines bringing it to the level of C (Fig. 14**B)**. However, no conclusive changes could be observed in the A-KD induced epithelial plasticity with V-KD on A-KD cell lines (data not shown).

To see if ML141 has any effect on ADAMTS1 and VCAN expression in response to A-KD induction in OVCAR4 and OVCAR5 cell lines, mRNA expression of ADAMTS1 and VCAN was measured in cells treated with ML141 before induction of A-KD in ovarian OVCAR4 and OVCAR5 cell lines. No change in the mRNA expression of ADAMTS1 and VCAN was observed under these conditions (Supplementary Fig. 9). This suggests ML141 has GTPase specific effect and does not affect the mRNA expression of the target proteins.

## Discussion

ADAMTS1 is an ECM-associated protease which has shown to have a significant impact on the progression of different types of cancer [16, 28–30]. Different studies have presented overexpression or under expression of this protease in different tumour types compared to control-normal tissues, implying its pro- or anti-tumorigenic roles in different cancers [10, 11]. This discrepancy in the expression of ADAMTS1 with tumour progression in different tumour streams/models is not precisely known but it can be related to the expression of full length catalytically active ADAMTS1 known to promote metastasis, while the expression of catalytically inactive -NH2 and -COOH fragments can have an inhibitory effect on metastasis [4]. Considering that catalytically active status of ADAMTS1 has been reported in ovarian biology [62], enhanced expression of catalytically active ADAMTS1 in ovarian tumours under these conditions should supposedly promote pro-metastatic features of high-grade ovarian tumours.

To date there have been limited studies that examined the expression pattern of ADAMTS1 in primary benign and metastatic high-grade/stages of ovarian tumours and studied in detail the phenotypic and functional changes in OC cells in response to ADAMTS1 expression changes. Our current work provides novel insights into the expression pattern of ADAMTS1 in benign, different grades/stages and metastatic ovarian tumours and describes how decreasing the expression of ADAMTS1 in in vitro cultures in OC cell lines impacts cell migration, adhesion and cellular plasticity through different ADAMTS1-mediated pathways.

We describe for the first time the expression pattern of ADAMTS1 in relation to cellular localisation in different stages and grades of ovarian tumours compared to benign ovarian tumours. The fact that the expression of ADAMTS1 in ovarian tumours coincided mostly with the expression of CA125 in high-grade serous ovarian tumours, indicates that ADAMTS1 is mostly expressed by epithelial tumour cells. However, the enhanced trend of epithelial staining of ADAMTS1 expression with increasing grades/stages did not show significance with respect to benign tumours in the small cohort of samples used in the study. Contrary to that, less intense diffuse stromal staining of ADAMTS1 evident in malignant tumours were significantly high in different stages/grades compared to benign tumours, contributed to the overall significant higher expression in different stages/grades of tumours compared to benign tumours. These observations are consistent with significantly increased mRNA expression of ADAMTS1 in epithelial compared to mesenchymal component of ascites-derived tumour cells accrued from OC patients. We also report that ADAMTS1 protein expression is significantly higher in metastatic tumours derived from the omentum compared to primary serous ovarian tumours. Our observations are consistent with a previous study which used immunohistochemistry and demonstrated overall increased expression of ADAMTS1 in borderline and malignant tumours compared to benign ovarian tumours, classified based on World Health Organisation (WHO) tumour stage/grade grouping [24]. These coherent observations also conform with GENT2 mRNA data in which significantly enhanced expression of ADAMTS1 was noted in ovarian tumours compared to normal ovarian tissues. Further to that, by OPAL staining, for the first time we demonstrate intense, discreet nuclear expression of ADAMTS1 in epithelial tumour cells compared to diffuse cytoplasmic expression in stromal cells of both high-grade and benign tumours. In both benign and high-grade tumours nuclear epithelial staining was much higher than cytoplasmic nuclear or stromal staining. In that context, nuclear localization of a ADAMTS1 can be associated with enhanced transcription, replication and ribosome biogenesis [23], while cytoplasmic localization may relate to translation, metabolism, transportation and signal transduction [24]. Nuclear localization of ADAMTS1 mainly observed in the epithelial tumorigenic cells, may indicate that these cells are undergoing an increase in ADAMTS1 transcription, thus leading to increase in secretion of ADAMTS1 for maintenance of stromal functions [16]. On the other hand, cytoplasmic localization of ADAMTS1 in the stroma may relate to an elevated signalling in these cells to remodel the stroma requisite for the sustenance and promotion of pro-metastatic functions [5]. These observations align with recent studies which detected predominant nuclear localization of ADAMTS1 in epithelial mammary cells, while cells of mesenchymal origin had the protease localised in the cytoplasm [63, 64]. One of these studies further went to show that nuclear localisation of ADAMTS1 can be shifted to cytoplasmic expression by disrupting Golgi apparatus architecture and function by an inhibitor, Monensin, indicating that ADAMTS1 requires to be secreted and endocytosed to reach the nuclei [64]. This may suggest a potential nuclear matrix remodelling ability of ADAMTS1 like that shown previously for ECM remodelling [10]. In agreement with this, a recent study has supported the nuclear role of VCAN shown to participate in mitotic spindle organisation during cell division of vascular cells [65]. In that scenario, nuclear ADAMTS1 may be required to cleave VCAN as a prerequisite for its nuclear mitotic spindle organisation and function.

We show transient suppression of ADAMTS1 (80–90%) expression by siRNA in three different OC cell lines, OVCAR4, OVCAR5 and ES2. In these cell lines ADAMTS1 expression was observed in the nucleus and the cytoplasm and transient knockdown of ADAMTS1 did not show any effect on the localisation of this protease. Suppression of ADAMTS1 in vitro, had no impact on the proliferation of these cells but instead enhanced the migration and decreased adhesion of OC cell lines. Even though decreased adhesion was observed in all three OC A-KD compared to C cell lines (OVCAR4, OVCAR5, ES2), no enhanced effect on migration was observed in ES2 cell line. Contrary to that, CRISPR knock down of ADAMTS1 in ES2 cell line previously showed no effect on proliferation but significantly enhanced migration [31]. With the same token, treatment with conditioned medium enriched in ADAMTS1 diminished the migration of CRISPR ADAMTS1 knocked down ES2 cell line [31], suggesting that the level of intra and extracellular expression of ADAMTS1 impacts cancer cell migration. The migration of ES2 cell line in CRISPR knock down scenario was associated with enhanced Cdc42 GTPase activity which was reduced in the presence of ADAMTS1 enriched medium with consequent inhibition of migration [31]. This is consistent with our findings where we report increased activity of Cdc42 GTPase activity correlating with increased cellular migration in A-KD OVCAR4 and OVCAR5 cell lines compared to control cells and abolishment of that enhancement in migration in the presence of Cdc42 GTPase specific inhibitor ML141. However, this observation of enhanced Cdc42 GTPase activity with the knock down of ADAMTS1 expression was not seen in the ES2 cell line. These studies suggest that the expression level of ADAMTS1 may regulate Cdc42 GTPase dependant migratory phenotype of OC cells. In that context, it should be highlighted that ES2 has several-fold higher expression of ADAMTS1 compared to OVCAR5 and OVCAR4 cell lines, 80% transient knock down of ADAMTS1 by siRNA in these cells still leaves behind considerable level of residual intracellular ADAMTS1 expression, presence of which may hinder the enhanced migratory abilities of ES2 cells in response to ADAMTS1 knock down.

To further explore the mechanisms of A-KD-mediated increased migration and decreased adhesion, the mRNA expression of ADAMTS1 regulated specific ECM substrates such SDC4, FBLN1 and L1CAM [18, 26, 29], which previously have shown to regulate cellular migration and adhesion, was investigated in A-KD OC cell lines compared to control C cell lines. Significantly decreased mRNA expression of L1CAM, FBLN1 and SDC4 with concomitant low secretion of cleaved ectodomain of SCD4 was noted in OVCAR4 A-KD cell line. In OVCAR5 cell line, even though L1CAM and FBLN1 mRNA were downregulated, no such observation could be made in ES2 cell line. These observations are consistent with decreased mRNA expression of MMP-9, identified to cleave SCD4 and liable for its reduced cell surface expression [20]. Loss of MMP-9 mRNA expression occurred in concordance with the loss of α3, αv and β1 integrin subunit mRNA expression conforming altered ECM remodelling, which results in the loss of functional stabilization of integrins with leads to subsequent defects in adhesion stability and simultaneous enhanced motility of cells.

FBLN1 is an ECM protein and a cofactor of ADAMTS1 [26]. The over expression of this protein has been reported to inhibit migration in a variety of cell types by interacting with various ECM components and cell surface receptors, such as integrins and EGFR [66, 67]. Loss of FBLN1 expression in response to ADAMTS1 knock down maybe a factor contributing to enhanced migration in OVCAR4 and OVCAR5 cell lines. However, in OVCAR4 cell line loss of FBLN1 in conjunction with shedding of SCD4 which though decreased in A-KD cells may also contribute to increased migration and decreased adhesion as shown previously [20].

We further demonstrate that the loss of ADAMTS1 expression in OVCAR4 and OVCAR5 cell lines results in altered cell plasticity in the context of enhanced expression of epithelial specific CDH1 and EPCAM mRNA and decrease in the mRNA expression of mesenchymal CDH2 and VIM. This was consistent with the gain of CDH1 and loss of CDH2 protein expression, coherent with decreased mRNA expression of EGFR and TGFβ and down regulation of EMT-inducing transcription factors such as TWIST1, SLUG, SNAIL, FZD4, FZD7 and SHC1. Consistent with these changes, loss of downstream Erk1/2 signalling in A-KD OVCAR4 and OVCAR5 was observed compared to C cell lines. In that context, ADAMTS1^high^ has previously been shown to induce EMT through TGFβ in non-small lung cancer [28], and through increase in ADAMTS1-L1CAM-EGFR axis in oral squamous cell carcinoma models [29]. In addition, ADAMTS1-VCAN-EGFR axis has been shown to drive anoikis resistance and invasion in renal cell carcinoma [30]. Hence, it is not surprising that the loss of TGFβ, *EGFR* and the expression of EMT-inducing transcription factors in response to ADAMTS1^low^ (A-KD) coincides with the loss of mesenchymal CDH2 and VIM and gain of epithelial CDH1 and EPCAM. These data suggest that KD of ADAMTS1 in certain OC cell lines that harbour low initial expression of ADAMTS1 (as in the case of OVCAR4 and OVCAR5 cell lines), initiation of MET-like process may be noticeable which may not occur in cell lines expressing considerably high amount of ADAMTS1. Even though TGFβ and EGFR-mediated EMT-induced migration in cancer cells is well recognised, selective studies have shown epithelial cells, especially those from branched organs, such as lung, kidney, mammary gland, and even epithelial cancer cells to undertake migration as a part of their developmental program [68, 69]. In addition, in mammary epithelium there has been suggestions that self-inhibition of TGFβ signalling may contribute to collective epithelial cell migration [70].

The loss of ADAMTS1 expression in OC cell lines coincided with the gain of VCAN expression. This gain in VCAN expression modulates enhanced migration and attenuated adhesion as knock down of VCAN (V-KD) in A-KD OVCAR4 and OVCAR5 cell lines reverses these processes. This shows a direct link between ADAMTS1 and VCAN expression in our in vitro experimental model. Previous studies have shown significantly high expression of VCAN in ovarian tumours compared to normal ovaries and addition of recombinant VCAN to in vitro cultures accelerated cancer cell invasion [58]. Consistent with that, another study has shown that ectopic expression of V1 isoform of VCAN induces mesenchymal-epithelial transition (MET) in NIH3T3 fibroblasts and inhibition of endogenous VCAN expression abolished MET in metanephric mesenchyme [71]. Further to that, VCAN secreted by ovarian Fallopian Tube cells or exogenous addition of VCAN has been shown to enhance migration in both murine and human Fallopian Tube epithelial cells of the ovary [17]. These observations suggest that VCAN expression modulated by alteration in ADAMTS1 expression in vitro impacts OC cell functions such as migration and epithelial cell plasticity.

## Limitations of the study

As the tissue cohort used in this study was relatively small, the clinical analysis of the expression of ADAMTS1 in tumour grades and stages could not be correlated with clinical parameters such as OS and no multivariate analysis could be done. However, survival association with ADAMTS1 expression in high-grade tumours was deduced using two independent data sets which showed longer OS in ADAMTS1^low^ compared to ADAMTS1^high^ expressing tumour groups. In these analyses grade 3 patients were selected as they represent high-grade patients in our study.

The molecular pathways resulting in the alteration of cellular ADAMTS1 expression is complex and its true evaluation in terms of precise mechanism in OC biology is beyond the scope of current study. In this study, for the first time, we have explored the expression pattern of ADAMTS1 in relation to OC progression and tried to understand the functional alterations in OC cell lines in relation to its expression changes in the cells. In a seemingly paradoxical way, we report consistently high expression of ADAMTS1 in high grades/stages and metastatic ovarian tumours but knock-down expression of ADAMTS1 in vitro to facilitate cancer cell migration which coincided with reduced adhesion. This seemingly inconsistency in findings between the clinical observation (higher ADAMTS1 expression with increasing grades/stages of tumours) and in vitro experimental findings (enhanced migration after ADAMTS1 knockdown) is hard to explain. Nonetheless, consistent with our findings, ADAMTS1 in vivo has been reported to be expressed both by cancer and stromal cells [5]. Despite this, it is the cancer cells that secrete ADAMTS1 persistently to promote stromal cell remodelling of the ECM to promote cancer cell migration and invasion, which facilitates the pro-metastatic features of tumours [16]. Consistent with these published observations, we show that even though the epithelial tumour staining of ADAMTS1 in ovarian tumours is several-fold higher than the staining in stroma, it is staining in the stroma which progressively increases with tumour grades and stages making a significant difference in the ADAMTS1 staining between the low and high grades of tumours. These observations can be related to studies done in mouse models such as that in B16F1 murine model where a loss of tumour progression was noted when ADAMTS1 was genetically deleted in the host (mice) causing severe decrease in tumour growth and metastatic progression [72]. Although ADAMTS1 expression was provided by B16F1 tumour cells, the absence of ADAMTS1 expression in the host stroma significantly diminished B16F1 tumour growth and metastasis. However, knocking down ADAMTS1 expression in B16F1 tumour cells did not show these effects in xenograft models [72, 73]. These findings support the concept that it is the stromal ADAMTS1 expression in the vicinity of cancer cells that drives the tumorigenic role of cancer and ADAMTS1 in cancer cells only feeds the stromal expression of ADAMTS1 to drive cancer [74]. In line with that, in Adamts1(−/−) knockout PyMT mouse mammary tumour model development of smaller low-grade tumours with less pulmonary metastasis and significantly increased survival of mice compared to wild-type Adamts1(+/+) PyMT mice was observed [61]. Consistent with that, stable transfection of ADAMTS1 in bronchial epithelial tumour cells (BZR) and subcutaneous injection of these cells in immunodeficient mice accelerated the in vivo tumour growth and promoted stromal reaction characterised by myofibroblast infiltration and excessive matrix deposition due to increased production of metalloproteinase-13, fibronectin, TGFβ and interleukin-1β (IL-1B) [75]. Neutralising antibodies again TGFβ and IL-1β abrogated the chemotactic effects of ADAMTS1 expressing cells on the fibroblasts in vitro, indicating the effect of these factors on stromal infiltration in tumours [75]. These studies suggest that tumour cell migration and metastasis in vivo is facilitated by a crosstalk between ADAMTS1 expressing cancer and stromal cells, but it is the expression of ADAMTS1 in stroma that drives tumour progression.

However, in in vitro experimental models devoid of cancer-associated stroma, knock down of ADAMTS-1 expression in breast cancer cells demonstrated increased migration and invasion compared to other breast cancer cells in which the protease was not knocked down [22]. ADAMTS1 knocked down cells showed increased VEGF concentrations in conditioned medium in conjunction with enhanced expression of the VEGF receptor (VEGFR2). Pretreatment of these cells with a function-blocking antibody against VEGF reduced migration and invasion suggesting that sequestering of VEGF in conditions of ADAMTS1 knock down is involved with the enhanced migratory capacity of breast cancer cells. The role of VEGF in enhancing the migratory abilities of OC cell lines in response to ADAMTS1 knock down remains to be determined. At the same token, as mentioned above enhanced migration in OVCAR4 and OVCAR5 cell lines in response to ADAMTS1 knock down is consistent with CRISPR ADAMTS1 ablation in ES2 cell line; which was due to significantly increased Cdc42 GTP signals which strongly polarised the migrating cells at the leading edge of migratory front [31]. Contrary to that, NIH3T3 cells either with full length ADAMTS1 or mutation in the catalytic domain of ADAMTS1 exposed to the conditioned medium of ADAMTS1 enriched HT1080 cell line showed diminished migration compared to control untreated cells, suggesting that soluble ADAMTS1 in the environment of migrating cells impacts cancer cell migration and that catalytic domain of ADAMTS1 is not required for that diminished migration [31]. These observations are consistent with our data and is coherent with significant enhancement of Cdc42 GTPase activity under ADAMTS1 knocked down conditions. We also show that enhanced migration in response to ADAMTS1 knock down can be reversed by Cdc42 GTPase specific inhibitor ML141. However, no effect on reversing A-KD mediated loss of adhesion or initiation of MET was observed.

The findings overall suggest that the availability of soluble ADAMTS1 in the tumour microenvironment, which is dependent on the expression level of ADAMTS1 in cell lines may be an important requirement for determining of the migratory capacity of cancer cells. We also present other evidence; such as decreased adhesion and small rounded morphological changes inflicted on OC cells by ADAMTS1 KD may also facilitate the migratory capacity.

## Conclusion

In conclusion, we show that ADAMTS1 expression enhances as ovarian tumour progresses and that enhancement in expression is due to progressive increase in stromal ADAMTS1 expression. We also show that the functional outcome of ADAMTS1 expression in OC cells is context dependent and may be very much controlled by microenvironment sustaining the cancer cells with different effects in vivo compared to in vitro. Even though we have shown the roles of Cdc42 GTPase and VCAN in mediating ADAMTS1 knock down induced enhanced migration, reduced adhesion and epithelial cell plasticity in in vitro experimental models how these processes transcribe in vivo still needs to be studied in in vivo xenograft models. However, our in vitro studies show a direct reciprocal effect of ADAMTS1 knockdown (A-KD) on VCAN mRNA and protein expression in OVCAR4 and OVCAR5 A-KD cell lines. The fact that A-KD enhanced the mRNA expression of VCAN, with subsequent increase of migration and attenuation of adhesion in ovarian cancer cell lines and that these attributes were dampened by additional KD of VCAN expression, suggests a direct inverse link between ADAMTS1 and VCAN in in vitro functional assays. Our findings suggests that ADAMTS1 proteolysis of VCAN has crucial role in OC cells migratory and adhesive functions but only under conditions of low ADAMTS1 expression which enhances the expression of VCAN.

Even though the role of Cdc42 GTPase in epithelial cell plasticity in developmental biology has been shown to regulate cell polarity by strengthening cell–cell junctions [76] and E-Cadherin expression to sustain E-Cadherin mediated junctions anchored to actin cytoskeleton [77], we could only show reversal of migratory characteristics induced by A-KD in OC cell but no conclusive evidence of the reversal of A-KD mediated loss of adhesion and epithelial cell plasticity by using Cdc42 GTPase inhibitor. Knock down of VCAN in A-KD OC cell lines in vitro models. This may be due to specificity of the inhibitor in dampening and GTPase activity rather than direct effect on gene expression and also may be due to complex interplay of transcriptional, epigenetic, and signalling changes required to upregulate epithelial markers (e.g., E-Cad, EpCAM) and downregulate mesenchymal markers (e.g., CDH2, VIM) in cell culture models is beyond the scope of this study.

**Fig. 15:**
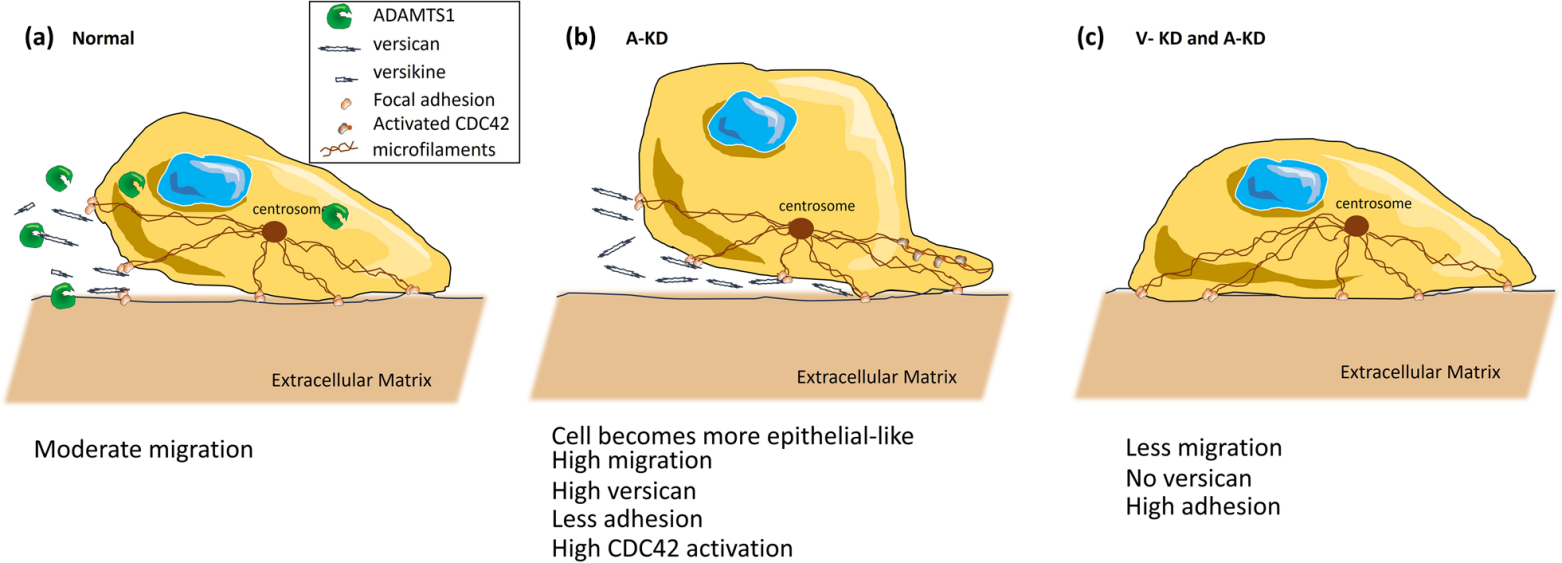
Model of ADAMTS1 knock down related functional changes in OC cell lines. **(a)** Normal functional status for OC cells. (**b)** Knock down of ADAMTS1 (A-KD) in OC cell lines induces enhanced Cdc42 GTPase activity and high intracellular VCAN production. This results in cells becoming more rounded, polarised epithelial-like cells with reduced adhesion and enhanced migration. (**c)** Knock down of VCAN in A-KD cells reverses the effect of increased migration and reduced adhesion imposed by A-KD in OC cells

Based on the novel findings in this study, we would like to propose that even though the expression of ADAMTS1 is significantly increased in high-grade and metastatic tumours compared to low grade/benign or primary tumours, relatively low expression of ADAMTS1 facilitates OC cell migration in vitro. This seemingly contradictory observation may represent the biology in mammary gland epithelium where intra-epithelial protrusions enriched in Cdc42 activity generate a coordinated force to push epithelial cell migration to attain branching morphogenesis [70]. In OC scenario, we demonstrate that this is possible in vitro, however, migration of OC cells with knocked down expression of ADAMTS1 requires further scrutiny in in vivo xenograft models. As the ability of cancer cells to switch inherent plasticity to facilitate migratory modes is a critical challenge preventing successful design of anti-cancer/metastatic therapies, targeting ovarian tumours with high expression of ADAMTS1 may have a profound positive impact on OC patients’ prognosis. A model hypothesising the in vitro scenario is explained in Fig. 15.

## Supplementary Information

Supplementary Material 1. Co-localization of ADAMTS1 with CA125 in benign and high-grade serous ovarian tumours. (**A**) Images are representative of the same high-grade, Stage III and Grade 2 serous ovarian tumour. Column 1 is representative of that tumour stained with H&E. Columns 2 and 3 represent corresponding CA-125 and ADAMTS1 staining of the same area of the tumour. Magnification, first row indicates =0.2X, scale green bar = 10 mm; second row a magnification 4.4X, scale purple bar = 500 µm, third row magnification 40X, scale black bar = 50 µm. (**B**) Matching sections show H&E stain, CA-125 and ADAMTS1 staining of benign and high-grade tumours. Magnification 40X, scale black bar = 50 µm

Supplementary Material 2. Localisation of ADAMTS1 siRNA in the ADAMTS1 gene. (**A**) ADAMTS1 gene siRNA gene location. (**B)** Preliminary data used to identify nM amount of siRNA for the best knock down of ADAMTS1 gene without producing off-target effects. 48h post transfection of ADAMTS1 and control siRNA, three independent siRNAs targeting ADAMTS1or pool siRNA was measured by qRT-PCR as described in the Methods. The experiment was repeated three times in triplicate in OVCAR4 and OVCAR5 cell lines. Graphs represent mean of total ± SEM. Significance is indicated by *p<0.05, **p<0.01, by one-way ANOVA. (**C**) Single fluorophore image corresponding to the immunofluorescence expression of ADAMTS1, in OVCAR5, OVCAR4 and ES-2 cell lines shown in Figure 5A. The images were obtained as described in Figure 4A. Magnification 20X; scale bar = 20 μm

Supplementary Material 3. Effect of ADAMTS-1 knock down on inhibitors and members of ADAM family. After 48 h of post transfection of ADAMTS1 siRNA the mRNA levels of (**A**) tissue inhibitors of metalloproteinases, (**B**) ADAMTS family members, and (**C**) ADAM family members, which share similar amino acid sequences with ADAMTS1, was deduced in the representative cell lines by qRT-PCR as described in Methods. Graphs represent amount of mRNA relative to 18S ± SEM derived from three experiments done in triplicate. P indicates parental cell line treated with transfection reagent, C are cells transfected with scrambled siRNA and A-KD is a representative of a pool of all three ADAMTS1 siRNAs knock down cells

Supplementary Material 4. Effect of ADAMTS-1 knock down on EMT-associated transcription factors. After 48 h of post transfection of ADAMTS1 siRNA the mRNA expression of SLUG, SNAIL, TWIST1, FZD4, FZD7 and SHC1 was deduced in the representative OVCAR4, OVCAR5 asnd ES2 cell lines by qRT-PCR as described in Methods. Graphs represent amount of mRNA relative to 18S ± SEM derived from three experiments done in triplicate. P indicates parental cell line treated with transfection reagent, C are cells transfected with scrambled siRNA and A-KD is a representative of a pool of all three ADAMTS1 siRNAs knock down cells. Graphs represent mean of total ± SEM. Significance is indicated by *p<0.05, **p<0.01, ***p<0.001 by one-way ANOVA

Supplementary Material 5. Representative three western blot images of OVCAR4 and OVCAR5 cell lines. Western blot was performed as described in Methods and material. Raw Western blot images of the Erk1/2 and phospho Erk1/2 bands from OVCAR4 and OVCAR5 cell lines transfected with ADAMTS1 siRNA

Supplementary Material 6. VCAN gene isoforms and their expression in OC cell lines. (**A**) VCAN isoforms showing the G1 domain, G2 central domain [where glycosaminoglycan (GAG) chains attach] and G3 domain. For each individual isoform the central domain G2 is differently spliced: V0 (containing both GAG alpha and GAG beta) , V1 (contains only GAG beta), while V2 (contains GAG alpha) and V3 lacks any GAG subdomain. The pink box indicates G1 domain; Yellow box indicates the GAG alpha sub domain; Blue box indicates GAG beta subdomain; colored lines indicate GAG chains attached to the GAG subdomains; Green box indicates G3 domain which contains the epidermal growth factor-like repeats, a lectin-like motif, and a complement-binding motif. Red box indicates isoforms previously described to be involved in OC. (**B**) The Ct (cycle threshold) value  in qRT-PCR of all the VCAN isoforms and G1 domain in OVCAR4, OVCAR5 and ES2 cell lines. Graphs represent only Ct values and error bars ± transfected with scrambled siRNA and A-KD is a representative of a pool of all three ADAMTS1 siRNAs knock down cells. Significance is indicated by *p < 0.05 and **p < 0.01, one-way ANOVA using Tukey’s multiple comparison test. A very high Ct (usually 40+) often suggest that the gene is not expressed by qRT-PCR

Supplementary Material 7. Effect of ML141 on Cdc42 GTPase activity. ML141was used to inhibit Cdc42 GTPase activation induced by ADAMTS1 siRNA. 48 hrs post-transfection of ADAMTS1 siRNA and control siRNA. C are cells transfected with scrambled siRNA and A-KD is a representative of a pool of all three ADAMTS1 siRNAs knocked down cells. 5-10 µM ML141 decreased Cdc42 activity levels back to OVCAR4 endogenous levels. Each bar indicates mean ±SEM, derived from one experiment done in duplicate

Supplementary Material 8. Effect of ML141 of Cdc42 activity on epithelial plasticity. The mRNA expression EMT inducerin OVCAR4 and OVCAR5 control and ADAMTS1 siRNA treated cells for 24 h with 10µM of the Cdc42 inhibitor, was deduced by qRT-PCR. The experiment was repeated three times in triplicate. Graphs represent mean of total ± SEM. Significance is indicated by *p<0.05, and **p<0.01, one-way ANOVA

Supplementary Material 9. Effect of ML141 on ADAMTS1 and VCAN expression. The mRNA expression  of ADAMTS1 and VCAN in OVCAR4 and OVCAR5 control and ADAMTS1 siRNA treated cells for 24 h with 10 µM of the Cdc42 inhibitor, was assessed by qRT-PCR. The experiment was repeated three times in triplicate. Graphs represent mean of total ± SEM. Graphs represent mean of total ± SEM. Significance is indicated by *p<0.05, and **p<0.01, one-way ANOVA

Supplementary Material 10. List of 5 Tables

## Data Availability

The data presented in the study are not publicly available. However, the data can be made available from the corresponding author on a reasonable request.

## Abbreviations

ADAMTS1: A disintegrin and metalloproneinase with thrombospondin motifs
OC: Ovarian cancer
ECM: Extracellular matrix
EMT: Epithelial mesenchymal transition
MET: Mesenchymal epithelial transition
A-KD: ADAMTS1 knock down
C: Control
P: Parental
CA125: Cancer antigen 125
E-Cad: E-Cadherin
N-Cad: N-Cadherin
VIM: Vimentin
EGFR: Epidermal Growth Factor Receptor
TGFβ: Transforming growth factor beta
MMP: Matrix metalloproteinase
MT-MMP: Membrane type matrix metalloproteinase
SDC4: Syndecan-4
VCAN: Versican
FBL1: Fibulin-1
MTT: 3- (4,5 Dimethylthiazol-2-yl)-2,5-diphenyl tetrazolium bromide
DAPI: 4,6-Diamidino-2-phenylindole
qRT-PCR: Quantitative reverse transcription polymerase chain reaction
ELISA: Enzyme linked immunosorbent assay
SDS-PAGE: Sodium dodecyl sulfate polyacrylamide gel electrophoresis
PVDF: Polyvinylidene difluoride
PBS: Phosphate buffer saline
TCGA: The Cancer Genome Atlas

## References

[1] Sung H, Ferlay J, Siegel RL, Laversanne M, Soerjomataram I, Jemal A, et al. Global cancer statistics 2020: GLOBOCAN estimates of incidence and mortality worldwide for 36 cancers in 185 countries. CA Cancer J Clin. 2021;71(3):209–49. 33538338 10.3322/caac.21660

[2] Klotz DM, Wimberger P. Cells of origin of ovarian cancer: ovarian surface epithelium or fallopian tube? Arch Gynecol Obstet. 2017;296(6):1055–62. 28940023 10.1007/s00404-017-4529-z

[3] Lheureux S, Gourley C, Vergote I, Oza AM. Epithelial ovarian cancer. Lancet. 2019;393(10177):1240–53. 30910306 10.1016/S0140-6736(18)32552-2

[4] Wesley T, Berzins S, Kannourakis G, Ahmed N. The attributes of plakins in cancer and disease: perspectives on ovarian cancer progression, chemoresistance and recurrence. Cell Commun Signal. 2021;19(1):55. 34001250 10.1186/s12964-021-00726-x PMC8127266

[5] Ahmed N, Stenvers KL. Getting to know ovarian cancer ascites: opportunities for targeted therapy-based translational research. Front Oncol. 2013;3:256. 24093089 10.3389/fonc.2013.00256 PMC3782691

[6] Novak C, Horst E, Mehta G. Review: mechanotransduction in ovarian cancer: shearing into the unknown. APL Bioeng. 2018;2(3):031701. 31069311 10.1063/1.5024386 PMC6481715

[7] Hyler AR, Baudoin NC, Brown MS, Stremler MA, Cimini D, Davalos RV, et al. Fluid shear stress impacts ovarian cancer cell viability, subcellular organization, and promotes genomic instability. PLoS ONE. 2018;13(3):e0194170. 29566010 10.1371/journal.pone.0194170 PMC5864000

[8] Compton SLE, Pyne ES, Liu L, Guinan J, Shea AA, Grieco JP, et al. Adaptation of metabolism to multicellular aggregation, hypoxia and obese stromal cell incorporation as potential measure of survival of ovarian metastases. Exp Cell Res. 2021;399(1):112397. 33338477 10.1016/j.yexcr.2020.112397

[9] Yu Y, Lyu C, Li X, Yang L, Wang J, Li H, et al. Remodeling of tumor microenvironment by extracellular matrix protein 1a differentially regulates ovarian cancer metastasis. Cancer Lett. 2024;596:217022. 38849014 10.1016/j.canlet.2024.217022

[10] Bacchetti R, Yuan S, Rainero E. ADAMTS proteases: Their multifaceted role in the regulation of cancer metastasis. Dis Res. 2024;4(1):40–52. 38948119 10.54457/DR.202401004 PMC7616120

[11] Tan IA, Ricciardelli C, Russell DL. The metalloproteinase ADAMTS1: a comprehensive review of its role in tumorigenic and metastatic pathways. Int J Cancer. 2013;133(10):2263–76. 23444028 10.1002/ijc.28127

[12] Hubmacher D, Apte SS. ADAMTS proteins as modulators of microfibril formation and function. Matrix Biol. 2015;47:34–43. 25957949 10.1016/j.matbio.2015.05.004 PMC4731137

[13] Kelwick R, Desanlis I, Wheeler GN, Edwards DR. The ADAMTS (a disintegrin and metalloproteinase with thrombospondin motifs) family. Genome Biol. 2015;16(1):113. 26025392 10.1186/s13059-015-0676-3 PMC4448532

[14] Ween MP, Hummitzsch K, Rodgers RJ, Oehler MK, Ricciardelli C. Versican induces a pro-metastatic ovarian cancer cell behavior which can be inhibited by small hyaluronan oligosaccharides. Clin Exp Metastasis. 2011;28(2):113–25. 21153687 10.1007/s10585-010-9363-7

[15] Desjardins M, Xie J, Gurler H, Muralidhar GG, Sacks JD, Burdette JE, et al. Versican regulates metastasis of epithelial ovarian carcinoma cells and spheroids. J Ovarian Res. 2014;7:70. 24999371 10.1186/1757-2215-7-70 PMC4081460

[16] Ricciardelli C, Russell DL, Ween MP, Mayne K, Suwiwat S, Byers S, et al. Formation of hyaluronan- and versican-rich pericellular matrix by prostate cancer cells promotes cell motility. J Biol Chem. 2007;282(14):10814–25. 17293599 10.1074/jbc.M606991200

[17] Russo A, Yang Z, Heyrman GM, Cain BP, Lopez Carrero A, Isenberg BC, et al. Versican secreted by the ovary links ovulation and migration in fallopian tube derived serous cancer. Cancer Lett. 2022;543:215779. 35697329 10.1016/j.canlet.2022.215779 PMC10134877

[18] Rodriguez-Manzaneque JC, Carpizo D, Plaza-Calonge MC, Torres-Collado AX, Thai SN, Simons M, et al. Cleavage of syndecan-4 by ADAMTS1 provokes defects in adhesion. Int J Biochem Cell Biol. 2009;41(4):800–10. 18775505 10.1016/j.biocel.2008.08.014 PMC3807939

[19] Elfenbein A, Simons M. Syndecan-4 signaling at a glance. J Cell Sci. 2013;126(Pt 17):3799–804. 23970415 10.1242/jcs.124636 PMC3757327

[20] Lambert J, Makin K, Akbareian S, Johnson R, Alghamdi AAA, Robinson SD, et al. ADAMTS-1 and syndecan-4 intersect in the regulation of cell migration and angiogenesis. J Cell Sci. 2020. 10.1242/jcs.235762. 32269093 10.1242/jcs.235762 PMC7157938

[21] Choi JE, Kim DS, Kim EJ, Chae MH, Cha SI, Kim CH, et al. Aberrant methylation of ADAMTS1 in non-small cell lung cancer. Cancer Genet Cytogenet. 2008;187(2):80–4. 19027488 10.1016/j.cancergencyto.2008.08.001

[22] Freitas VM, do Amaral JB, Silva TA, Santos ES, Mangone FR, Pinheiro JJ, et al. Decreased expression of ADAMTS-1 in human breast tumors stimulates migration and invasion. Mol Cancer. 2013;12:2. 23289900 10.1186/1476-4598-12-2 PMC3600045

[23] Wang S, Zhang J, Wang K, Zhao Y, Liu D. ADAMTS1 as potential prognostic biomarker promotes malignant invasion of glioma. Int J Clin Oncol. 2023;28(1):52–68. 36371587 10.1007/s10147-022-02268-9

[24] Lima MA, Dos Santos L, Turri JA, Nonogaki S, Buim M, Lima JF, et al. Prognostic value of ADAMTS proteases and their substrates in epithelial ovarian cancer. Pathobiology. 2016;83(6):316–26. 27359117 10.1159/000446244

[25] Bourd-Boittin K, Bonnier D, Leyme A, Mari B, Tuffery P, Samson M, et al. Protease profiling of liver fibrosis reveals the ADAM metallopeptidase with thrombospondin type 1 motif, 1 as a central activator of transforming growth factor beta. Hepatology. 2011;54(6):2173–84. 21826695 10.1002/hep.24598

[26] Lee NV, Rodriguez-Manzaneque JC, Thai SN, Twal WO, Luque A, Lyons KM, et al. Fibulin-1 acts as a cofactor for the matrix metalloprotease ADAMTS-1. J Biol Chem. 2005;280(41):34796–804. 16061471 10.1074/jbc.M506980200

[27] Liu YJ, Xu Y, Yu Q. Full-length ADAMTS-1 and the ADAMTS-1 fragments display pro- and antimetastatic activity, respectively. Oncogene. 2006;25(17):2452–67. 16314835 10.1038/sj.onc.1209287 PMC2759703

[28] Hu X, Jiang C, Hu N, Hong S. ADAMTS1 induces epithelial-mesenchymal transition pathway in non-small cell lung cancer by regulating TGF-beta. Aging. 2023;15(6):2097–114. 36947712 10.18632/aging.204594 PMC10085599

[29] Chien MH, Yang YC, Ho KH, Ding YF, Chen LH, Chiu WK, et al. Cyclic increase in the ADAMTS1-L1CAM-EGFR axis promotes the EMT and cervical lymph node metastasis of oral squamous cell carcinoma. Cell Death Dis. 2024;15(1):82. 38263290 10.1038/s41419-024-06452-9 PMC10805752

[30] Wen YC, Lin YW, Ho KH, Yang YC, Lai FR, Chu CY, et al. The oncogenic ADAMTS1-VCAN-EGFR cyclic axis drives anoikis resistance and invasion in renal cell carcinoma. Cell Mol Biol Lett. 2024;29(1):126. 39333870 10.1186/s11658-024-00643-0 PMC11429190

[31] de Assis Lima M, da Silva SV, Serrano-Garrido O, Hulsemann M, Santos-Neres L, Rodriguez-Manzaneque JC, et al. Metalloprotease ADAMTS-1 decreases cell migration and invasion modulating the spatiotemporal dynamics of Cdc42 activity. Cell Signal. 2021;77:109827. 33161094 10.1016/j.cellsig.2020.109827 PMC7723338

[32] Raza A, Hoque A, Luwor R, Escalona RM, Kelly J, Sharma R, et al. Enhanced expression of mitochondrial Magmas protein in ovarian carcinomas: Magmas inhibition facilitates antitumour effects, signifying a novel approach for ovarian cancer treatment. Cells. 2025. 10.3390/cells14090655. 40358179 10.3390/cells14090655 PMC12071367

[33] Escalona RM, Bilandzic M, Western P, Kadife E, Kannourakis G, Findlay JK, et al. TIMP-2 regulates proliferation, invasion and STAT3-mediated cancer stem cell-dependent chemoresistance in ovarian cancer cells. BMC Cancer. 2020;20(1):960. 33023532 10.1186/s12885-020-07274-6 PMC7542139

[34] Escalona RM, Chu S, Kadife E, Kelly JK, Kannourakis G, Findlay JK, et al. Knock down of TIMP-2 by siRNA and CRISPR/Cas9 mediates diverse cellular reprogramming of metastasis and chemosensitivity in ovarian cancer. Cancer Cell Int. 2022;22(1):422. 36585738 10.1186/s12935-022-02838-x PMC9805260

[35] Lokman NA, Pyragius CE, Ruszkiewicz A, Oehler MK, Ricciardelli C. Annexin A2 and S100A10 are independent predictors of serous ovarian cancer outcome. Transl Res. 2016;171:83–95 (**e81-82**). 26925708 10.1016/j.trsl.2016.02.002

[36] Bankhead P, Loughrey MB, Fernandez JA, Dombrowski Y, McArt DG, Dunne PD, et al. QuPath: open source software for digital pathology image analysis. Sci Rep. 2017;7(1):16878. 29203879 10.1038/s41598-017-17204-5 PMC5715110

[37] Latifi A, Luwor RB, Bilandzic M, Nazaretian S, Stenvers K, Pyman J, et al. Isolation and characterization of tumor cells from the ascites of ovarian cancer patients: molecular phenotype of chemoresistant ovarian tumors. PLoS ONE. 2012;7(10):e46858. 23056490 10.1371/journal.pone.0046858 PMC3466197

[38] Escalona RM, Kannourakis G, Findlay JK, Ahmed N. Expression of TIMPs and MMPs in ovarian tumors, ascites, ascites-derived cells, and cancer cell lines: characteristic modulatory response before and after chemotherapy treatment. Front Oncol. 2021;11:796588. 35047406 10.3389/fonc.2021.796588 PMC8762252

[39] Uhlen M, Fagerberg L, Hallstrom BM, Lindskog C, Oksvold P, Mardinoglu A, et al. Proteomics. Tissue-based map of the human proteome. Science. 2015;347(6220):1260419. 25613900 10.1126/science.1260419

[40] Park SJ, Yoon BH, Kim SK, Kim SY. GENT2: an updated gene expression database for normal and tumor tissues. BMC Med Genomics. 2019;12(Suppl 5):101. 31296229 10.1186/s12920-019-0514-7 PMC6624177

[41] Price ZK, Lokman NA, Sugiyama M, Koya Y, Yoshihara M, Oehler MK, et al. Disabled-2: a protein up-regulated by high molecular weight hyaluronan has both tumor promoting and tumor suppressor roles in ovarian cancer. Cell Mol Life Sci. 2023;80(11):320. 37815603 10.1007/s00018-023-04972-9 PMC10564841

[42] Gyorffy B. Discovery and ranking of the most robust prognostic biomarkers in serous ovarian cancer. Geroscience. 2023;45(3):1889–98. 36856946 10.1007/s11357-023-00742-4 PMC10400493

[43] Gyorffy B. Integrated analysis of public datasets for the discovery and validation of survival-associated genes in solid tumors. Innovation. 2024;5(3):100625. 38706955 10.1016/j.xinn.2024.100625 PMC11066458

[44] Blayney JK, Davison T, McCabe N, Walker S, Keating K, Delaney T, et al. Prior knowledge transfer across transcriptional data sets and technologies using compositional statistics yields new mislabelled ovarian cell line. Nucleic Acids Res. 2016;44(17):e137. 27353327 10.1093/nar/gkw578 PMC5041471

[45] Lorenzi PL, Reinhold WC, Varma S, Hutchinson AA, Pommier Y, Chanock SJ, et al. DNA fingerprinting of the NCI-60 cell line panel. Mol Cancer Ther. 2009;8(4):713–24. 19372543 10.1158/1535-7163.MCT-08-0921 PMC4020356

[46] Kwok AL, Wong OG, Wong ES, Tsun OK, Chan KK, Cheung AN. Caution over use of ES2 as a model of ovarian clear cell carcinoma. J Clin Pathol. 2014;67(10):921–2. 25049276 10.1136/jclinpath-2014-202430

[47] Willemsen M, Krebbers G, Bekkenk MW, Teunissen MBM, Luiten RM. Improvement of Opal multiplex immunofluorescence workflow for human tissue sections. J Histochem Cytochem. 2021;69(5):339–46. 33797290 10.1369/00221554211007793 PMC8091416

[48] Shield K, Riley C, Quinn MA, Rice GE, Ackland ML, Ahmed N. Alpha2beta1 integrin affects metastatic potential of ovarian carcinoma spheroids by supporting disaggregation and proteolysis. J Carcinog. 2007;6:11. 17567918 10.1186/1477-3163-6-11 PMC1929068

[49] Schlie-Wolter S, Ngezahayo A, Chichkov BN. The selective role of ECM components on cell adhesion, morphology, proliferation and communication in vitro. Exp Cell Res. 2013;319(10):1553–61. 23588204 10.1016/j.yexcr.2013.03.016

[50] Lee CJ, Jang TY, Jeon SE, Yun HJ, Cho YH, Lim DY, et al. The dysadherin/MMP9 axis modifies the extracellular matrix to accelerate colorectal cancer progression. Nat Commun. 2024;15(1):10422. 39613801 10.1038/s41467-024-54920-9 PMC11607440

[51] Winkler J, Abisoye-Ogunniyan A, Metcalf KJ, Werb Z. Concepts of extracellular matrix remodelling in tumour progression and metastasis. Nat Commun. 2020;11(1):5120. 33037194 10.1038/s41467-020-18794-x PMC7547708

[52] Luo W, Shi Q, Han M, Zhang Z, Reiter RJ, Ashrafizadeh M, et al. TGF-beta-driven EMT in cancer progression and drug resistance. Cytokine Growth Factor Rev. 2025;85:11–25. 40436672 10.1016/j.cytogfr.2025.05.004

[53] Misra A, Pandey C, Sze SK, Thanabalu T. Hypoxia activated EGFR signaling induces epithelial to mesenchymal transition (EMT). PLoS ONE. 2012;7(11):e49766. 23185433 10.1371/journal.pone.0049766 PMC3504094

[54] Xie L, Law BK, Chytil AM, Brown KA, Aakre ME, Moses HL. Activation of the Erk pathway is required for TGF-beta1-induced EMT in vitro. Neoplasia. 2004;6(5):603–10. 15548370 10.1593/neo.04241 PMC1531665

[55] Schinke H, Shi E, Lin Z, Quadt T, Kranz G, Zhou J, et al. A transcriptomic map of EGFR-induced epithelial-to-mesenchymal transition identifies prognostic and therapeutic targets for head and neck cancer. Mol Cancer. 2022;21(1):178. 36076232 10.1186/s12943-022-01646-1 PMC9454230

[56] Fontanil T, Mohamedi Y, Cobo T, Cal S, Obaya AJ. Novel associations within the tumor microenvironment: fibulins meet ADAMTSs. Front Oncol. 2019;9:796. 31508361 10.3389/fonc.2019.00796 PMC6714394

[57] Nandadasa S, Foulcer S, Apte SS. The multiple, complex roles of versican and its proteolytic turnover by ADAMTS proteases during embryogenesis. Matrix Biol. 2014;35:34–41. 24444773 10.1016/j.matbio.2014.01.005 PMC5525047

[58] Ghosh S, Albitar L, LeBaron R, Welch WR, Samimi G, Birrer MJ, et al. Up-regulation of stromal versican expression in advanced stage serous ovarian cancer. Gynecol Oncol. 2010;119(1):114–20. 20619446 10.1016/j.ygyno.2010.05.029 PMC3000175

[59] Arias-Romero LE, Chernoff J. Targeting Cdc42 in cancer. Expert Opin Ther Targets. 2013;17(11):1263–73. 23957315 10.1517/14728222.2013.828037 PMC3937847

[60] Li X, Tan C, Fu X, Qiu J, Shen W, Xu Z, et al. Disrupting Cdc42 activation-driven filopodia formation with low-intensity ultrasound and microbubbles: a novel strategy to block ovarian cancer metastasis. Colloids Surf B Biointerfaces. 2025;253:114724. 40300280 10.1016/j.colsurfb.2025.114724

[61] Ricciardelli C, Frewin KM, Tan IA, Williams ED, Opeskin K, Pritchard MA, et al. The ADAMTS1 protease gene is required for mammary tumor growth and metastasis. Am J Pathol. 2011;179(6):3075–85. 22001177 10.1016/j.ajpath.2011.08.021 PMC3260838

[62] Russell DL, Doyle KM, Ochsner SA, Sandy JD, Richards JS. Processing and localization of ADAMTS-1 and proteolytic cleavage of versican during cumulus matrix expansion and ovulation. J Biol Chem. 2003;278(43):42330–9. 12907688 10.1074/jbc.M300519200

[63] Silva SV, Lima MA, Cella N, Jaeger RG, Freitas VM. ADAMTS-1 is found in the nuclei of normal and tumoral breast cells. PLoS ONE. 2016;11(10):e0165061. 27764205 10.1371/journal.pone.0165061 PMC5072708

[64] Silva SV, Lima MA, Hodgson L, Freitas VM, Rodriguez-Manzaneque JC. ADAMTS-1 has nuclear localization in cells with epithelial origin and leads to decreased cell migration. Exp Cell Res. 2023;433(2):113852. 37951335 10.1016/j.yexcr.2023.113852 PMC10841765

[65] Carthy JM, Abraham T, Meredith AJ, Boroomand S, McManus BM. Versican localizes to the nucleus in proliferating mesenchymal cells. Cardiovasc Pathol. 2015;24(6):368–74. 26395512 10.1016/j.carpath.2015.07.010

[66] Harikrishnan K, Joshi O, Madangirikar S, Balasubramanian N. Cell derived matrix fibulin-1 associates with epidermal growth factor receptor to inhibit its activation, localization and function in lung cancer Calu-1 cells. Front Cell Dev Biol. 2020;8:522. 32719793 10.3389/fcell.2020.00522 PMC7348071

[67] Twal WO, Czirok A, Hegedus B, Knaak C, Chintalapudi MR, Okagawa H, et al. Fibulin-1 suppression of fibronectin-regulated cell adhesion and motility. J Cell Sci. 2001;114(Pt 24):4587–98. 11792823 10.1242/jcs.114.24.4587

[68] Ewald AJ, Brenot A, Duong M, Chan BS, Werb Z. Collective epithelial migration and cell rearrangements drive mammary branching morphogenesis. Dev Cell. 2008;14(4):570–81. 18410732 10.1016/j.devcel.2008.03.003 PMC2773823

[69] Friedl P, Gilmour D. Collective cell migration in morphogenesis, regeneration and cancer. Nat Rev Mol Cell Biol. 2009;10(7):445–57. 19546857 10.1038/nrm2720

[70] Lu P, Lu Y. Born to run? Diverse modes of epithelial migration. Front Cell Dev Biol. 2021;9:704939. 34540829 10.3389/fcell.2021.704939 PMC8448196

[71] Sheng W, Wang G, La Pierre DP, Wen J, Deng Z, Wong CK, et al. Versican mediates mesenchymal-epithelial transition. Mol Biol Cell. 2006;17(4):2009–20. 16452631 10.1091/mbc.E05-10-0951 PMC1415306

[72] Fernandez-Rodriguez R, Rodriguez-Baena FJ, Martino-Echarri E, Peris-Torres C, Plaza-Calonge MDC, Rodriguez-Manzaneque JC. Stroma-derived but not tumor ADAMTS1 is a main driver of tumor growth and metastasis. Oncotarget. 2016;7(23):34507–19. 27120788 10.18632/oncotarget.8922 PMC5085172

[73] Caracuel-Peramos R, Rodriguez-Baena FJ, Redondo-Garcia S, Villatoro-Garcia JA, Garcia-Munoz A, Peris-Torres C, et al. Loss of the extracellular protease ADAMTS1 reveals an antitumorigenic program involving the action of NIDOGEN-1 on macrophage polarization. Oncoimmunology. 2025;14(1):2508057. 40401531 10.1080/2162402X.2025.2508057 PMC12101600

[74] Tan IA, Frewin K, Ricciardelli C, Russell DL. ADAMTS1 promotes adhesion to extracellular matrix proteins and predicts prognosis in early stage breast cancer patients. Cell Physiol Biochem. 2019;52(6):1553–68. 31135123 10.33594/000000108

[75] Rocks N, Paulissen G, Quesada-Calvo F, Munaut C, Gonzalez ML, Gueders M, et al. ADAMTS-1 metalloproteinase promotes tumor development through the induction of a stromal reaction in vivo. Cancer Res. 2008;68(22):9541–50. 19010931 10.1158/0008-5472.CAN-08-0548

[76] Stengel K, Zheng Y. Cdc42 in oncogenic transformation, invasion, and tumorigenesis. Cell Signal. 2011;23(9):1415–23. 21515363 10.1016/j.cellsig.2011.04.001 PMC3115433

[77] Chu YS, Thomas WA, Eder O, Pincet F, Perez E, Thiery JP, et al. Force measurements in E-cadherin-mediated cell doublets reveal rapid adhesion strengthened by actin cytoskeleton remodeling through Rac and Cdc42. J Cell Biol. 2004;167(6):1183–94. 15596540 10.1083/jcb.200403043 PMC2172605

