# Supplementary figures and images for: Enhanced expression of ADAMTS1 in ovarian carcinomas: loss of ADAMTS1 expression instigates cellular reprogramming of extracellular matrix ensuing altered plasticity, augmented migration and attenuated adhesion
Source: J Biomed Sci. 2026 Jun 22;33:67. doi: 10.1186/s12929-026-01260-z (PMC13289130; doi:10.1186/s12929-026-01260-z)

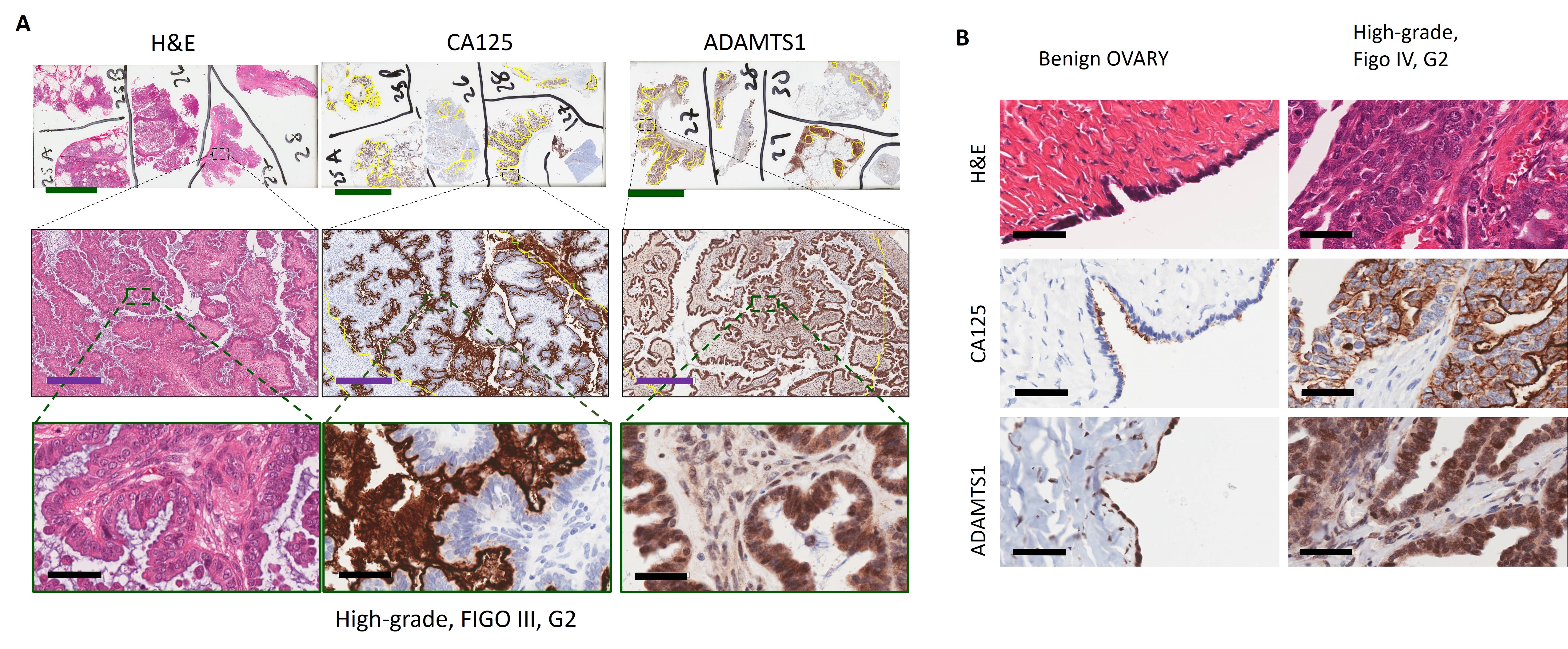

Supplement: Supplementary file 1 — Supplementary Material 1. Co-localization of ADAMTS1 with CA125 in benign and high-grade serous ovarian tumours. (A) Images are representative of the same high-grade, Stage III and Grade 2 serous ovarian tumour. Column 1 is representative of that tumour stained with H&E. Columns 2 and 3 represent corresponding CA-125 and ADAMTS1 staining of the same area of the tumour. Magnification, first row indicates =0.2X, scale green bar = 10 mm; second row a magnification 4.4X, scale purple bar = 500 µm, third row magnification 40X, scale black bar = 50 µm. (B) Matching sections show H&E stain, CA-125 and ADAMTS1 staining of benign and high-grade tumours. Magnification 40X, scale black bar = 50 µm [file 12929_2026_1260_MOESM1_ESM.jpg]

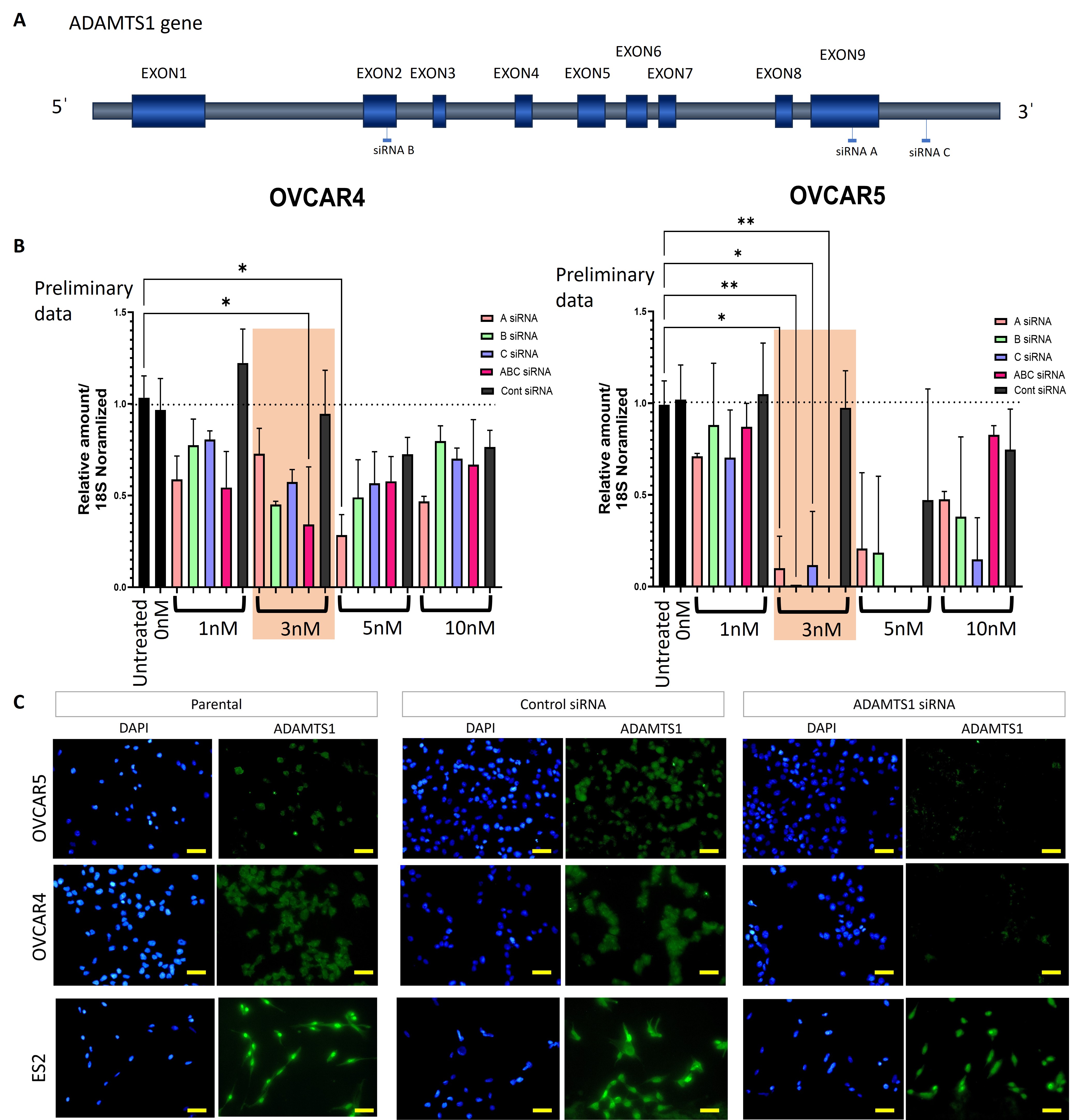

Supplement: Supplementary file 2 — Supplementary Material 2. Localisation of ADAMTS1 siRNA in the ADAMTS1 gene. (A) ADAMTS1 gene siRNA gene location. (B) Preliminary data used to identify nM amount of siRNA for the best knock down of ADAMTS1 gene without producing off-target effects. 48h post transfection of ADAMTS1 and control siRNA, three independent siRNAs targeting ADAMTS1or pool siRNA was measured by qRT-PCR as described in the Methods. The experiment was repeated three times in triplicate in OVCAR4 and OVCAR5 cell lines. Graphs represent mean of total ± SEM. Significance is indicated by *p<0.05, **p<0.01, by one-way ANOVA. (C) Single fluorophore image corresponding to the immunofluorescence expression of ADAMTS1, in OVCAR5, OVCAR4 and ES-2 cell lines shown in Figure 5A. The images were obtained as described in Figure 4A. Magnification 20X; scale bar = 20 μm [file 12929_2026_1260_MOESM2_ESM.jpg]

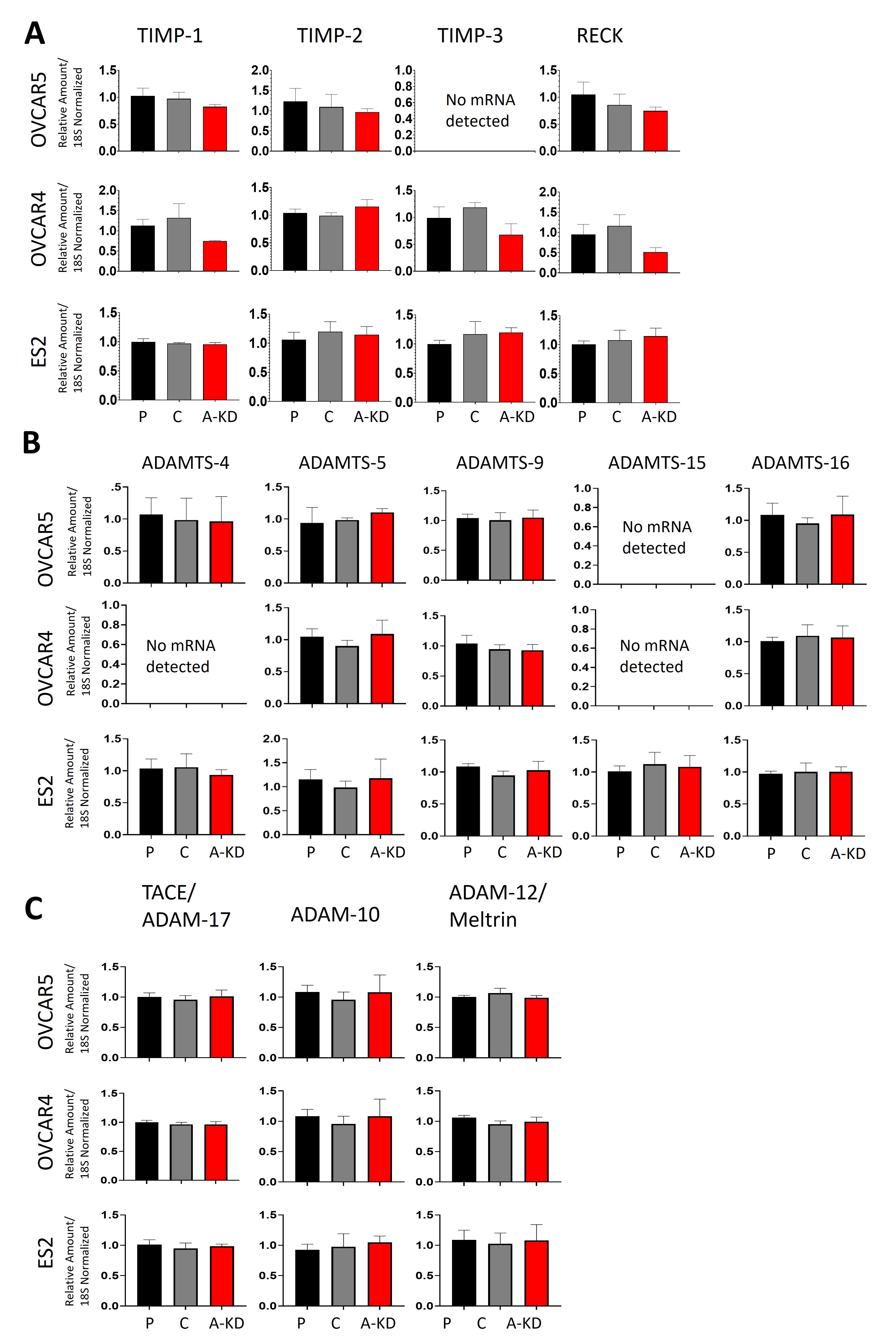

Supplement: Supplementary file 3 — Supplementary Material 3. Effect of ADAMTS-1 knock down on inhibitors and members of ADAM family. After 48 h of post transfection of ADAMTS1 siRNA the mRNA levels of (A) tissue inhibitors of metalloproteinases, (B) ADAMTS family members, and (C) ADAM family members, which share similar amino acid sequences with ADAMTS1, was deduced in the representative cell lines by qRT-PCR as described in Methods. Graphs represent amount of mRNA relative to 18S ± SEM derived from three experiments done in triplicate. P indicates parental cell line treated with transfection reagent, C are cells transfected with scrambled siRNA and A-KD is a representative of a pool of all three ADAMTS1 siRNAs knock down cells [file 12929_2026_1260_MOESM3_ESM.jpg]

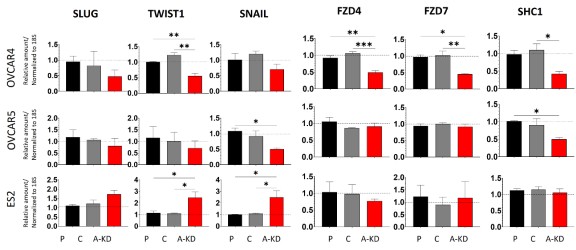

Supplement: Supplementary file 4 — Supplementary Material 4. Effect of ADAMTS-1 knock down on EMT-associated transcription factors. After 48 h of post transfection of ADAMTS1 siRNA the mRNA expression of SLUG, SNAIL, TWIST1, FZD4, FZD7 and SHC1 was deduced in the representative OVCAR4, OVCAR5 asnd ES2 cell lines by qRT-PCR as described in Methods. Graphs represent amount of mRNA relative to 18S ± SEM derived from three experiments done in triplicate. P indicates parental cell line treated with transfection reagent, C are cells transfected with scrambled siRNA and A-KD is a representative of a pool of all three ADAMTS1 siRNAs knock down cells. Graphs represent mean of total ± SEM. Significance is indicated by *p<0.05, **p<0.01, ***p<0.001 by one-way ANOVA [file 12929_2026_1260_MOESM4_ESM.jpg]

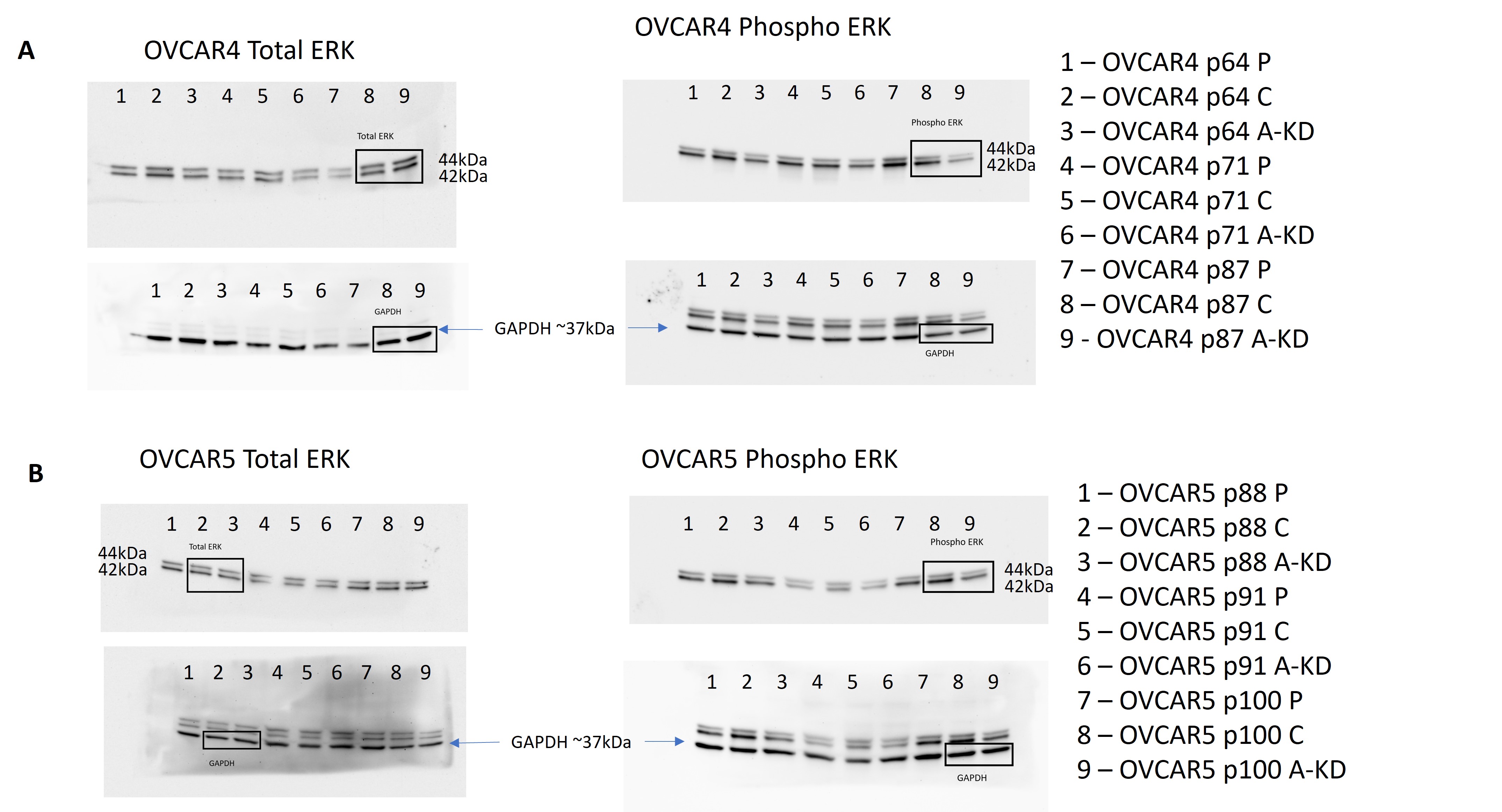

Supplement: Supplementary file 5 — Supplementary Material 5. Representative three western blot images of OVCAR4 and OVCAR5 cell lines. Western blot was performed as described in Methods and material. Raw Western blot images of the Erk1/2 and phospho Erk1/2 bands from OVCAR4 and OVCAR5 cell lines transfected with ADAMTS1 siRNA [file 12929_2026_1260_MOESM5_ESM.jpg]

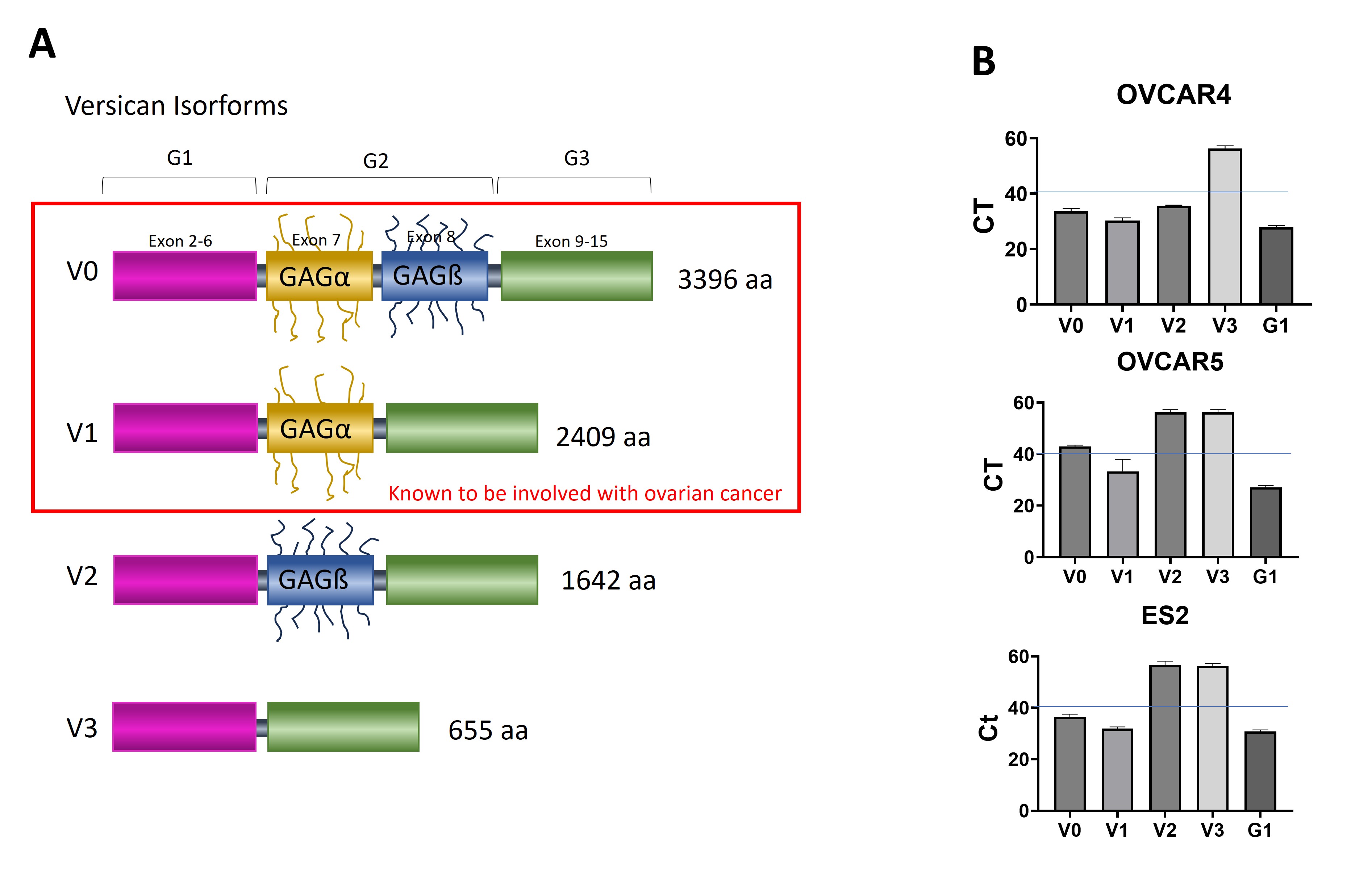

Supplement: Supplementary file 6 — Supplementary Material 6. VCAN gene isoforms and their expression in OC cell lines. (A) VCAN isoforms showing the G1 domain, G2 central domain [where glycosaminoglycan (GAG) chains attach] and G3 domain. For each individual isoform the central domain G2 is differently spliced: V0 (containing both GAG alpha and GAG beta) , V1 (contains only GAG beta), while V2 (contains GAG alpha) and V3 lacks any GAG subdomain. The pink box indicates G1 domain; Yellow box indicates the GAG alpha sub domain; Blue box indicates GAG beta subdomain; colored lines indicate GAG chains attached to the GAG subdomains; Green box indicates G3 domain which contains the epidermal growth factor-like repeats, a lectin-like motif, and a complement-binding motif. Red box indicates isoforms previously described to be involved in OC. (B) The Ct (cycle threshold) value in qRT-PCR of all the VCAN isoforms and G1 domain in OVCAR4, OVCAR5 and ES2 cell lines. Graphs represent only Ct values and error bars ± transfected with scrambled siRNA and A-KD is a representative of a pool of all three ADAMTS1 siRNAs knock down cells. Significance is indicated by *p < 0.05 and **p < 0.01, one-way ANOVA using Tukey’s multiple comparison test. A very high Ct (usually 40+) often suggest that the gene is not expressed by qRT-PCR [file 12929_2026_1260_MOESM6_ESM.jpg]

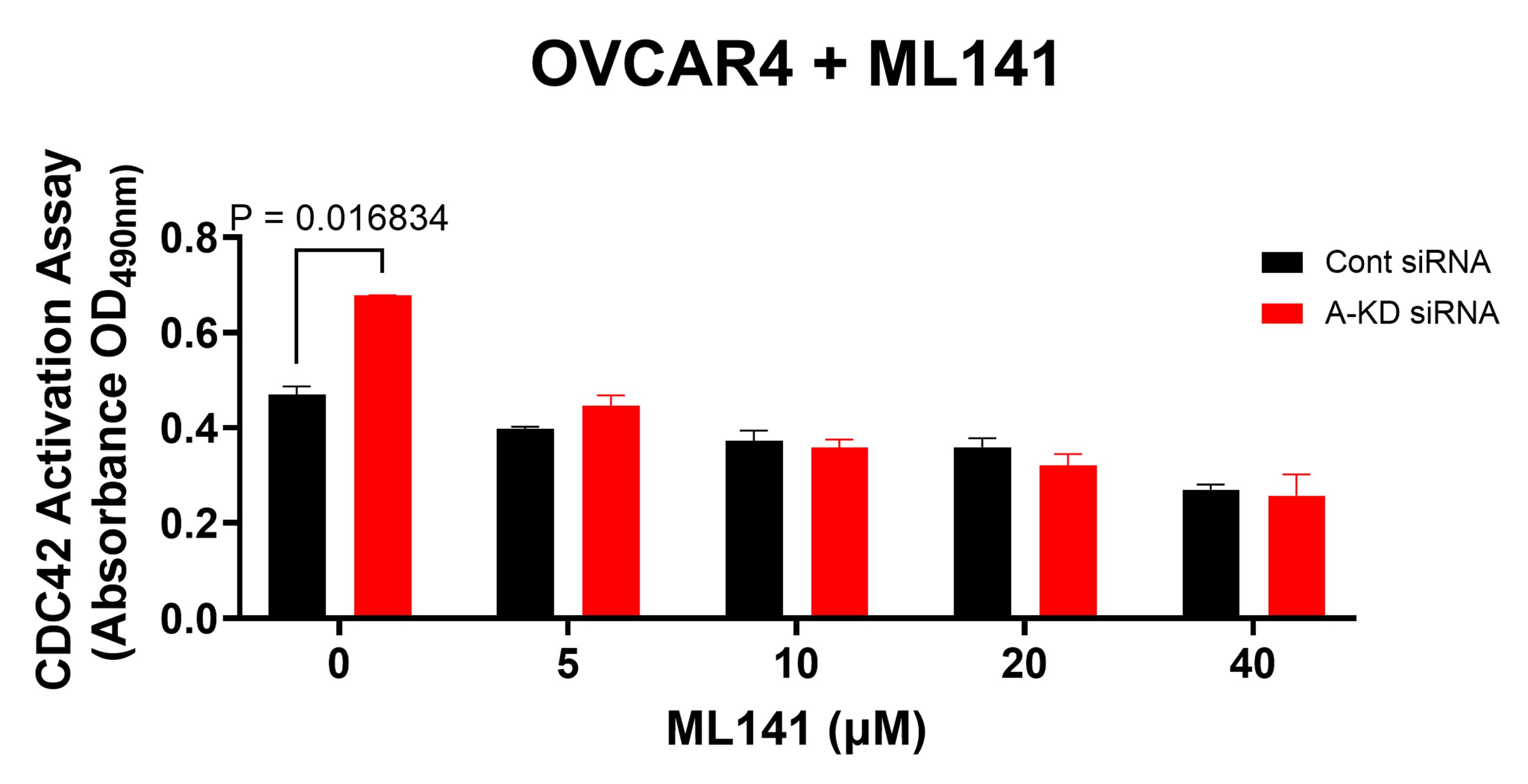

Supplement: Supplementary file 7 — Supplementary Material 7. Effect of ML141 on Cdc42 GTPase activity. ML141was used to inhibit Cdc42 GTPase activation induced by ADAMTS1 siRNA. 48 hrs post-transfection of ADAMTS1 siRNA and control siRNA. C are cells transfected with scrambled siRNA and A-KD is a representative of a pool of all three ADAMTS1 siRNAs knocked down cells. 5-10 µM ML141 decreased Cdc42 activity levels back to OVCAR4 endogenous levels. Each bar indicates mean ±SEM, derived from one experiment done in duplicate [file 12929_2026_1260_MOESM7_ESM.jpg]

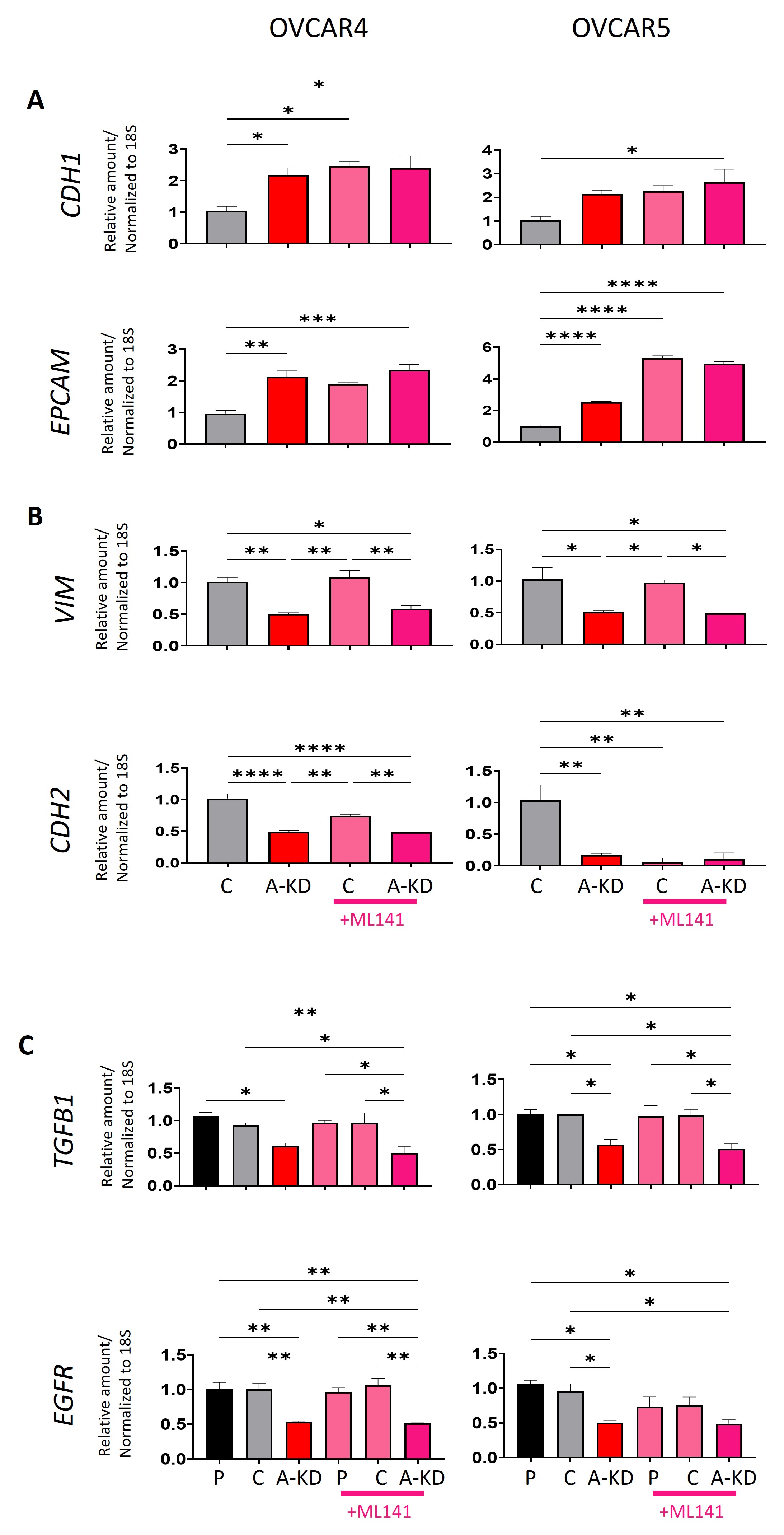

Supplement: Supplementary file 8 — Supplementary Material 8. Effect of ML141 of Cdc42 activity on epithelial plasticity. The mRNA expression EMT inducerin OVCAR4 and OVCAR5 control and ADAMTS1 siRNA treated cells for 24 h with 10µM of the Cdc42 inhibitor, was deduced by qRT-PCR. The experiment was repeated three times in triplicate. Graphs represent mean of total ± SEM. Significance is indicated by *p<0.05, and **p<0.01, one-way ANOVA [file 12929_2026_1260_MOESM8_ESM.jpg]

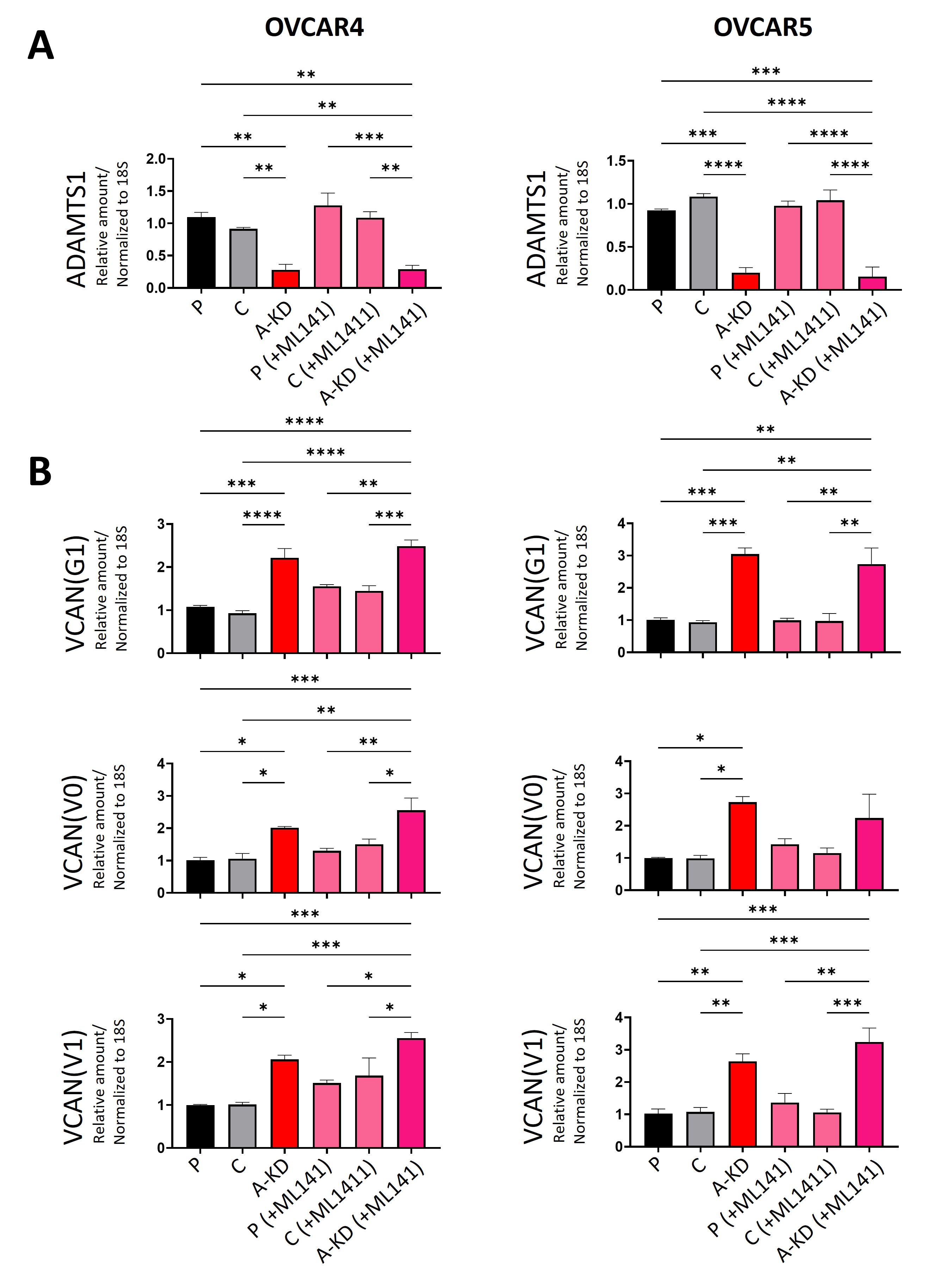

Supplement: Supplementary file 9 — Supplementary Material 9. Effect of ML141 on ADAMTS1 and VCAN expression. The mRNA expression of ADAMTS1 and VCAN in OVCAR4 and OVCAR5 control and ADAMTS1 siRNA treated cells for 24 h with 10 µM of the Cdc42 inhibitor, was assessed by qRT-PCR. The experiment was repeated three times in triplicate. Graphs represent mean of total ± SEM. Graphs represent mean of total ± SEM. Significance is indicated by *p<0.05, and **p<0.01, one-way ANOVA [file 12929_2026_1260_MOESM9_ESM.jpg]
