# Supplementary material for: Enhanced expression of ADAMTS1 in ovarian carcinomas: loss of ADAMTS1 expression instigates cellular reprogramming of extracellular matrix ensuing altered plasticity, augmented migration and attenuated adhesion
Source: J Biomed Sci. 2026 Jun 22;33:67. doi: 10.1186/s12929-026-01260-z (PMC13289130; doi:10.1186/s12929-026-01260-z)
Supplement: Supplementary file 10 — Supplementary Material 10. List of 5 Tables [file 12929_2026_1260_MOESM10_ESM.docx]

Supplementary Table 1 **Primary 0varian tissue details for immunohistochemistry study-.**

| Block # | Age | Pathologist diagnosis | Cancer Type | Tumour Type | FIGO Stage | Silverberg Classification | WHO classification | p53 staining | CA125 levels | Peripheral blood CA125 at diagnosis | Ascites Present at Diagnosis | Genetic Info. | Survived from Diagnosed till Death |
| --- | --- | --- | --- | --- | --- | --- | --- | --- | --- | --- | --- | --- | --- |
| 1 | 20 | Benign sclerosis tumour | benign | benign | -- | -- | -- | -ve | increased | 69 | no | Nil Ca | -- |
| 2 | 45 | normal OV and FT | normal | normal | -- | -- | -- | -ve | N/A | n/a | no | BRCA1+ | -- |
| 3 | 43 | benign serous cystadenoma | normal- Peutz-Jegher's Syndrome | benign | -- | -- | -- | -ve | N/A | n/a | no | Peutz-Jegher's syndrome | -- |
| 4 | 54 | Fibroma-mitotically active | benign | benign | -- | -- | -- | -ve | N/A | >7 | no | Nil Ca | -- |
| 5 | 64 | serous cystadenofibroma | Large multi cystic ovarian mass (benign) | benign | -- | -- | -- | -ve | N/A | n/a | no | Family history of Ca | -- |
| 6 | 58 | serous cystadeno-fibroma | Cyst benign | benign | -- | -- | -- | -ve | N/A | 6 | no |  | -- |
| 7 | 62 | serous cystadeno-fibroma | Large ovarian cyst (benign) | benign | -- | -- | -- | -ve | N/A | >27 | no | n/a | -- |
| 8 | 48 | simple serous cyst | Benign | benign | -- | -- | -- | -ve | N/A | no data | no | HNPCC carrier (colorectal Ca) | -- |
| 9 | 54 | serous cystadenoma - bordeline | Serous cystadenoma bordeline malignancy | bordeline | Ia | not graded | 1(Low) | -ve | N/A | >11 | not entered | Nil Ca | -- |
| 10 | 60 | serous bordeline tumour | Serous cystadenoma bordeline malignancy | bordeline | Ia | not graded | 1 (Low) | -ve | raised | 1237 | no | Nil Ca | -- |
| 11 | 37 | serous cystadenoma - bordeline, microinvasion | Ser Cys NOS | malignant | Ia | not graded | 1 (Low) | -ve | N/A | 138 | no | no (grandfather Ca pancreas) | -- |
| 12 | 49 | serous bordeline tumour | serous cystadenoma bordeline malignancy | bordeline | Ib | not graded | 1(Low) | -ve | raised | 93 | unknown | Nil Ca | -- |
| 13 | 31 | micropapillary Ser Ca | Ser Cys NOS | malignant | Ic | G1 | 1(Low) | -ve | N/A | 177 | no | grandmother gastric Ca grandfather smoker lung Ca | -- |
| 14 | 67 | serous bordeline tumour | Serous cystadenoma bordeline malignancy | bordeline | Ic | not graded | 1(Low) | -ve | N/A | 11 | no | niece and nephew Bowel Ca | -- |
| 15 | 44 | serous bordeline tumour | Papillary Ser Cys uncertain benign or malignant | bordeline | Ic | not graded | 1 (Low) | -ve | 369 | 369 | yes | n/a | -- |
| 16 | 54 | Ser Ca | Papillary Ser Cys | malignant | IIc | G3 | 2 (High) | +ve | 300 | 223 | no | mother kidney Ca, Br Ca | -- |
| 17 | 67 | Ser Ca | Papillary Ser Cys | malignant | IIc | G3 | 2 (High) | -ve | N/A | 24 | yes | sister died melanoma | -- |
| 18 | 61 | Ser Ca | Ser Cys NOS | malignant | IIc | G3 | 2 (High) | -ve | 109 | 107 | no | Nil Ca | -- |
| 19 | 72 | Ser Ca ꭞ | Papillary Ser Cys | malignant | IIc | G3 | 2 (High) | +ve | N/A | n/a | unknown | Nil Ca | -- |
| 20 | 65 | Ser Ca | Ser Cys NOS | malignant | IIb | G2 | 2 (High) | +ve | 1404 | 1404 | no | Nil ca | -- |
| 21 | 74 | Ser Ca ꭞ | Ser Cys NOS | malignant | IIIa | G3 | 2 (High) | -ve | N/A | 104 | yes | BRCA2+ve | -- |
| 22 | 37 | micropapillary Ser Ca | Papillary Ser Cys | malignant | IIIc | G1 | I (Low) | -ve | N/A | n/a | yes | No (Nil, father liver Ca --drinker) | -- |
| 23 | 45 | Ser Ca | Papillary Ser Cys | malignant | IIIc | G1 | I (Low) | -ve | N/A | 585 | yes | n/a | -- |
| 24 | 42 | Ser Ca | Papillary Ser Cys | malignant | IIIc | G2 | 2 (High) | -ve | 1200 | n/a | not entered | Family history of Ca | -- |
| 25 | 43 | Ser Ca | Papillary Ser Cys | malignant | IIIc | G2 | 2 (High) | -ve | N/A | 428 | yes | n/a | 3 years 7months |
| 26 | 76 | Ser Ca | Papillary Ser Cys | malignant | IIIc | G3 | 2 (High) | +ve | N/A | 4831 | yes | Nil Ca | 4 years 1months |
| 27 | 54 | Ser Ca | Papillary Ser Cys | malignant | IIIc | G3 | 2 (High) | -ve | 1230 | 1113 | yes | BRCA2 +ve | -- |
| 28 | 62 | Ser Ca | Carcinoma NOS | malignant | IIIc | G3 | 2 (High) | +ve | N/A | 3058 | yes | BRCA1 +ve | 2 years 7months |
| 29 | 56 | Ser Ca | Ser Cys NOS | malignant | IIIc | G3 | 2 (High) | -ve | increased | 3025 | yes | -ve, Nil Ca | 2 years 5months |
| 30 | 38 | Ser Ca | Ser Cys NOS | malignant | IIIc | G3 | 2 (High) | -ve | increased | 957 | yes | Nil Ca | -- |
| 31 | 60 | Ser Ca | Serous surface papillary carcinoma | malignant | IIIc | G3 | 2 (High) | +ve | 374 | 397 | yes | BRCA2 +ve | 4 years 7months |
| 32 | 59 | Ser Ca | Papillary Ser Cys | malignant | IV | G2 | 2 (High) | +ve | N/A | 3187 | yes | sister with Ca | 6 years 10months |
| 33 | 61 | Ser Ca | Ser Cys NOS | malignant | IV | G2 | 2 (High) | -ve | raised | 333 | n/a | BRCA2 carrier | 3years 11months |

ꭞPrimary site is FT; Ser Ca = Serous Carcinoma; Ser Cys= Serous Cystadenocarcinoma; -ve= negative; +ve= positive; OV = ovaries; FT = Fallopian Tube; Ca = cancer; n/a = not available; G1 = grade 1; G2 = grade 2; G3 = grade 3

**Supplementary Table 2.** Clinicopathological characteristics of serous ovarian cancer TMA cohort

| **Primary High grade serous ovarian carcinomas (n=115)** | | |
| --- | --- | --- |
| **Age at Diagnosis (years)** | **Median (range)** | **60 (24-86)** |
| Histological Grade | Grade 2  Grade 3 | 19  96 |
| FIGO stage | Stage II  Stage III  Stage IV | 1  107  7 |
| ADAMTS1 H-score Epithelial | Median (range)  Mean ± SD | 123.4 (0-299.0)  140.2 ± 103.0 |
| ADAMTS1 H-score Stroma | Median (range)  Mean ± SD | 8.8(0-188.4)  29.8 ± 39.6 |
| Recurrence | No  Yes  Unknown | 24  80  11 |
| Cause of Death | Ovarian cancer  Other cause  Alive  Lost to follow-up | 75  11  28  1 |
| **Metastatic High grade serous ovarian carcinomas (n=42)** | | |
| **Age at Diagnosis (years)** | **Median (range)** | **70 (46-86)** |
| Histological Grade | Grade 2  Grade 3 | 7  35 |
| FIGO stage | Stage II  Stage III  Stage IV | 0  39  3 |
| ADAMTS1 H-score Epithelial | Median (range)  Mean ± SD | 273.4(63.2-300)  243.6 ± 65.6 |
| ADAMTS1 H-score stroma | Median (range)  Mean ± SD | 67.8(8.7-221.8)  92.0 ± 62.1 |
| Recurrence | No  Yes  Unknown | 10  27  5 |
| Cause of Death | Ovarian cancer  Other cause  Alive  Lost to follow-up | 30  3  8  1 |

Supplementary Table 3  **Description of chemo-naïve patients recruited for ascites samples study**

| Ascites Samples | Sample or Cell type used  for the study | Diagnosis | Primary site | FIGO Stage | Silverberg Grade | Age | Time of first recurrence (after completion of first line of chemotherapy) | Time of sample collection (after diagnosis) | Treatment received before  the collection of ascites |
| --- | --- | --- | --- | --- | --- | --- | --- | --- | --- |
| As59 | Ep & Mes | Ser Cys NOS | OV | IIc | *G3 | 64 | NA | AD | None |
| As67 | Mes | adenocarcinoma NOS | MS (not sure, possible OV with distal metastasis to gastrointestinal/ colonic) | Unk | Not graded | 64 | NA | AD | None |
| As68 | Ep | Ser Cys | OV | IV | G3 | 72 | NA | AD | None |
| As71 | Ep & Mes | Ser Cys NOS | MS (genital tract -Female NEC | Unk | not graded | 62 | NA | AD | None |
| As80 | Ep & Mes | Ser Car | FT | Unk | G3 | 53 | NA | AD | None |
| As81 | Ep & Mes | Serous Papillary Carcinoma | OV | IIIc | G3 | 68 | NA | AD | None |
| As82 | Ep & Mes | Ser Cys | OV | IIIc | *G3 | 48 | NA | AD | None |
| As85 | Ep & Mes | Serous cystadenocarcinoma NOS | FT | Unk | G3 | 67 | NA | AD | None |
| As99 | Ep & Mes | Ser Cys NOS | Genital tract | IIIc | G3 | 64 | NA | AD | None |

^*^G3 = Poorly differentiated; NA =Not applicable; AD = After diagnosis, before treatment; Ser Ca = Serous Carcinoma; Ser Cys = Serous Cystadenocarcinoma; OV = ovary; Ep = epithelial; Mes = mesenchymal; MS = multiple sites, FT = Fallopian Tube.

Supplementary Table 4 **Other Cell Lines used in the study.**

| Cell Lines | Origin and Histology | Culture Medium |
| --- | --- | --- |
| FT282 | Normal epithelium Fallopian tube derived (Karst and Drapkin, 2012) | DMEM: F12 (1:1) This cell line was supplemented with Ultroser™ G serum substitute (PALL, Life Sciences, NY, USA) instead of FBS. |
| AOCS1 | Ascites; Material from a HGSC patient after second relapse, patient pre-treated CA and CIS (Milagre et al., 2015). | RPMI |
| CAOV3 | Tumour/Ovary derived from a pre-treated patient [CPA/DOX/ FU]); Serous/ adenocarcinoma (Likely High-grade serous)(Buick et al., 1985) | DMEM |
| COV318 | Ascites, Serous/ Fallopian Tube derived (van den Berg-Bakker et al., 1993) | DMEM |
| HEY | Derived from a human ovarian cancer xenograft (HX-62) originally grown from a peritoneal deposit of a patient with moderately differentiated papillary cystadenocarcinoma of the ovary. prior to chemotherapy treatment; Serous (Low grade serous) (Buick et al., 1985) | RPMI |
| JHOS-2 | Tumour/ epithelial ovarian carcinoma; Serous cystadenocarcinoma (Likely High-grade serous)(Yamada et al., 1999) | DMEM: F-12 (1:1) |
| JHOS-4 | Tumour/ High grade ovarian serous cyst adenocarcinoma; (Likely High-grade serous) (RIKEN BioRessource Center, Cat#RCB1678) | DMEM: F-12 (1:1) |
| OVCA433 | Ascites fluid prior to chemotherapy treatment ; Papillary serous cystadenocarcinoma (Bast et al., 1981) | DMEM: MCDB (1:1) |
| OVCA429 | Ascites; Serous(Bast et al., 1981) | DMEM: MCDB (1:1) |
| OVCAR4 | Ascites fluid derived from pre-treated patient [CPA/DOX/CIS] ovarian adenocarcinoma (Hamilton et al., 1984) (Likely High-grade serous) | RPMI |
| OVCAR5 | Ascites fluid prior to chemotherapy treatment (Johnson et al 1997); High-grade ovarian adenocarcinoma (Hamilton et al., 1984; Johnson et al., 1997) | RPMI |
| OVKATE | Metastatic deposit; ovarian carcinoma, stage IIIc, CAP(3) and EP(3) treatment done previously (Yanagibashi et al., 1997) | RPMI |
| SKOV3 | Ascites from a pre-treated patient [TT]; adenocarcinoma metastasis ascites (clear cell; Unlikely High-grade serous)(Fogh et al., 1977) | DMEM |
| TOV21G | Tumour/ grade 3, stage III, primary malignant adenocarcinoma; Clear cell (Hyper mutated cell line)(Mes-Masson and Provencher, 1998) | DMEM: MCDB (1:1) |

*CA= Carboplatin;* *CPA=cyclophosphamide; CIS=cisplatin;* *DOX=doxorubicin;* *FU=5-fluorouracil;* *TT=Thiotepa; CAP = Cyclophosphamide, Adriamycin and Cisplatin;* *EP= etoposide and cisplatin.*

Supplementgary Table 5 **Primers used in this study.**

| Gene symbol | Sequences (5' - 3') | Accession # | Product size (bp) | Fluorescence Capture (°C) |
| --- | --- | --- | --- | --- |
| 18S | F GTAACCCGTTGAACCCCATT  R CCATCCAATCGGTAGTAGCG | NR_003286.1 | 153 | 78 |
| ADAM10 | F TGCTGCTTCGATGCAAATCAACC  R TGCACAGTCTGAATCATCCCGAC | NM_001110.3 | 147 | 78-80 |
| ADAM12/ Meltrin-alpha | F ACCGAGAGTTTCAGAGGCAA  R CTGGTGAATGGGTCCTGACT | NM_003474.6 | 174 |  |
| ADAM17/TACE | F AGAAACCTAACCACCTACCT  R CAACCTCAGCCTCTCCAAGT | NM_003183 | 229 | 78 |
| ADAMTS1 | F CCTCTGTCTGTGTGCAAGGA  R GAGCCGCTGTACCTCAAGAC | NM_006988.4 | 349 | 78 |
| ADAMTS4 | F CTGACCACTTTGACACAGCC  R GTGCTCAAAGGCCCATTCAA | NM_005099.6 | 238 |  |
| ADAMTS5 | F ACTACGATGCAGCTATCCTGT  R CATGGGAGAGGCCAAGTAAA | NM_007038.5 | 189 |  |
| ADAMTS9 | F CGAAAAACCTGCCGTAATGT  R TCAGAGTCTCCATGCACCAG | NM_182920.1 | 191 | 78 |
| ADAMTS15 | F CAACATCGTTGTGGTCAAGG  R GGTCACACATGGTACCCACA | NM_139055.4 | 236 |  |
| ADAMTS16 | F GGAATAACTCAGCCTGCACG  R AGTCGAAAGTAGTGCCCGAA | NM_139056.4 | 234 |  |
| E-CAD/ CDH1 | F GGCACAGATGGTGTGATTACAG  R GTCCCAGGCGTAGACCAAGAAA | NM_004360.3 | 81 | 75 |
| EpCAM | F CGTCAATGCCAGTGTACTTCAGTTG  R TCCAGTAGGTTCTCACTCGCTCAG | NM_002354.2 | 301 | 80-83 |
| EGFR | F CCAGTATTGATCGGGAGAGC  R CCAAGGACCACCTCACAGTT | NM_005228.5 | 234 |  |
| ITGA3 | F GCCTGCCAAGCTAATGAGAC  R AGAAGCTTTGTAGCCGGTGA | NM_002204.4 | 247 |  |
| ITGAV | F AGGAGAAGGTGCCTACGAAG  R GCACAGGAAAGTCTTGCTAAGG | NM_002210.5 | 105 |  |
| ITGB1 | F ATCCCAGAGGCTCCAAAGAT  R CTAAATGGGCTGGTGCAGTT | NM_133376 | 409 |  |
| FBLN1 | F GGAATCCAAAGAATGCAGGA  R ACTGGTAGCCAACCATGAGG | NM_006487.3 | 239 |  |
| L1CAM | F GCCAAAGGAGACAGTGAAGC  R GCGTGGCAGATGTAGTCTGA | NM_000425.5 | 219 |  |
| MKI67/Ki67 | F TTGGTACTGGGGGAGGGAGA  R TGGGAGGCGAAAAAGTAAAA | NM_002417.4 | 188 | 78 |
| MMP-2 | F TTGACGGTAAGGACGGACTC  R ACTTGCAGTACTCCCCATCG | NM_004530.4 | 153 | 80-83 |
| MMP-9 | F TTGACAGCGACAAGAAGTGG  R GCC ATTCACGTCGTCCTTAT | NM_004994.2 | 179 | 83-85 |
| MT1-MMP/ MMP-14 | F GCTCCGAGGGGAGATGTTTG  R CAGCTCCTTAATGTGCTTGGG | NM_004995.2 | 235 | 83 |
| N-CAD/ CDH2 | F AAACAGCAACGACGGGTTAG  R CTTAGGATTGGGGGCAAAAT | NM_001792.3 | 195 | 78 |
| RECK | F GAACTGGCTATTGCCTTGGA  R ATTCTCGGCAGTTTGTGTGA | NM_021111.2 | 175 | 79 |
| SDC4 | F GTCTGGCTCTGGAGATCTGG  R GTCTGGCTCTGGAGATCTGG | NM_002999.4 | 224 |  |
| TGFB1-Ligand | F CAACAATTCCTGGCGATACCT  R GCTAAGGCGAAAGCCCTCAAT | NM_000660 | 136 | 79-82 |
| TIMP-1 | F TGACATCCGGTTCGTCTACA  R GTTTGCAGGGGATGGATAAA | NM_003254.2 | 248 | 85 |
| TIMP-2 | F CCGCAACAGGCGTTTTGCAA  R TCACTTCTCTTGATGCAGGC | NM_003255.4 | 494 | 85 |
| TIMP-3 | F TTCTGCAACTCCGACATCGT  R ATGCAGGCGTAGTGTTTGGA | NM_000362.4 | 452 | 83 |
| TWIST1 | F GTCCGCAGTCTTACGAGGAG  R CCAGCTTGAGGGTCTGAATC | NM_000474.3 | 159 | 88 |
| VCAN-G1 domain | F TTACCGCTGTGACGTCATGT  R CTGCGTCACACTGCTCAAAT | NM_004385.5 | 215 |  |
| VCAN-V0 | F CAAGCTTACACAGCCAACAAGACCA  R ACTCTAGAGGCCACGCCTAGCTTCTGCAGC | NM_003380.3 | 436 |  |
| VCAN-V1 | F CGGGATCCGGGGTGAGAACCCTGTATCG  R ACTCTAGAGGCCACGCCTAGCTTCTGCAGC | NM_003380.3 | 375 |  |
| VCAN-V2 | F TCAGAGAAAATAAGACAGGACCTGATC  R CATACGTAGGAAGTTTCAGTAGGATAACA | NM_003380.3 | 135 |  |
| VCAN-V3 | F GGCTTTGACCAGTGCGATTAC  R CCAGCCATAGTCACATGTCTC | NM_003380.3 | 429 |  |
| VIM | F CCTACAGGAAGCTGCTGGAA  R GGTCATCGTGATGCTGAGAA | NM_003380.3 | 198 | 75 |
